# Solid state frustrated Lewis pair chemistry†
†Electronic supplementary information (ESI) available: Additional experimental details, further spectral and crystallographic data, additional data from the solid state NMR and theoretical studies. CCDC 1515080–1515082. For ESI and crystallographic data in CIF or other electronic format see DOI: 10.1039/c8sc01089g


**DOI:** 10.1039/c8sc01089g

**Published:** 2018-04-23

**Authors:** Long Wang, Gerald Kehr, Constantin G. Daniliuc, Melanie Brinkkötter, Thomas Wiegand, Anna-Lena Wübker, Hellmut Eckert, Lei Liu, Jan Gerit Brandenburg, Stefan Grimme, Gerhard Erker

**Affiliations:** a Organisch-Chemisches Institut , Westfälische Wilhelms-Universität Münster , Corrensstraße 40 , 48149 Münster , Germany . Email: erker@uni-muenster.de; b Institut für Physikalische Chemie , Graduate School of Chemistry , Westfälische Wilhelms-Universität Münster , Corrensstraße 30 , 48149 Münster , Germany . Email: eckerth@uni-muenster.de; c Laboratorium für Physikalische Chemie , ETH Zürich , Vladimir-Prelog-Weg 2 , 8093 Zürich , Switzerland; d Institute of Physics in Sao Carlos , University of Sao Paulo , CEP 369 , Sao Carlos SP 13566-590 , Brazil; e London Centre for Nanotechnology , University College London , 17-19 Gordon Street , London , WC1H 0AH , UK; f Mulliken Center for Theoretical Chemistry , Institut für Physikalische und Theoretische Chemie , Universität Bonn , Beringstraße 4 , 53115 Bonn , Germany . Email: grimme@thch.uni-bonn.de

## Abstract

In solution the PCy_3_/B(C_6_F_5_)_3_ pair is rapidly deactivated by nucleophilic aromatic substitution. In the solid state deactivation is effectively suppressed and the active frustrated phosphane/borane Lewis pair splits dihydrogen or adds to sulfur dioxide. A variety of phosphane/B(C_6_F_5_)_3_ pairs have been used to carry out active FLP reactions in the solid state. The reactions were analyzed by DFT calculations and by solid state NMR spectroscopy. The solid state dihydrogen splitting reaction was also carried out under near to ambient conditions with suspensions of the non-quenched phosphane/borane mixtures in the fluorous liquid perfluoromethylcyclohexane.

## Abstract 2

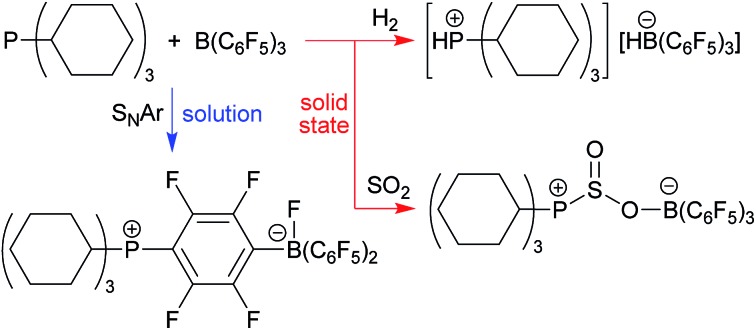
In solution the PCy_3_/B(C_6_F_5_)_3_ pair is rapidly deactivated by nucleophilic aromatic substitution. In the solid state (or in a fluorous liquid), however, it serves as an active frustrated Lewis pair to effectively split dihydrogen.

## Introduction

Lewis acids and bases when brought together in solution typically undergo rapid formation of strong adducts. Similar to the neutralization reaction of Brønsted acids and bases this leads to an annihilation of the typical Lewis acid and Lewis base properties.^1^,^2^ This situation can be changed if one effectively hinders the Lewis pair from the neutralizing adduct formation, *e.g.* by electronic means^3^ or, more commonly, by attaching very bulky substituents at the core atoms of the pair.^4^–^6^ This invariably leads to situations where active Lewis acids and active Lewis bases are present in a solution at the same time, opening possibilities for cooperative reactions with added substrates. Such “frustrated Lewis pairs” (FLPs)^7^ have very successfully been used within the last decade for small molecule binding and activation, most notably among this the metal-free splitting and activation of dihydrogen.^8^,^9^ A number of new reaction types have been found in this way^10^,^11^ and a great variety of different FLPs have been devised, characterized and their reactions reported.^12^–^23^ The vast majority of them relies on avoiding the effective neutralizing Lewis acid/Lewis base adduct formation by steric hindrance, and this has led to the discovery and development of a great variety of interesting reactions in solution.^24^–^26^

We thought that we should not confine ourselves to searching for FLP reactions in the liquid phase. One may safely assume that large molecules in the solid state are more or less rigidly confined to their positions in the crystal lattice. Therefore, Lewis acids and Lewis bases should effectively be hindered from adduct formation or other deactivating reaction pathways even in the absence of efficient steric hindrance by their substituents as long as we keep them in the solid state. This may actually set the scene for possibly finding new frustrated Lewis pairs and, consequently, new FLP reactions, by exposing such Lewis pairs to suitable reagents in the solid state. We have tried this principle and found that FLP chemistry can be done in this way.

There had been some reports about frustrated Lewis pair related behavior at certain heterogeneous catalysts. In these cases the active sites were part of the catalytic solid. Activation of small molecules at such systems had been achieved either thermally or by photolysis.^27^–^29^ There have also been a few reports about heterogenized Lewis acids, bonded to suitable supports, that have been employed in FLP type reactions.^30^ Our here reported case is distinctly different: we have employed solid physical mixtures of phosphane Lewis bases with the strong B(C_6_F_5_)_3_ boron Lewis acid and reacted them under suitable conditions with selected small molecules. Activation occurred and the phosphane/borane pair became consumed with selective formation of the FLP reaction products. First examples of this FLP development will be described below in this account.

## Results and discussion

**Scheme 1:**
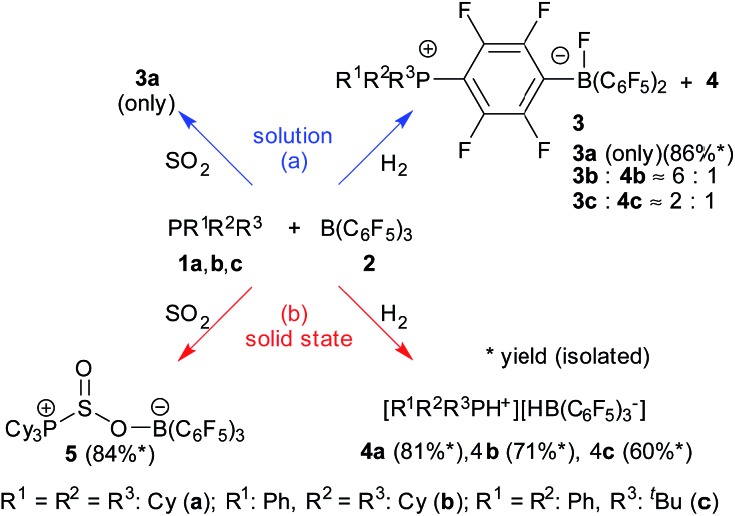
Reactions of the PR^1^R^2^R^3^/B(C_6_F_5_)_3_ FLP systems in a dihydrogen and SO_2_ atmosphere, respectively, in solution (a) and in the solid state (b). [Cy: cyclohexyl].

Tricyclohexylphosphane (**1a**) and tris(pentafluorophenyl)-borane (**2**) represent a Lewis base/Lewis acid pair that is known to react rapidly in solution. It undergoes a nucleophilic aromatic substitution by the phosphane at the *para*-position of a C_6_F_5_ group of the borane^4^,^31^ to eventually yield the reaction product **3a**.^7^,^32^ This favorable S_N_Ar reaction eliminates any FLP reactivity of the **1a**/**2** pair very effectively in solution. Even in the presence of 50 bar H_2_ no significant hydrogen splitting is observed, the formation of **3a** even prevails under these conditions. We isolated this compound in 86% yield and confirmed its formation by X-ray diffraction and by NMR spectroscopy [Scheme 1a (H_2_); for details see the ESI†].^32^

The situation is drastically different in the solid state: we mixed equimolar quantities of PCy_3_ (**1a**) and B(C_6_F_5_)_3_ (**2**) and exposed it in a glass vial inside a steel autoclave to 50 bar of dihydrogen for a total of 10 days with constant agitation by a Teflon coated magnetic stirring bar. After this time a sample was taken and dissolved in d_2_-dichloromethane. The NMR analysis revealed the hydrogen splitting product [HPCy_3_^+^][HB(C_6_F_5_)_3_^–^] (**4a**) had been formed as the by far major product [Scheme 1b (H_2_)]. Only negligible if any amounts of the S_N_Ar product **3a**, which would have been formed readily from any residual PCy_3_/B(C_6_F_5_)_3_ upon dissolving in dichloromethane, were present in the *in situ* samples. The phosphonium/hydridoborate product **4a** was identified by its typical ^31^P (*δ* 33.2, ^1^*J*_PH_ ∼443 Hz) and ^11^B (*δ* –25.3, ^1^*J*_BH_ ∼92 Hz) NMR signals with correlated ^1^H NMR features at *δ* 5.15 (dq, ^1^*J*_PH_ = 444.0 Hz, ^3^*J*_HH_ = 4.1 Hz) and *δ* 3.59 (br 1 : 1 : 1 : 1 q, [B]H), respectively. Workup of a representative sample eventually furnished the salt **4a** isolated in 81% yield on a 100 mg scale. Crystallization from dichloromethane/pentane gave single crystals which were used to confirm the formation of the FLP H_2_ splitting product under these special conditions by X-ray diffraction (for details see the ESI†).^33^ The salt **4a** is an active reducing agent. Its reaction with the bulky *N*-phenyl-4-methylacetophenon-imine gave the respective secondary amine reduction product (24 h at 70 °C, 86% conversion, for details see the ESI†).

**Fig. 1:**
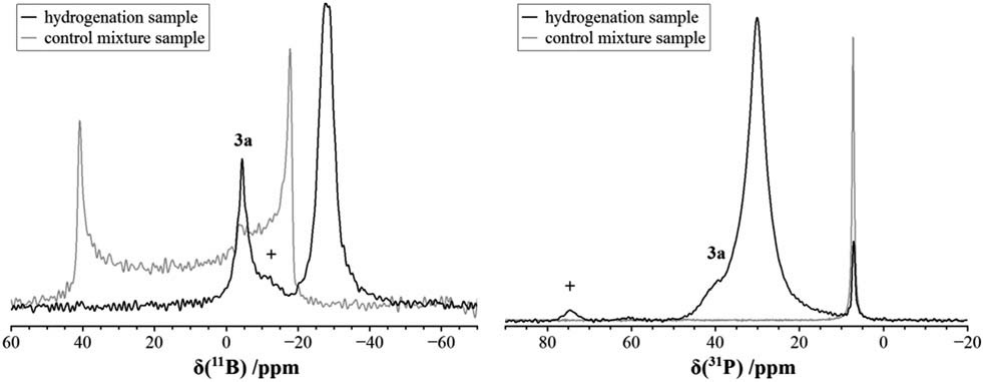
^11^B{^1^H} MAS (left) and ^31^P{^1^H} CPMAS NMR spectra (right) of the B(C_6_F_5_)_3_/PCy_3_ control mixture sample (grey trace) and the hydrogenation sample (black trace) from the solid state reaction with H_2_. Minor side products are labelled by +.

We carried out an ample characterization of the solid product material directly (*i.e.* without ever dissolving it) by solid state NMR spectroscopy. Evidence for the solid state hydrogenation of the PCy_3_/B(C_6_F_5_)_3_ mixture comes from the ^31^P and ^11^B MAS NMR data (Fig. 1). In the ^31^P NMR spectrum we recognize the starting material at 7.1 ppm, whereas the phosphonium ion gives a broad signal at 30.1 ppm. In the ^11^B MAS NMR spectrum the signal of the free B(C_6_F_5_)_3_ gives rise to the previously documented second-order quadrupolar lineshape^34^ whereas after the hydrogenation a much narrower signal appears at the isotropic chemical shift of –24.9 ppm after applying the correction for the second-order quadrupolar shift (see ESI†). In addition, the spectrum reveals the presence of a minor amount of the substitution product at –2.4 ppm. The latter is the only product formed when the reaction is carried out in solution. The complete NMR characterization of the substitution product both in solution and the solid state is given in the ESI.† In a control experiment, the formation of only a minor amount of the substitution product **3a** was observed in the solid state NMR spectra of a PCy_3_/B(C_6_F_5_)_3_ mixture subjected to identical reaction conditions in the absence of H_2_.

**Fig. 2:**
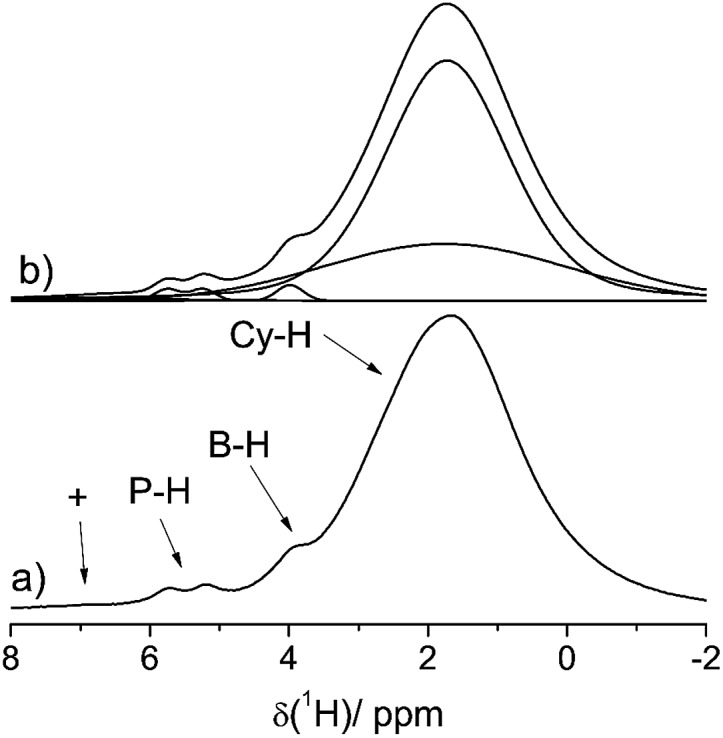
^1^H MAS NMR spectrum of a reaction mixture after the solid state reaction of B(C_6_F_5_)_3_/PCy_3_ with H_2,_ acquired using the EASY scheme for probe and rotor cap background suppression measured at 20.0 T and a MAS frequency of 60.0 kHz (a) and corresponding line shape simulation (b). + marks a small signal remaining from the rotor cap.

Fig. 2 shows the ^1^H MAS NMR spectrum of the FLP–H_2_ adduct **4a** acquired at 20.0 T and a MAS spinning frequency of 60.0 kHz with the EASY scheme for suppression of background signals from the MAS probe and the MAS rotor cap.^35^ Under these conditions, the strong ^1^H–^1^H dipolar couplings are sufficiently suppressed, even though residual line broadening is still detected owing to higher order terms in the Hamiltonian which are not fully eliminated even at 60.0 kHz.^36^ A distinct doublet (*J*(^1^H–^31^P) ∼ 430 Hz) can be identified at 5.5 ppm which is assigned to P-bound hydrogen, whereas the singlet at 4.0 ppm is assigned to the B-bound hydrogen (the expected multiplet is not resolved in this case).

**Fig. 3:**
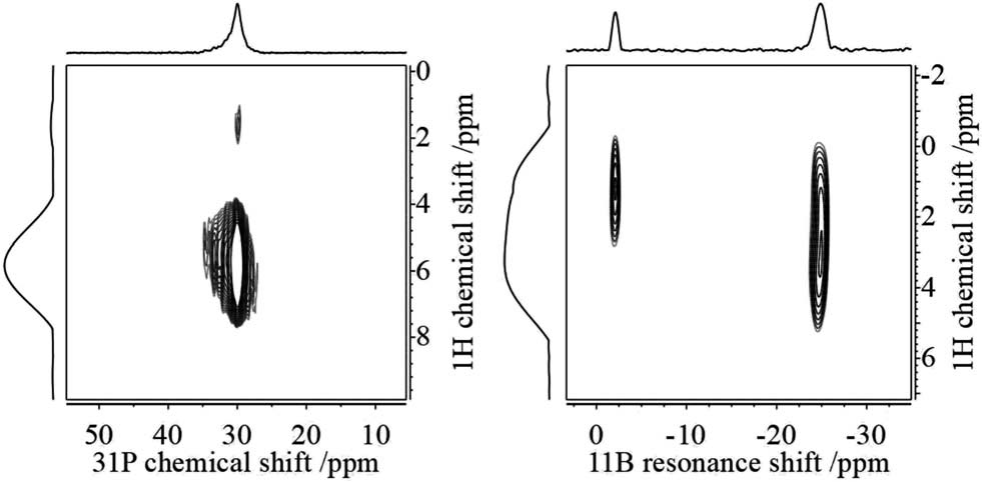
^1^H/^31^P (left) and ^1^H/^11^B (right) HETCOR spectra of a HB(C_6_F_5_)_3_^–^/HPCy_3_^+^ mixture **4a** after the solid state reaction with H_2_. Cross-peaks denote the B–H and P–H correlations. The additional ^1^H/^11^B crosspeak seen in the right part of the figure at (1.3/–5.2) ppm arises from the substitution product **3a**.

This assignment is supported by ^11^B{^1^H} and ^31^P{^1^H} heteronuclear correlation experiments (Fig. 3) which show intense cross-peaks linking these resonances to the corresponding ^31^P and ^11^B NMR signals of the solid state hydrogenation product. Further support for this assignment comes from ^1^H{^11^B} REAPDOR experiments. The obtained peak assignments are in agreement with solution state NMR data (*vide supra*), as well as DFT computations of ^1^H NMR chemical shifts for the isolated cationic H–PCy_3_^+^ and anionic H–B(C_6_F_5_)_3_^–^ species (5.4 and 4.5 ppm on a B3-LYP/def2-TZVP level of theory, respectively). The absence of an encounter complex is proven by ^31^P{^11^B} REAPDOR and ^11^B{^31^P} REDOR experiments (see the ESI†). As previously discussed, such experiments can probe the B···P distance by measuring the strength of the heteronuclear ^11^B–^31^P dipole–dipole interactions in both FLPs and their reaction products.^37^ In the present material, no dephasing was observed over a dipolar mixing time of ∼5 ms. Comparing these experimental data with corresponding two-spin simulations we can conclude that the boron–phosphorus distance must be larger than 600 pm. Thus, all the experimental data are consistent with well-separated phosphonium and borate ions.

The reaction of the phosphane PPhCy_2_ (**1b**) with the Lewis acid B(C_6_F_5_)_3_ (**2**) in the solid state proceeds similarly. The reaction was carried out analogously as the one described above (r.t., 50 bar H_2_, 3 days). Our analysis of a product sample dissolved in CD_2_Cl_2_ revealed almost exclusive formation of the dihydrogen FLP splitting product **4b** [Scheme 1b (H_2_)]. It shows a characteristic ^1^H NMR [P]H doublet at *δ* 6.13, ^1^*J*_PH_ = 459.3 Hz (^31^P: *δ* 30.6) and a broad 1 : 1 : 1 : 1 intensity [B]H quartet at *δ* 3.64 (^11^B: *δ* –25.3, d, ^1^*J*_BH_ ∼94 Hz).

Keeping the PPhCy_2_/B(C_6_F_5_)_3_ mixture (**1b**/**2**) in CD_2_Cl_2_ solution for 12 hours under H_2_ (50 bar) gave a different result. The NMR analysis showed the formation of a *ca.* 1 : 6 mixture of the salt **4b** with the substitution product **3b**. [Scheme 1a (H_2_)]. The zwitterionic phosphonium/fluoroborate product **3b** was isolated from a separate experiment as a white solid in 82% yield. It shows a ^11^B NMR doublet (^1^*J*_BF_ ∼70 Hz) at *δ* –0.6 and a sharp ^31^P NMR signal at *δ* 33.6. The ^19^F NMR spectrum shows the [B]F resonance at *δ* –192.9, two signals of the bridging C_6_F_4_ group and three resonances (*o*, *p*, *m*) of the remaining B(C_6_F_5_)_2_ unit with a Δ*δ*^19^F_*m*,*p*_ shift difference of 4.9 ppm, which is typical for a borate type structure (for details and the depicted NMR spectra see the ESI†).

In this case the nucleophilic aromatic substitution by the markedly less nucleophilic phosphane PPhCy_2_ (**1b**) relative to PCy_3_ (**1a**) seems to allow the FLP reaction to compete as a minor pathway in solution. This becomes continued in the reaction of the much less nucleophilic phosphane PPh_2_(^*t*^Bu) (**1c**) which in solution together with the B(C_6_F_5_)_3_ Lewis acid gave a ratio of the S_N_Ar and FLP products of **3c** : **4c** ∼ 2 : 1 under our typical reaction conditions (r.t., CH_2_Cl_2_ solution, 12 hours, 50 bar H_2_). [Scheme 1a (H_2_)]. Without H_2_ only the substitution product **3c** was formed in solution (isolated in 76% yield, see the ESI† for its characterization). On the contrary, the reaction of the PPh_2_(^*t*^Bu)/B(C_6_F_5_)_3_ Lewis base/Lewis acid mixture in the solid state (r.t., 50 bar H_2_, 3 days) gave almost pure H_2_-splitting product **4c**, [Scheme 1b (H_2_)] [^31^P NMR: *δ* 31.4 (^1^*J*_PH_ ∼474 Hz), ^11^B: *δ* –25.2 (^1^*J*_BH_ ∼91 Hz), ^19^F: Δ*δ*^19^F_*m*,*p*_ = 3.0 ppm] which we isolated from the workup procedure involving recrystallization from CH_2_Cl_2_/pentane in 60% yield (details of the characterization of the compounds **3c** and **4c** see the ESI†).

**Fig. 4:**
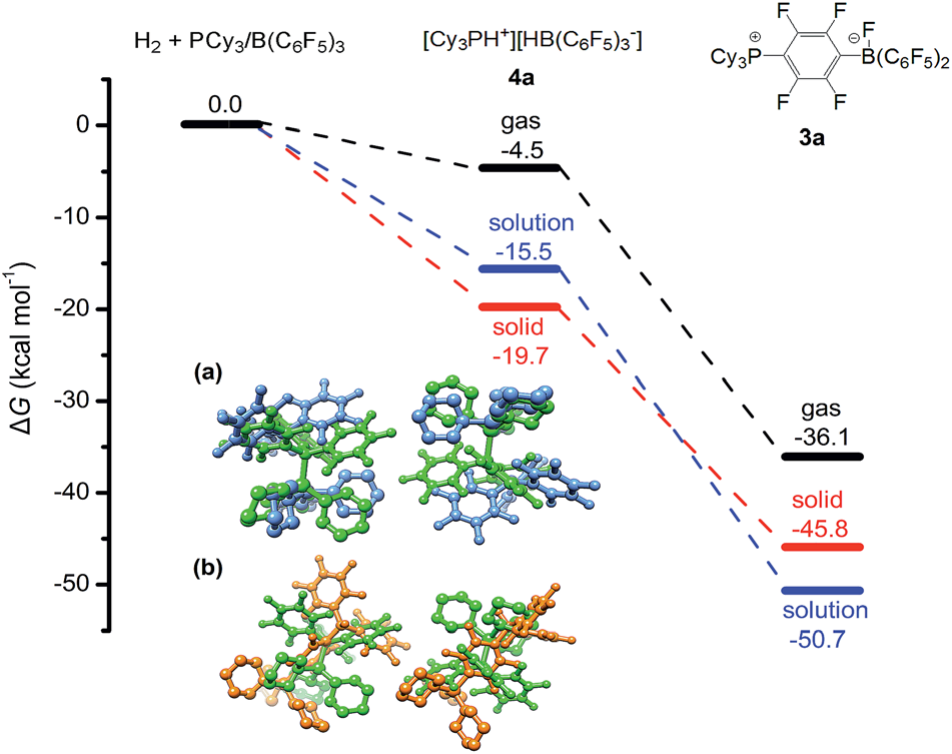
Calculated Gibbs free energies for the reaction of PCy_3_/B(C_6_F_5_)_3_ with H_2_ in the solution (toluene) and in the solid state (see the ESI† for computational details). All energy values are given in kcal mol^–1^. Inserted figures are the overlays of the HF-**3c** calculated crystal structures of PCy_3_/B(C_6_F_5_)_3_ (green) and [HPCy_3_^+^][HB(C_6_F_5_)_3_^–^] (blue) (a), and crystal structures of PCy_3_/B(C_6_F_5_)_3_ (green) and the S_N_2Ar product **3a** (orange) (b). Hydrogen atoms except P–H and B–H are omitted for clarity.

Quantum chemical simulations were used to investigate the mechanistic details of the different FLP reactivities in all three states of matter, *i.e.* gas, liquid, and solid phase. The FLP/H_2_ thermochemistry is for the first time investigated here in the solid state using relatively high-level periodic quantum chemistry methods. We employed a hierarchy of theoretical methods, ranging from semi-empirical tight-binding Hamiltonians to accurate London dispersion corrected hybrid density functionals.^38^–^45^ More discussion of methodological points and computational details can be found in our previous benchmark study^46^ and in the ESI.† The main representative results of the PCy_3_/B(C_6_F_5_)_3_ FLP are shown in Fig. 4.

All shown reactions are exergonic, and both the solvent and the crystal phase stabilize the products **4a** and **3a** by 10 to 15 kcal mol^–1^ compared to the gas phase. In this regard the FLP reaction to **4a** is in fact thermodynamically feasible. However, the competing product **3a** is in solution significantly preferred over **4a** (–50.7 kcal mol^–1^*vs.* –15.5 kcal mol^–1^) and due to the expected high mobility it can readily react and prevent the desired FLP reaction. In contrast, the crystal field provides much more pronounced energy barriers, which kinetically stabilizes the reactant and enables the targeted reaction to product **4a**. These higher energy barriers can be rationalized by a simple geometric comparison (inset of Fig. 4). Apparently, the solid state reaction to **4a** requires substantially less rearrangements of the crystal compared to **3a**. Thus, we can identify two key roles that drive the reaction in the solid phase: (1) the crystal environment can adopt the solvent role in enhancing the FLP reactivity, typically explained by both the electrostatic screening and undirectional London dispersion stabilization of the FLP products. (2) The static crystal field can selectively suppress certain undesired reaction routes, which is not possible in a liquid or gas environment with high molecular mobility.

**Fig. 5:**
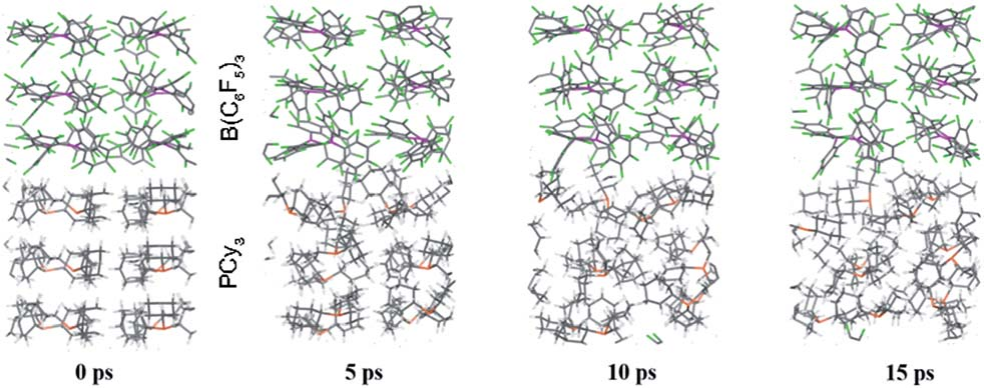
Snapshots of the periodic BO-MD simulation at the DFTB-D3 level of theory for the PCy_3_ + B(C_6_F_5_)_3_ FLP (**1a**/**2**). Color legend: P yellow, B pink, C black, F green and H white.

While point (1) makes the heterogeneous formulation of typical FLP reactions possible, (2) goes beyond it and opens possibilities for new FLP systems as compellingly demonstrated for the here discussed compound. An additional important prerequisite for the discussed reaction is the possible diffusion of H_2_ gas through the reactant crystal. Our molecular dynamics (MD) simulation (for details see the ESI†) confirm that H_2_ can actually move more or less freely through the channels of the crystal thereby generating the correct conditions for the H_2_ activation to take place. Moreover, we have conducted MD simulations for a model of the interface between the Lewis acid and base as it may occur experimentally in a mixture of the solid particles. According to these results which are shown in Fig. 5, at the interface the components (in particular the Lewis base) are spatially not constrained, possibly due to a mismatch of the molecular surfaces (a kind of interface strain). This leads partially to a “liquid phase” behavior of the FLPs in the solid state. It is seen that the Lewis acid and base components could move rather freely at the contact surface and adopt molecular FLP conformations enabling hydrogen activation as in solution (for details see the ESI†).

The new solid state phosphane/borane FLP reactions are not limited to the splitting of dihydrogen. We exposed the 1 : 1 mixture of PCy_3_ (**1a**) and B(C_6_F_5_)_3_ (**2**) for 4 hours at r.t. in the solid state to SO_2_ gas (1.5 bar).^47^ A sample was dissolved in CD_2_Cl_2_ and its NMR spectra revealed the almost quantitative formation of the P/B FLP SO_2_ addition product **5**. [Scheme 1b (SO_2_)] the product was also directly identified from the solid obtained by solid state NMR spectroscopy (see the ESI†), indicating essentially quantitative conversion. The distorted four-coordinate boron environment in **5** is characterized by *δ*_iso_ = –0.6 ppm, and a nearly axially symmetric electric field gradient, with *C*_Q_ = 1.54 MHz and *η*_Q_ = 0.15. The ^31^P MAS-NMR spectrum shows a single sharp signal at 51.5 ppm. In this case, ^11^B{^31^P} REDOR and ^31^P{^11^B} REAPDOR experiments consistently point towards a B···P internuclear distance of 450 pm, which is in good agreement with the distance of 434 pm from the crystal structure.

We performed the reaction on a preparative scale and isolated the product **5** as a white solid after recrystallization from CH_2_Cl_2_/pentane in 84% yield. The product shows the typical ^11^B (*δ* –0.3) and ^19^F NMR features (three resonances, Δ*δ*^19^F_*m*,*p*_ = 6.4 ppm) of the borate section of the molecule and a phosphonium ^31^P NMR signal at *δ* 50.0 (for further details see the ESI†).

**Fig. 6:**
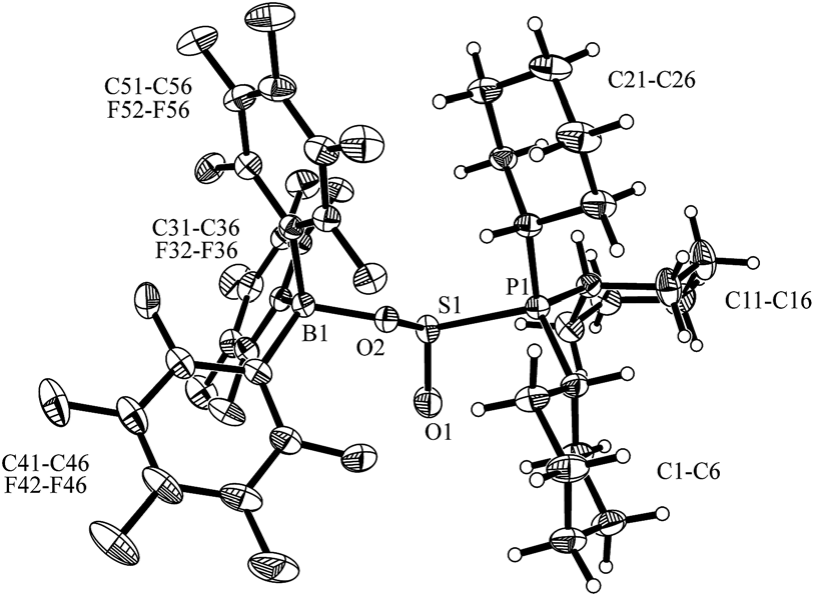
Molecular structure of the solid state P/B FLP SO_2_ addition product **5**. Selected bond lengths (Å): S1–O1 1.445(2), S1–O2 1.564(2), P1–S1 2.268(1), B1–O2 1.545(3) and bond angles (°): O1–S1–O2 110.8(1), O1–S1–P1 106.3(1), O2–S1–P1 93.4(1), ΣB1^CCC^ 334.0, ΣP1^CCC^ 334.1.

Compound **5** was characterized by X-ray diffraction (Fig. 6). The X-ray crystal structure analysis shows the newly formed P1–S1 and O2–B1 bonds. Both the phosphorus and the boron atoms show pseudo-tetrahedral coordination geometries. The sulfur coordination geometry is distorted trigonal-pyramidal. We also exposed the PCy_3_/B(C_6_F_5_)_3_ pair to SO_2_ in solution but only observed the formation of the substitution product **3a** [Scheme 1a (SO_2_); see the ESI† for details].

Our study has shown so far that an agitated mixture of particles of the phosphanes **1a–c** with particles of the B(C_6_F_5_)_3_ Lewis acid **2** did very effectively evade the deactivating S_N_Ar reaction that these pairs rapidly undergo in solution. Instead, they retained their frustrated Lewis pair character and, consequently, showed the ability to split dihydrogen heterolytically. While this observation is probably of a far-reaching principal interest, the rather harsh conditions of the solid state FLP H_2_-splitting (50 bars of dihydrogen, 3 to 10 d reaction time) made this far from a conveniently applicable procedure.

Fluorous liquids show some extraordinary properties.^48^–^55^ They do not mix with a variety of common organic solvents; they show an enhanced solubility of many gases in them, among them dihydrogen.^48^,^55^–^57^ Moreover, many organic and element-organic compounds, among them the phosphanes **1a–c** and B(C_6_F_5_)_3_ (**2**) are insoluble in them. Therefore, we decided to carry out the solid state FLP dihydrogen splitting reaction in perfluoromethylcyclohexane (F_11_C_6_–CF_3_). In a typical experiment (see the ESI† for details) we suspended an equimolar mixture of PCy_3_ (**1a**) and B(C_6_F_5_)_3_ in perfluoromethylcyclohexane and stirred the suspension for 10 h in a dihydrogen atmosphere at near to ambient conditions (r.t., 1.5 bar H_2_). Workup was simply done by evaporation of the volatiles. A sample of the obtained white powdery solid was then subjected to NMR analysis in d_2_-dichloromethane solution. It showed that a *ca.* 60% conversion to the hydrogen splitting product HPCy_3_^+^/HB(C_6_F_5_)_3_^–^ (**4a**) had been achieved. The remaining starting material had become converted to the S_N_Ar reaction product **3a** under the conditions of the NMR analysis in solution. The solid state NMR spectra of the products obtained after the suspension reaction showed the formation of **4a**.

The reaction of the PPhCy_2_ (**1b**)/B(C_6_F_5_)_3_ pair with dihydrogen proceeded at least equally well in this fluorous liquid. Under analogous conditions a *ca.* 95% conversion to the H_2_-splitting product **4b** was achieved within the 10 h reaction time. The PPh_2_^*t*^Bu (**1c**)/B(C_6_F_5_)_3_ system even slightly surpassed this result. We obtained a near to quantitative conversion to the HPPh_2_^*t*^Bu^+^/HB(C_6_F_5_)_3_^–^ salt within 10 h at near to ambient conditions in the inert perfluoromethylcyclohexane liquid (Table 1, see the ESI† for details).

**Table 1:** A Comparison of the dihydrogen splitting reaction by the phosphane/borane FLPs **1**/B(C_6_F_5_)_3_ in solution, in the dry solid state and in the fluorous liquid perfluoromethylcyclohexane

	No.	solution^*c*^	Dry solid	In F_11_C_6_–CF_3_^*b*^
PCy_3_	**1a**	Only **3a**	>90% conv. to **4a**^*a*^	60% conv. to **4a**
PPhCy_2_	**1b**	**3b** : **4b** ∼ 6 : 1	*ca.* 95% conv. to **4b**^*d*^	*ca.* 95% conv. to **4b**
PPh_2_^*t*^Bu	**1c**	**3c** : **4c** ∼ 2 : 1	*ca.* 95% conv. to **4c**^*d*^	*ca.* 98% conv. to **4c**

^*a*^10 days, r.t., 50 bar H_2_.

^*b*^10 hours, r.t., 1.5 bar H_2_.

^*c*^ d_2_-Dichloromethane, 12 hours, r.t., 50 bar H_2_.

^*d*^3 days, r.t., 50 bar H_2_.

## Conclusions

Our study has shown that frustrated Lewis pair behavior can be achieved by other means than the usual electronic^3^,^19^ or steric modifications^4^–^9^ of Lewis acids and bases. In the cases reported here we have deviated from the ubiquitous method of using sterically bulky substituents at the phosphorus Lewis base in order to prevent neutralizing adduct formation or other deactivating reactions with the strong B(C_6_F_5_)_3_ boron Lewis acid. On the contrary, the phosphanes used in this study are rather nucleophilic and in solution they undergo rapid nucleophilic aromatic substitution at one C_6_F_5_ ring of the Lewis acid replacing fluoride which consequently then becomes bonded to the boron atom^31^ with annihilation of its Lewis acidic features. In all the cases looked at in our study this FLP deactivating reaction is very efficiently suppressed by localizing the individual Lewis acidic and Lewis basic molecules inside their solid state lattices. This prevents them effectively from undergoing the bimolecular deactivation reaction.

From the solid state NMR and the DFT analysis in conjunction with general principles about molecular diffusion in the solid state, we assume that the individual Lewis acid and base components do not easily mix on a molecular level in our experiments, but that initially we are dealing with separate solid state phosphane and borane particles. This lets us assume that the FLP dihydrogen splitting reaction must take place at the surface, respectively the interface between phosphane and borane solid state entities; the ensuing reaction is, however, probably facilitated by the easy permeability of the respective crystal lattices by dihydrogen (and other gases). The dihydrogen splitting reaction may then have formed the phosphonium/hydridoborate salt initially at the surface, but it may be assumed that accumulation of that species creates a local situation resembling an ionic liquid, which might facilitate diffusion and mixing since eventually we obtained homogeneous solid samples of the respective dihydrogen splitting products. The MD simulations which support this view are currently due to the large system size too approximate (short) to draw any quantitative conclusions nor are we able to simulate further parts of the solid state reaction dynamically. Nevertheless, the MD and static theoretical results clearly support the above described picture of partially “molten” material at the interface with sufficient molecular flexibility for activation of almost freely diffusing molecular hydrogen.

Although the mechanistic aspects of our solid state FLP reactions must remain somewhat speculative at this time, we have greatly improved its practical applicability by using the fluorous liquid effect.^55^–^57^ This has made dihydrogen splitting reactions readily available from frustrated Lewis pair combinations which cannot be kept active in other ways.^33^ We have further found first indications that the resulting [P]H^+^/[B]H^–^ product **4a** can be usefully employed in imine reduction and we have shown that the solid state FLP reactions are not confined to the dihydrogen splitting reactions but can be developed beyond. The solid state approach can make FLPs available for small molecule activation beyond using the conventional methods leading to Lewis pair “frustration”. It needs to be explored if this will open new pathways of extending FLP chemistry beyond its existing scope, for example by opening FLP routes to the large field of heterogeneous catalysis, here to be performed without the aid of metals.^27^–^30^

## Conflicts of interest

There are no conflicts to declare.

## Supplementary Material

Supplementary information

Crystal structure data
