# Supplementary material for: Solid state frustrated Lewis pair chemistry
Source: Chem Sci. 2018 Apr 23;9(21):4859–65. doi: 10.1039/c8sc01089g (PMC5982199; doi:10.1039/c8sc01089g)
Supplement: Supplementary file 1 [file SC-009-C8SC01089G-s001.pdf]

Electronic Supplementary Material (ESI) for Chemical Science.
This journal is © The Royal Society of Chemistry 2018

Supplementary Information for

Solid State frustrated Lewis pair chemistry

Long Wang, Gerald Kehr, Constantin G. Daniliuc, Melanie Brinkkötter,
Thomas Wiegand, Anna-Lena Wübker, Hellmut Eckert*, Lei Liu, Jan Gerit Brandenburg,
Stefan Grimme*, Gerhard Erker*

correspondence to: erker@uni-muenster.de, eckerth@uni-muenster.de,
grimme@thch.uni-bonn.de

This PDF file includes:

Materials and Methods
SupplementaryText
Figures. S1 to S88
Tables S1 to S5
Scheme S1 to S13

Supplementary Information

(Part 1. Experimental and Analytical Details) S3
Materials and Methods S3
1) Reactions of PCy_3 (**1a**) with $B(C_6F_5)_3$ S4
1.1) Reaction of PCy_3 (**1a**) with $B(C_6F_5)_3$ in a H_2 atmosphere in the solid state: preparation of compound **4a** and control mixture sample S4
1.2) Reaction of PCy_3 (**1a**) with $B(C_6F_5)_3$ in a H_2 atmosphere in the solid state: isolation of compound **4a** S4
1.3) Reaction of PCy_3 (**1a**) with $B(C_6F_5)_3$ in solution: preparation of compound **3a** S8
1.4) Reaction of PCy_3 (**1a**) with $B(C_6F_5)_3$ in solution in a H_2 atmosphere: generation of compound **3a** S12
2) Reactions of $PhPCy_2$ (**1b**) with $B(C_6F_5)_3$ S15
2.1) Reaction of $PhPCy_2$ (**1b**) with $B(C_6F_5)_3$ in a H_2 atmosphere in the solid state: preparation of compound **4b** S15
2.2) Reaction of $PhPCy_2$ (**1b**) with $B(C_6F_5)_3$ in solution: generation of compound **3b** S19
2.3) Reaction of $PhPCy_2$ (**1b**) with $B(C_6F_5)_3$ in solution in a H_2 atmosphere: generation of a mixture of compounds **3b** and **4b** S21
3) Reactions of Ph_2P^tBu (**1c**) with $B(C_6F_5)_3$ S24
3.1) Reaction of Ph_2P^tBu (**1c**) with $B(C_6F_5)_3$ in a H_2 atmosphere in the solid state: preparation of compound **4c** S24
3.2) Reaction of Ph_2P^tBu (**1c**) with $B(C_6F_5)_3$ in solution: generation of compound **3c** S28
3.3) Reaction of Ph_2P^tBu (**1c**) with $B(C_6F_5)_3$ in solution in a H_2 atmosphere: generation of a mixture of compounds **3c** and **4c** S30
4) Reactions of PCy_3 (**1a**) and $B(C_6F_5)_3$ with SO_2 S33
4.1) Reaction of PCy_3 (**1a**) and $B(C_6F_5)_3$ with SO_2 in the solid state: preparation of compound **5** S33
4.2) Reaction of PCy_3 (**1a**) and $B(C_6F_5)_3$ with SO_2 in the solid state: isolation of compound **5** S34
4.3) Reaction of PCy_3 (**1a**) and $B(C_6F_5)_3$ with SO_2 in solution: generation of compound **3a** S38
5) Reactions in perfluoromethylcyclohexane solvent S40
5.1) Reactions of PCy_3 (**1a**) with $B(C_6F_5)_3$ in perfluoromethylcyclohexane in a H_2 atmosphere and control experiment S40
5.2) Reactions of $PhPCy_2$ (**1b**) with $B(C_6F_5)_3$ in perfluoromethylcyclohexane in a H_2 atmosphere S42
5.3) Reactions of PCy_3 (**1c**) with $B(C_6F_5)_3$ in perfluoromethylcyclohexane in a H_2 atmosphere S45
6) Reduction of an imine by compound **4a** S47
Supplementary Information (Part 2. Solid State NMR) S49
Supplementary Information (Part 3. Computational Section) S58
References S63

Supplementary Information (Part 1. Experimental and Analytical Details)

Long Wang[a], Gerald Kehr[a], Constantin G. Daniliuc[a], Gerhard Erker[a,*]

[a] Organisch-Chemisches Institut, Westfälische Wilhelms-Universität Münster, Corrensstraße 40, 48149 Münster, Germany

Materials and Methods

All experiments were carried out in a dry argon atmosphere using an MBraun glove box and/or standard Schlenk techniques. All solvents were dried and stored under an argon atmosphere before use. NMR spectra were measured on a Varian INOVA 500 MHz spectrometer (^{1}H 500 MHz; ^{13}C, 126 Hz; ^{11}B, 160 MHz; ^{19}F, 470 Hz) or a Varian UNITY plus 600 MHz spectrometer (^{1}H 600 MHz; ^{13}C, 151 Hz; ^{11}B, 192 MHz; ^{19}F, 564 Hz). Chemical shifts are given relative to $SiMe_4$ and referenced to the respective residual solvent signal (^{1}H NMR and ^{13}C NMR) or an external standard [δ ($BF_3{\cdot}OEt_2$) = 0 for ^{11}B NMR, δ ($CFCl_3$) = 0 for ^{19}F NMR]. NMR assignments were supported by additional 1D and 2D NMR experiments.

Compounds **1a**, **1b**, **1c** were bought from Sigma-Aldrich and used without further purification (stored in glove box to avoid moisture and air). Compound **2** was purchased from Boulder Scientific, purified by recrystallization in pentane and then sublimation before use. SO_2 gas was commercially available and further dried by passing through a column of P_4O_{10} . Fluorous solvent perfluoro(methylcyclohexane) was bought from Sigma-Aldrich (technical grade, 90%) and used without further purification (stored in glove box to avoid moisture and air). Unless otherwise noted, all chemicals were purchased from commercially available sources.

X-Ray diffraction: For compound **5a** data sets were collected with a Kappa CCD APEXII Bruker diffractometer. For compounds **4a** and **3b** data sets were collected with a D8 Venture Dual Source 100 CMOS diffractometer. Programs used: data collection: APEX2 V2014.5-0 (Bruker AXS Inc., 2014); cell refinement: SAINT V8.34A (Bruker AXS Inc., 2013); data reduction: SAINT V8.34A (Bruker AXS Inc., 2013); absorption correction, SADABS V2014/2 (Bruker AXS Inc., 2014); structure solution SHELXT-2014 (Sheldrick, 2014); structure refinement SHELXL-2014 (Sheldrick, 2014) and graphics, XP (Bruker AXS Inc., 2014). *R*-values are given for observed reflections, and wR^2 values are given for all reflections. *Exceptions and special features*: For compound **4a** one additional dichloromethane molecule disordered over two positions was found in the asymmetric unit. Several restraints (SADI, SAME, ISOR and SIMU) were used in order to improve refinement stability of the compound. For compound **5a** one half badly disordered hexane molecule *was found in the asymmetrical unit and could not be satisfactorily refined. The program SQUEEZE* (A. L. Spek J. Appl. Cryst., 2003, 36, 7-13) was therefore used to remove mathematically the effect of the solvent. The quoted formula and derived parameters do not include the squeezed solvent molecule.

1) Reactions of PCy_3 (**1a**) with $B(C_6F_5)_3$

1.1) Reaction of PCy_3 (**1a**) with $B(C_6F_5)_3$ in a H_2 atmosphere in the solid state: preparation of compound **4a** and control mixture sample

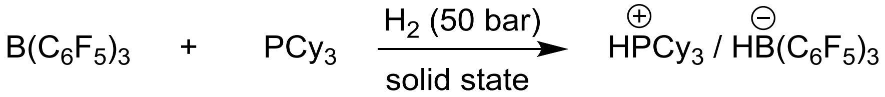

1a **4a**

Scheme S1. Reaction of PCy_3 (**1a**) with $B(C_6F_5)_3$ in the solid state: preparation of compound **4a**

A mixture of $B(C_6F_5)_3$ (102.4 mg, 0.2 mmol) and PCy_3 (56.1 mg, 0.2 mmol) was put in a vial with a double valve adaptor[a] and stirred for 15 min. Then the reaction mixture was stirred in a dihydrogen atmosphere (50 bar) for 10 days. After the reaction time the obtained powder was characterized by NMR experiments in solution (>90% conversion) and in the solid state.[1]

The obtained NMR data of the obtained powder in solution (dichloromethane-d_2) were consistent to those given for the isolated compound **4a** (see below).

*****Control mixture sample: A mixture of $B(C_6F_5)_3$ (102.4 mg, 0.2 mmol) and PCy_3 (56.1 mg, 0.2 mmol) was put in a vial with a double valve adaptor and stirred for 10 days in a argon atmosphere. The obtained control mixture sample was characterized by NMR experiments in the solid state.

1.2) Reaction of PCy_3 (**1a**) with $B(C_6F_5)_3$ in a H_2 atmosphere in the solid state: isolation of compound **4a**

A mixture of $B(C_6F_5)_3$ (102.4 mg, 0.2 mmol) and PCy_3 (56.1 mg, 0.2 mmol) were put in a vial with a double valve adaptor[a] and stirred for 15 min. Then the reaction mixture was stirred in a dihydrogen atmosphere (50 bar) for 10 days. After the reaction time the obtained powder was crystallized from dichloromethane/pentane and dried in vacuo to give a white solid (128.3 mg, 0.162 mmol, 81 %).[1]

[Cy: cyclohexyl]

^{1}H NMR (500 MHz, 299K, dichloromethane-d_2) δ = 5.15 (dq, $^1J_{PH}$ = 444.0 Hz, $^3J_{HH}$ = 4.1 Hz, 1H, PH), 3.59 (br 1:1:1:1 q, $^1J_{BH}$ ~ 92 Hz, 1H, BH), 2.43 (m, 3H, CH^{Cy}), 1.96/1.55, 1.93/1.40 (each m, each 6H, CH_2^{Cy}), 1.82/1.31 (each m, each 3H, CH_2^{Cy}).

^{13}C{^{1}H} NMR (126 MHz, 299K, dichloromethane-d_2) δ = 148.6 (dm, $^1J_{FC}$ ~ 235 Hz, C_6F_5), 138.1 (dm, $^1J_{FC}$ ~ 240 Hz, C_6F_5), 136.8 (dm, $^1J_{FC}$ ~ 245 Hz, C_6F_5), 125.5 (br, i-C_6F_5), 28.5 (d, $^1J_{PC}$ = 38.3 Hz, CH^{Cy}), 28.4 (d, J_{PC} = 3.9 Hz), 26.4 (d, J_{PC} = 12.7 Hz), 25.2 (d, J_{PC} = 1.6 Hz)(CH_2^{Cy}).

^{11}B NMR (160 MHz, 299K, dichloromethane-d_2) δ = -25.3 (d, $^1J_{BH}$ ~ 92 Hz).

^{11}B{^{1}H} NMR (160 MHz, 299K, dichloromethane-d_2) δ = -25.3 ($\nu_{1/2}$ ~ 40 Hz).

^{19}F NMR (470 MHz, 299K, dichloromethane-d_2) δ = -133.9 (m, 2F, *o*-C_6F_5), -164.6 (t, $^3J_{FF}$ = 20.2 Hz, 1F, *p*-C_6F_5), -167.5 (m, 2F, *m*-C_6F_5), [$\Delta\delta^{19}$Fm,p = 2.9].

^{31}P NMR (202 MHz, 299K, dichloromethane-d_2) δ = 33.2 (br d, $^1J_{PH}$ ~ 443 Hz).

^{31}P{^{1}H} NMR (202 MHz, 299K, dichloromethane-d_2) δ = 33.2 ($\nu_{1/2}$ ~ 3 Hz).

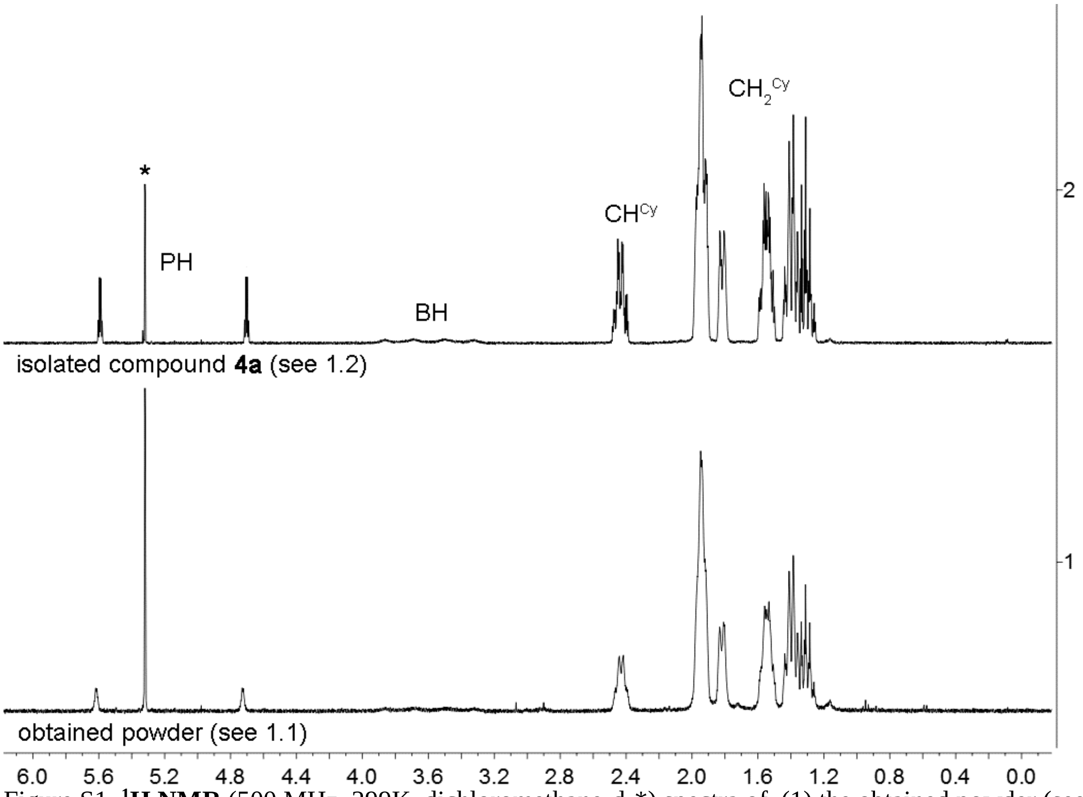

Figure S1. **^{1}H NMR** (500 MHz, 299K, dichloromethane-d$_2$*) spectra of (1) the obtained powder (see experiment 1.1) and (2) the isolated compound **4a** (see experiment 1.2).

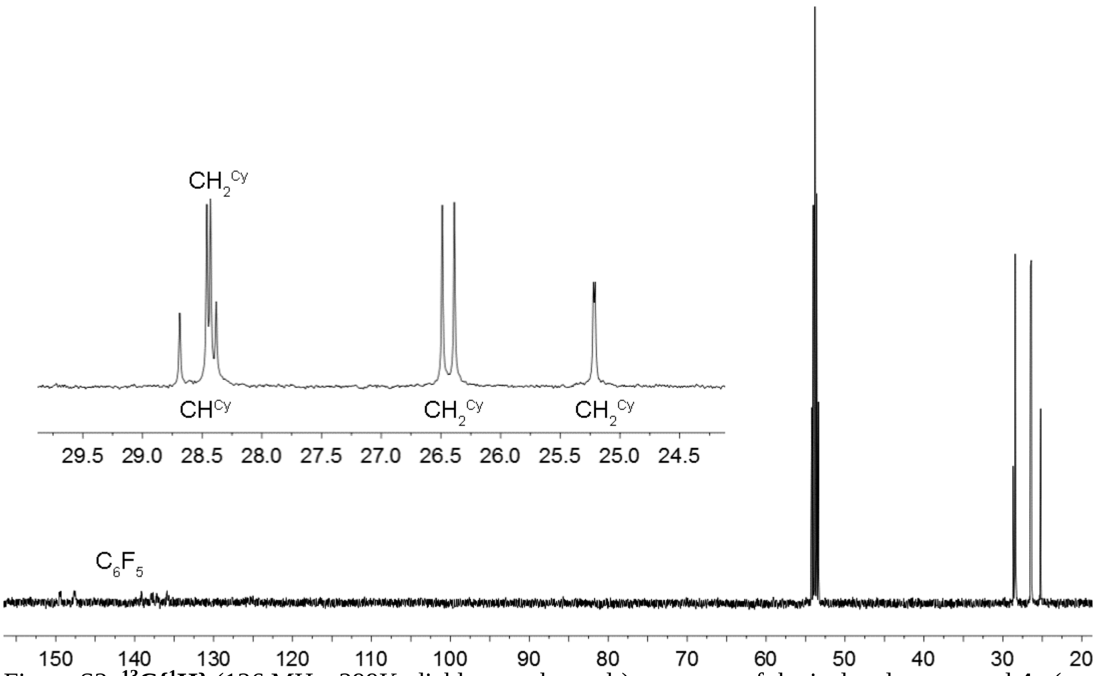

Figure S2. **^{13}C{^{1}H}** (126 MHz, 299K, dichloromethane-d$_2$) spectrum of the isolated compound **4a** (see experiment 1.2).

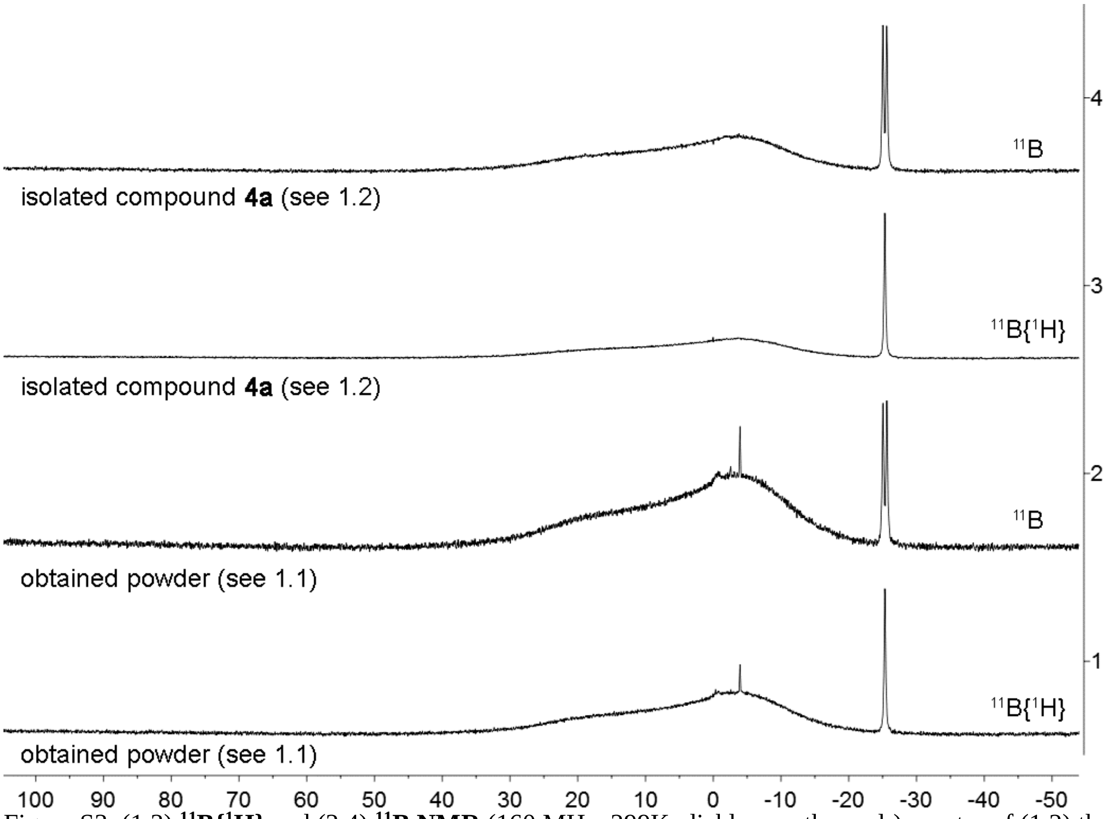

Figure S3. (1,3) **^{11}B{^{1}H}** and (2,4) **^{11}B NMR** (160 MHz, 299K, dichloromethane-d$_2$) spectra of (1,2) the obtained powder (see experiment 1.1) and (3,4) the isolated compound **4a** (see experiment 1.2).

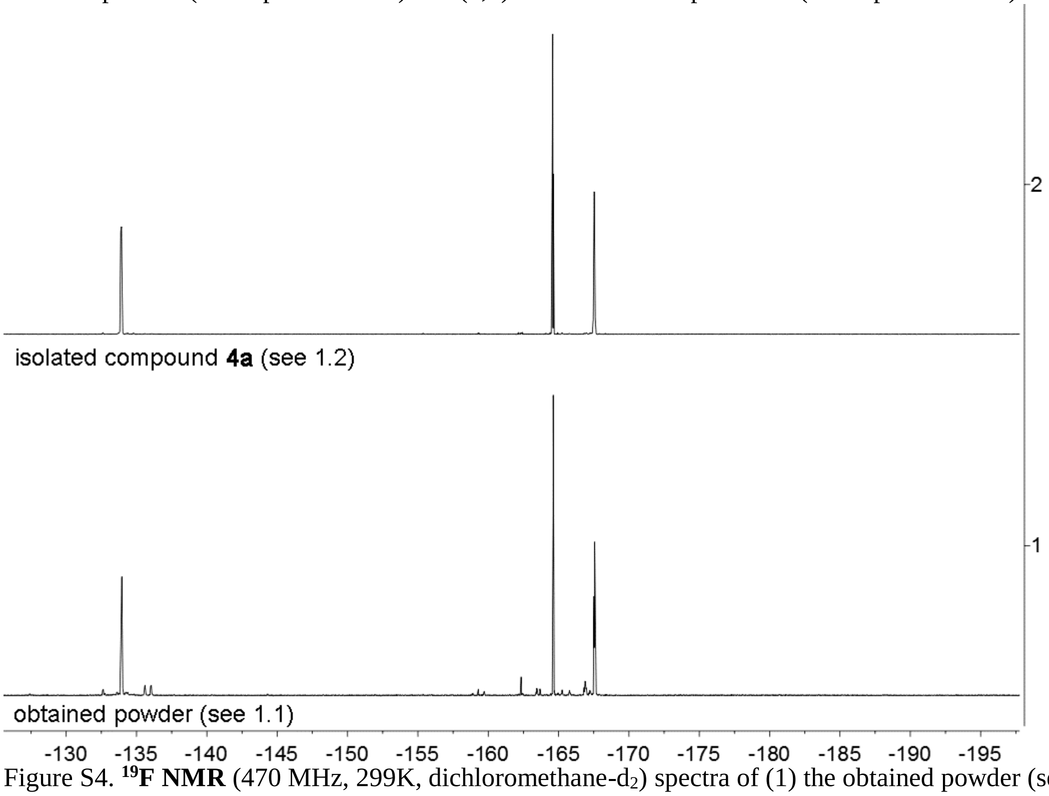

Figure S4. **^{19}F NMR** (470 MHz, 299K, dichloromethane-d$_2$) spectra of (1) the obtained powder (see experiment 1.1) and (2) the isolated compound **4a** (see experiment 1.2).

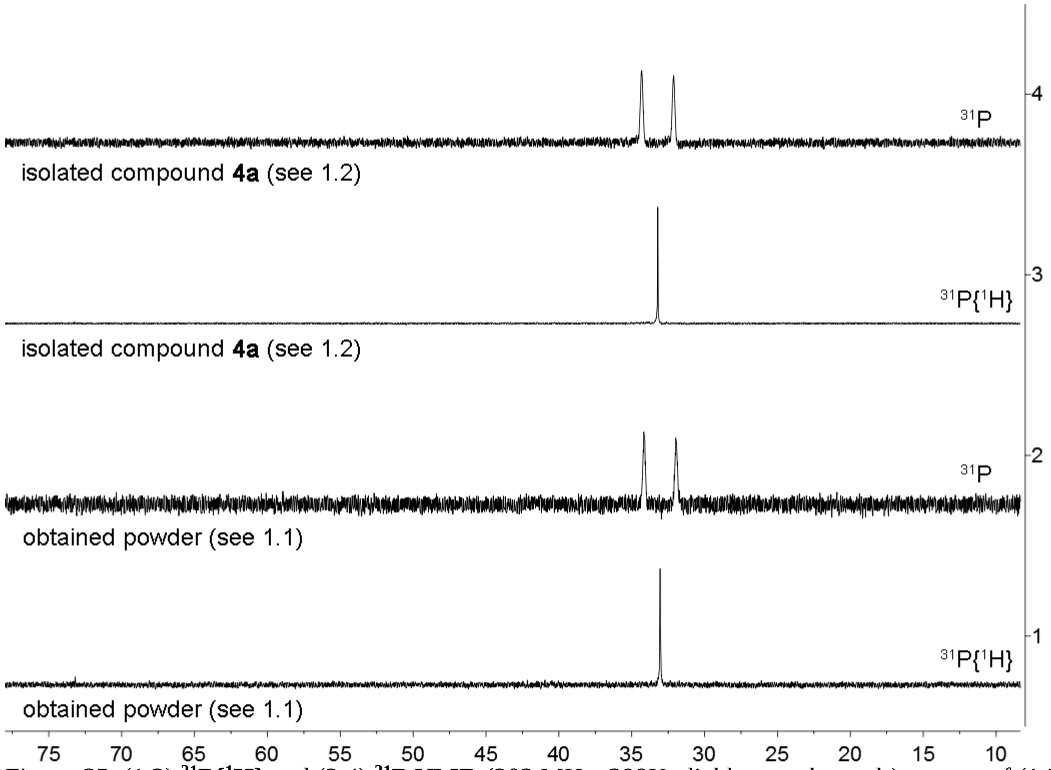

Figure S5. (1,3) **^{31}P{^{1}H}** and (2,4) **^{31}P NMR** (202 MHz, 299K, dichloromethane-d_2) spectra of (1,2) the obtained powder (see experiment 1.1) and (3,4) the isolated compound **4a** (see experiment 1.2).

Single crystals of compound **4a** suitable for the X-ray crystal structure analysis were obtained of diffusion of pentane to a solution of compound **3a** in dichloromethane at -36 ^{o}C. [X-ray crystal structure analysis see: Jiang, Y., Schirmer, B., Blacque, O., Fox, T., Grimme, S., and Berke, H. (2013). The “Catalytic Nitrosyl Effect”: NO Bending Boosting the Efficiency of Rhenium Based Alkene Hydrogenations. J. Am. Chem. Soc. *135*, 4088-4102.].[2]

X-ray crystal structure analysis of compound 4a: A colorless needle-like specimen of $C_{36}H_{35}BF_{15}P$, approximate dimensions 0.010 mm x 0.030 mm x 0.260 mm, was used for the X-ray crystallographic analysis. The X-ray intensity data were measured. A total of 2003 frames were collected. The total exposure time was 49.19 hours. The frames were integrated with the Bruker SAINT software package using a wide-frame algorithm. The integration of the data using a monoclinic unit cell yielded a total of 39592 reflections to a maximum θ angle of 65.07° (0.85 Å resolution), of which 5803 were independent (average redundancy 6.823, completeness = 99.7%, R_{int} = 15.69%, R_{sig} = 10.91%) and 3530 (60.83%) were greater than $2\sigma(F^2)$. The final cell constants of a = 28.898(3) Å, b = 11.2532(9) Å, c = 21.7565(19) Å, β = 105.318(5)°, volume = 6823.8(10) Å^3, are based upon the refinement of the XYZ-centroids of 4821 reflections above 20 σ(I) with 8.427° < 2θ < 131.8°. Data were corrected for absorption effects using the multi-scan method (SADABS). The ratio of minimum to maximum apparent transmission was 0.764. The calculated minimum and maximum transmission coefficients (based on crystal size) are 0.6640 and 0.9830. The final anisotropic full-matrix least-squares refinement on F^2 with 486 variables converged at R1 = 11.55%, for the observed data and wR2 = 33.37% for all

data. The goodness-of-fit was 1.019. The largest peak in the final difference electron density synthesis was 1.163 $e^-/Å^3$ and the largest hole was -0.506 $e^-/Å^3$ with an RMS deviation of 0.109 $e^-/Å^3$. On the basis of the final model, the calculated density was 1.547 g/cm^3 and F(000), 3248 e^-.

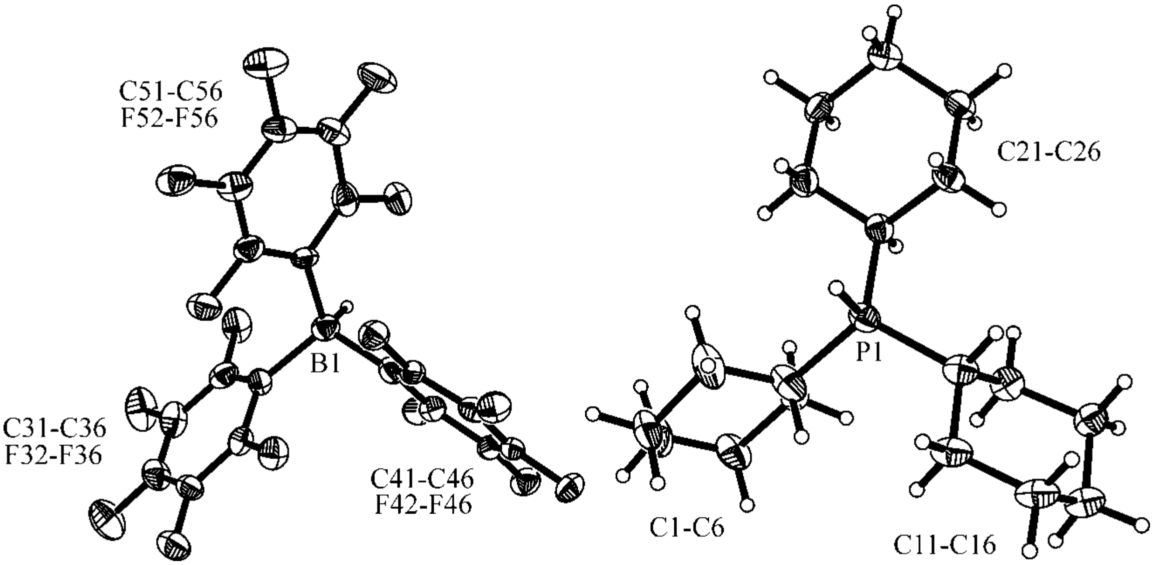

Figure S6. X-ray crystal structure analysis of compound 4a.

1.3) Reaction of PCy_3 (**1a**) with $B(C_6F_5)_3$ in solution: preparation of compound **3a**

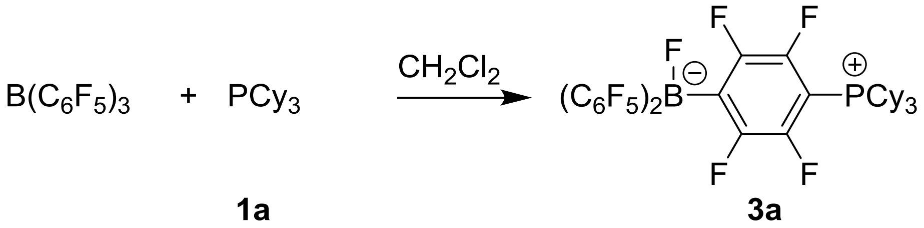

Scheme S2. Reaction of PCy_3 (**1a**) with $B(C_6F_5)_3$ in solution: preparation of compound **3a**.

A solution of PCy_3 (84.1 mg, 0.3 mmol) in CH_2Cl_2 (3 mL) was added to a solution of $B(C_6F_5)_3$ (153.6 mg, 0.3 mmol) in CH_2Cl_2 (3 mL). The mixture was stirred at room temperature for 12 hours. Then all volatiles were removed in vacuo to give a white solid, which was washed with pentane (1mL × 3) and dried in vacuo to finally give compound **3a** as a white solid (204.1 mg, 0.258 mmol, 86 %).[3] The white solid was also measured by solid state NMR.

^{1}H NMR (500 MHz, 299K, dichloromethane-d_2) δ = 2.92 (m, 1H, CH^{Cy}), 1.99/1.57, 1.96/1.41 (each br m, each 2H, CH_2^{Cy}), 1.84/1.34 (each br m, each 1H, CH_2^{Cy}).

$^{13}C\{^{1}H\}$ NMR (126 MHz, 299K, dichloromethane-d_2) δ = 33.1 (dt, $^{1}J_{PC}$ = 38.6 Hz, J = 4.2 Hz, CH^{Cy}), 27.9 (d, J_{PC} = 3.8 Hz), 27.3 (d, J_{PC} = 12.9 Hz), 25.7 (d, J_{PC} = 1.8 Hz)(CH_2^{Cy}), [C_6F_5 and C_6F_4 not listed].
$^{11}B\{^{1}H\}$ NMR (160 MHz, 299K, dichloromethane-d_2) δ = -0.6 (d, $^{1}J_{FB}$ ~ 67 Hz).
^{19}F NMR (470 MHz, 299K, dichloromethane-d_2) δ = -128.3, -131.7 (each m, each 2F, C_6F_4), - 135.5 (m, 4F, *o*), -161.6 (t, $^{3}J_{FF}$ = 20.0 Hz, 2F, *p*), -166.5 (m, 4F, *m*)(C_6F_5) [$\Delta\delta^{19}F_{m,p}$ = 4.9], 193.0 (br m, BF).
$^{31}P\{^{1}H\}$ NMR (202 MHz, 299K, dichloromethane-d_2) δ = 41.0 (m).

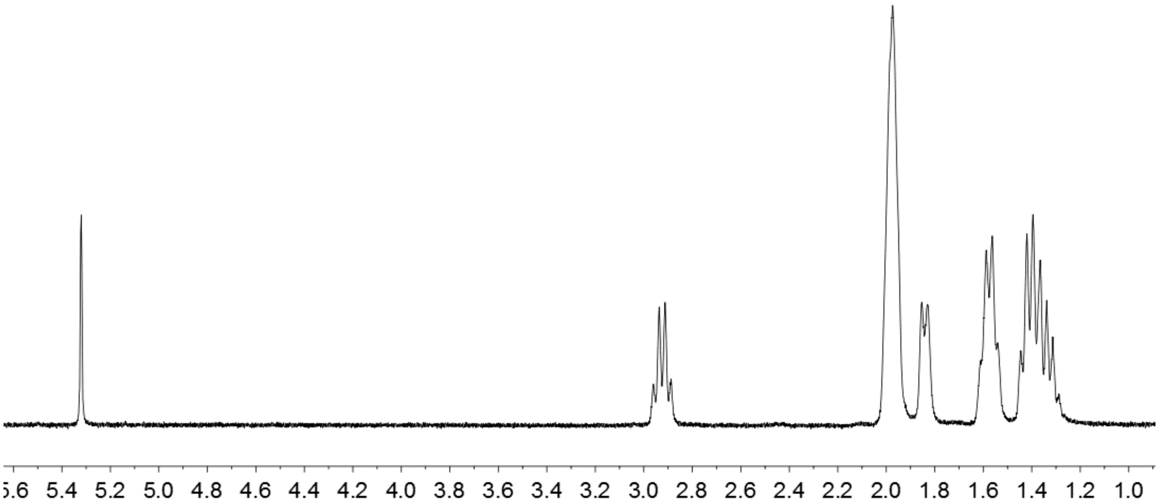
Figure S7. **^{1}H NMR** (500 MHz, 299K, dichloromethane-d_2) spectrum of compound **3a**.

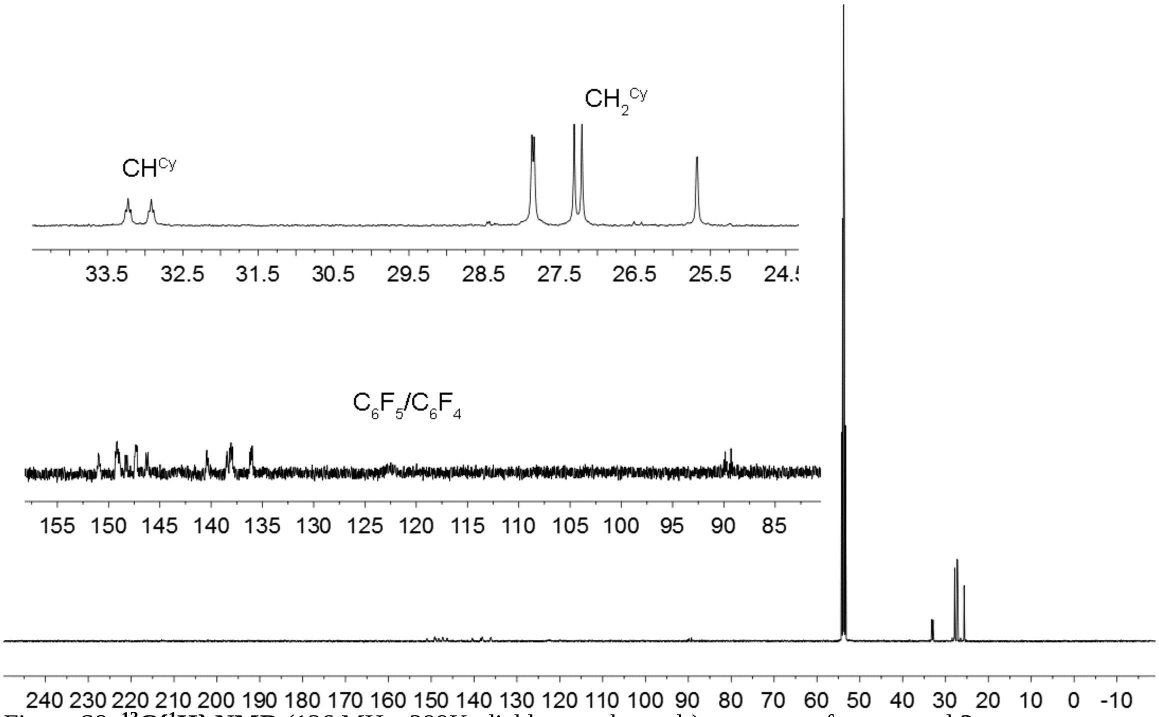

Figure S8. **$^{13}C\{^{1}H\}$ NMR** (126 MHz, 299K, dichloromethane-d_2) spectrum of compound **3a**

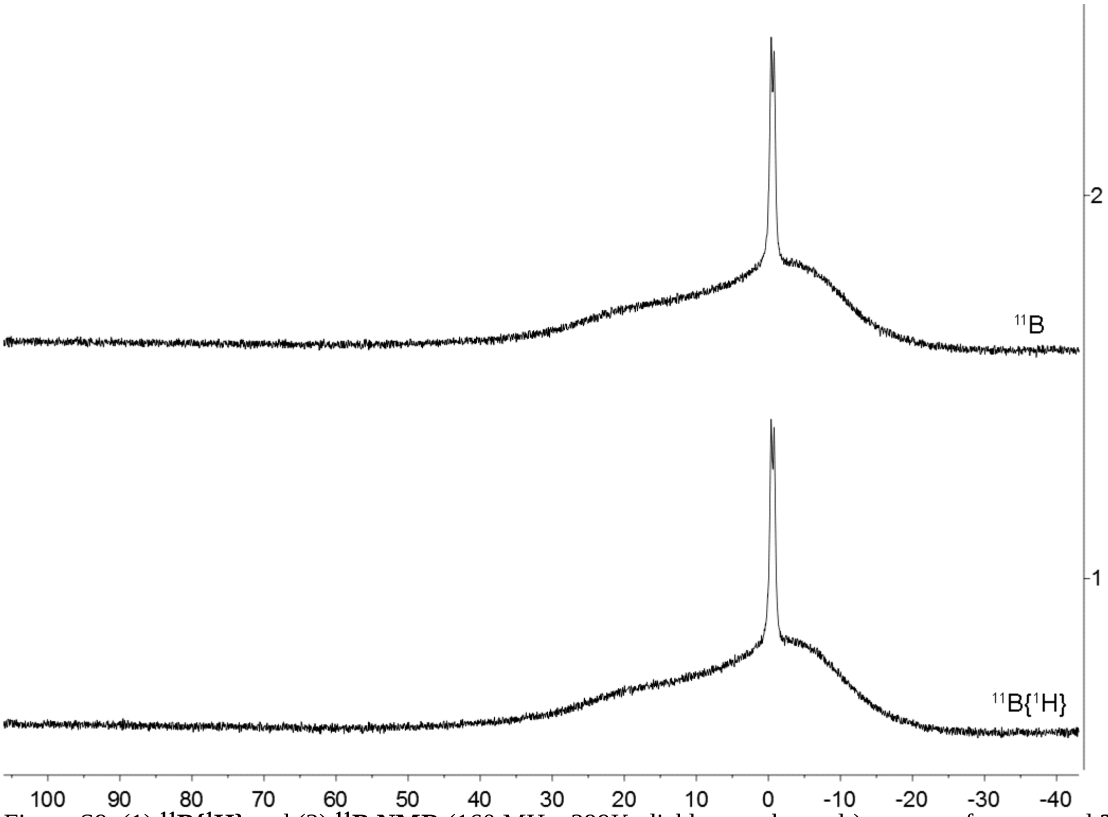

Figure S9. (1) **^{11}B{^{1}H}** and (2) **^{11}B NMR** (160 MHz, 299K, dichloromethane-d_2) spectra of compound **3a**.

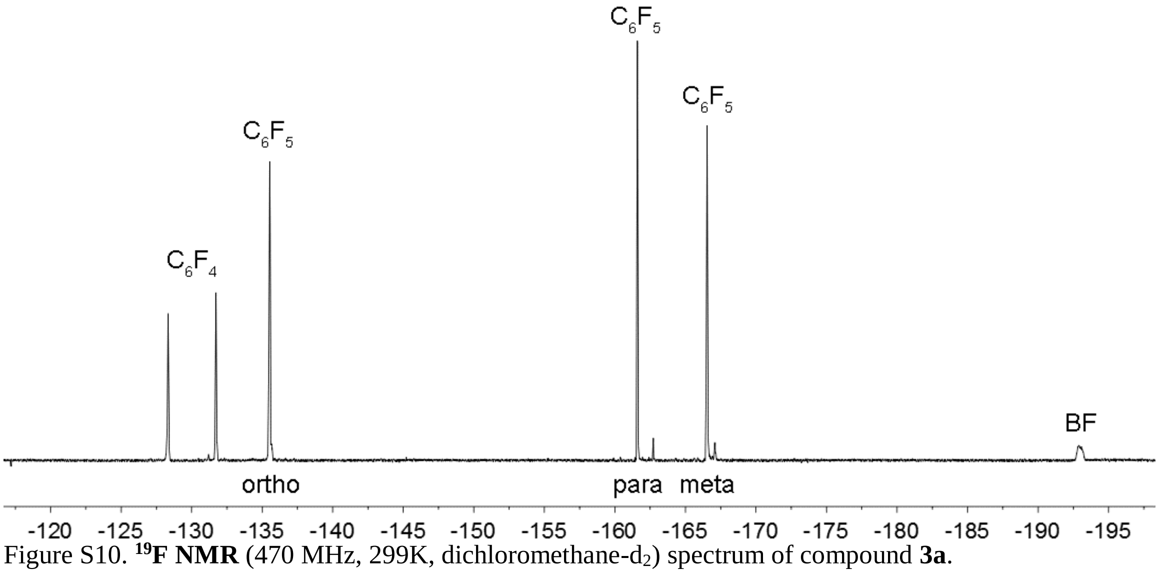

Figure S10. **^{19}F NMR** (470 MHz, 299K, dichloromethane-d_2) spectrum of compound **3a**.

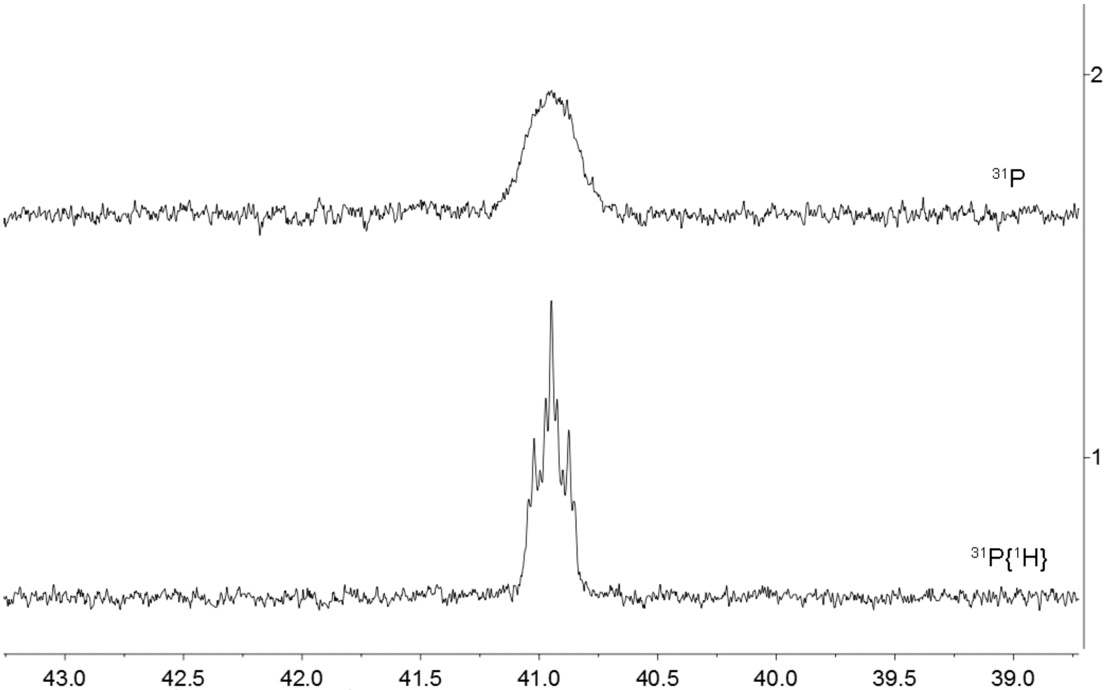

Figure S11. (1) **$^{31}P\{^1H\}$** and (2) **^{31}P NMR** (202 MHz, 299K, dichloromethane-d_2) spectra of compound **3a**.

Single crystals suitable for the X-ray crystal structure analysis were obtained by pentane diffusion to a solution of compound **3a** in dichloromethane at -36 ^oC.

X-ray crystal structure analysis of compound 3a: A colorless prism-like specimen of $C_{36}H_{33}BF_{15}P \cdot 2 \times CH_2Cl_2$ approximate dimensions 0.098 mm x 0.196 mm x 0.219 mm, was used for the X-ray crystallographic analysis. The X-ray intensity data were measured. A total of 984 frames were collected. The total exposure time was 16.40 hours. The frames were integrated with the Bruker SAINT software package using a narrow-frame algorithm. The integration of the data using an orthorhombic unit cell yielded a total of 194744 reflections to a maximum θ angle of 26.37° (0.80 Å resolution), of which 8145 were independent (average redundancy 23.910, completeness = 99.9%, R_{int} = 5.16%, R_{sig} = 1.41%) and 6970 (85.57%) were greater than $2\sigma(F^2)$. The final cell constants of a = 20.8497(8) Å, b = 16.0333(6) Å, c = 23.8954(9) Å, volume = 7988.0(5) $Å^3$, are based upon the refinement of the XYZ-centroids of 9344 reflections above 20 σ(I) with 4.962° < 2θ < 55.01°. Data were corrected for absorption effects using the multi-scan method (SADABS). The ratio of minimum to maximum apparent transmission was 0.948. The calculated minimum and maximum transmission coefficients (based on crystal size) are 0.9100 and 0.9580. The final anisotropic full-matrix least-squares refinement on F^2 with 560 variables converged at R1 = 4.05%, for the observed data and wR2 = 10.73% for all data. The goodness-of-fit was 1.047. The largest peak in the final difference electron density synthesis was 0.653 $e^-/Å^3$ and the largest hole was -0.742 $e^-/Å^3$ with an RMS deviation of 0.066 $e^-/Å^3$. On the basis of the final model, the calculated density was 1.600 g/cm^3 and F(000), 3904 e^-.

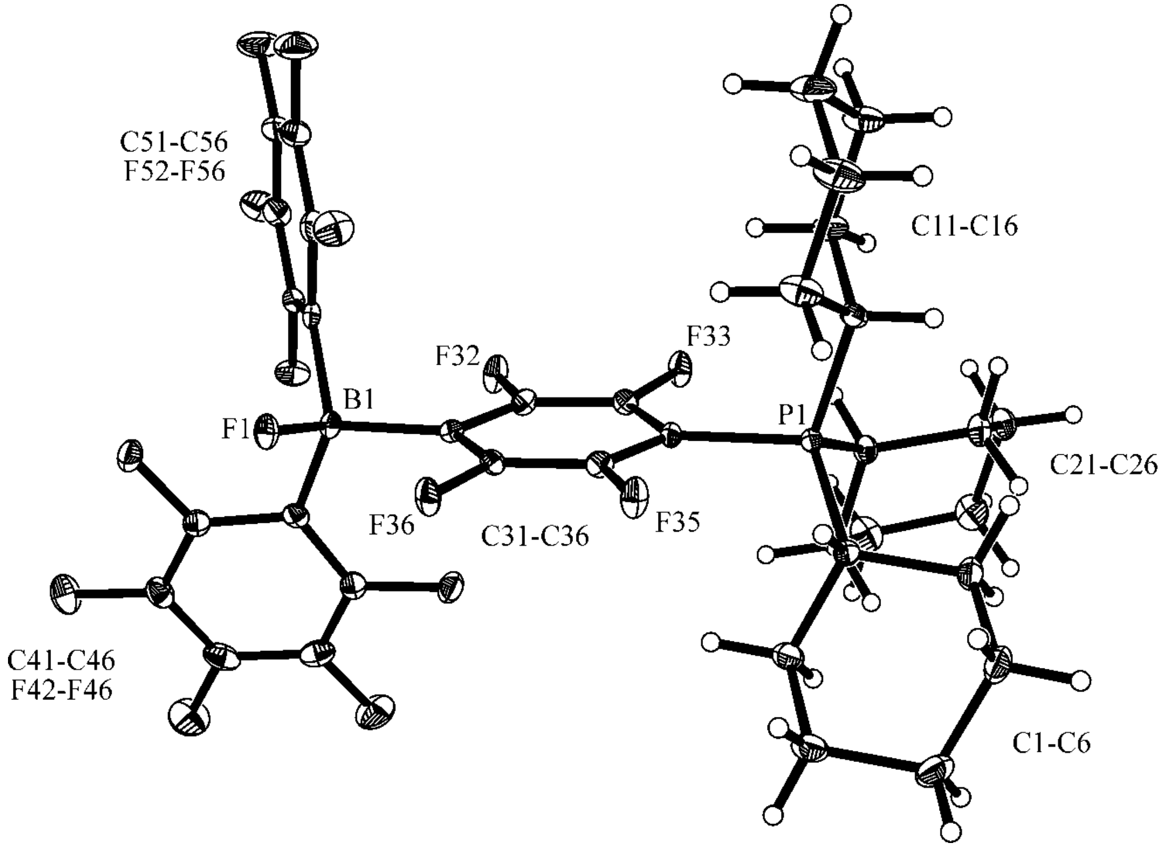

Figure S12. X-ray crystal structure analysis of compound **3a**.

1.4) Reaction of PCy_3 (**1a**) with $B(C_6F_5)_3$ in solution in a H_2 atmosphere: generation of compound **3a**

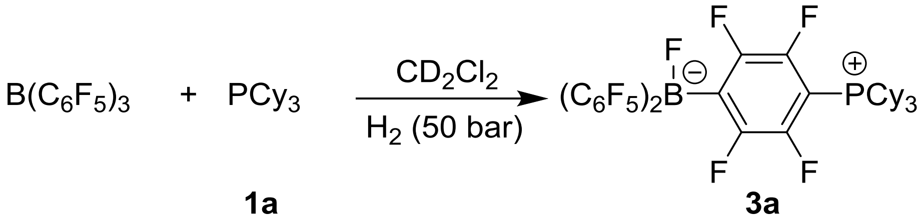

Scheme S3. Reaction of PCy_3 (**1a**) with $B(C_6F_5)_3$ in solution in a H_2 atmosphere: generation of compound **3a**

A solution of PCy_3 (14.0 mg, 0.05 mmol) and $B(C_6F_5)_3$ (25.6 mg, 0.05 mmol) in CD_2Cl_2 (1 mL) was put in a vial with a double valve adaptor[a] and stirred for 12 hours in an H_2 atmosphere (50 bar). Then the reaction solution was characterized by NMR experiments.[1]

[Comment: the obtained NMR data were consistent to those given for compound **3a** (see experiment 1.3)]

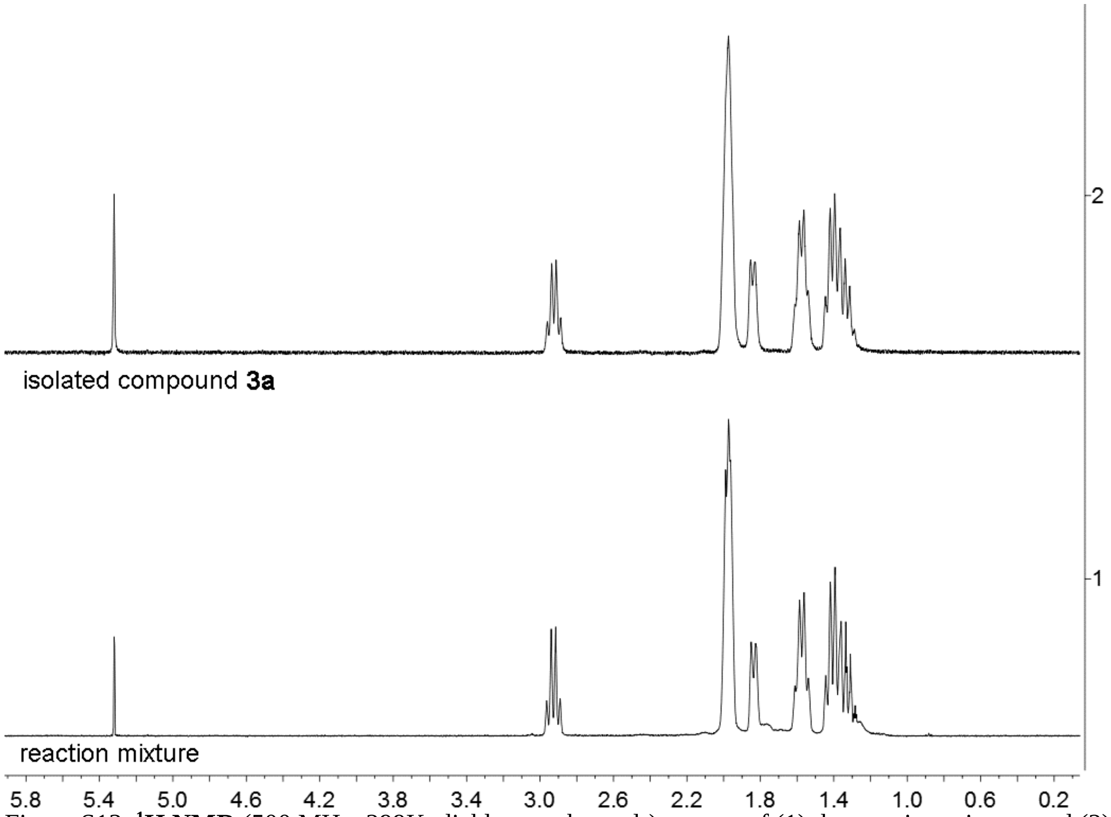

Figure S13. ^{1}**H NMR** (500 MHz, 299K, dichloromethane-d$_2$) spectra of (1) the reaction mixture and (2) the isolated compound **3a** (see experiment 1.3).

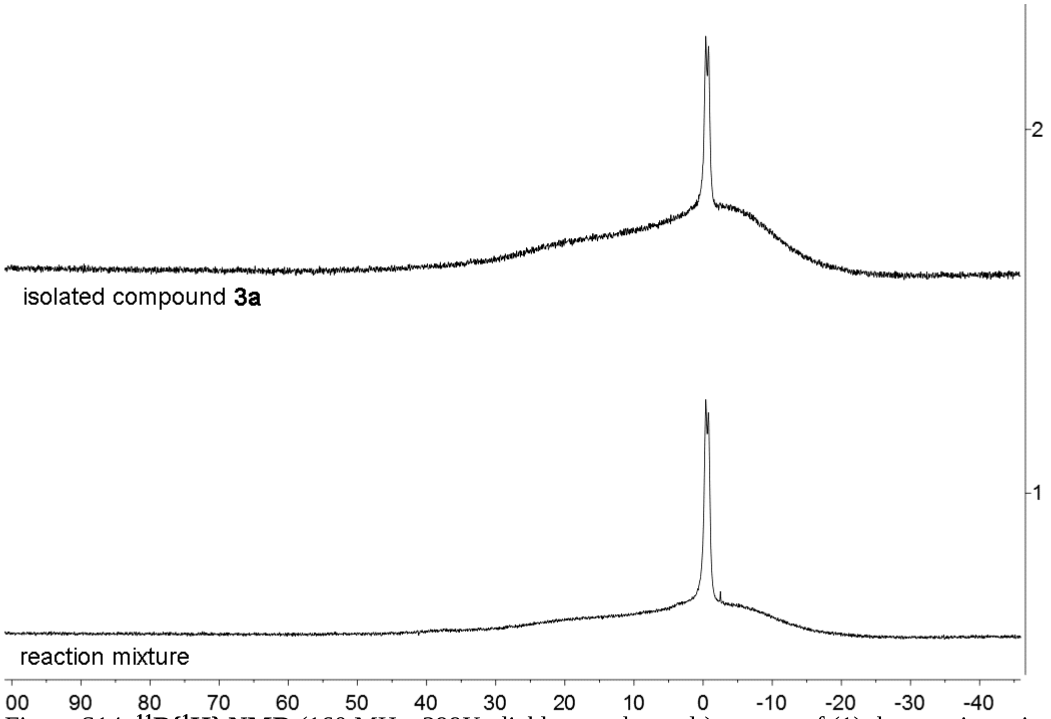

Figure S14. ^{11}**B{^{1}H} NMR** (160 MHz, 299K, dichloromethane-d$_2$) spectra of (1) the reaction mixture and (2) the isolated compound **3a** (see experiment 1.3).

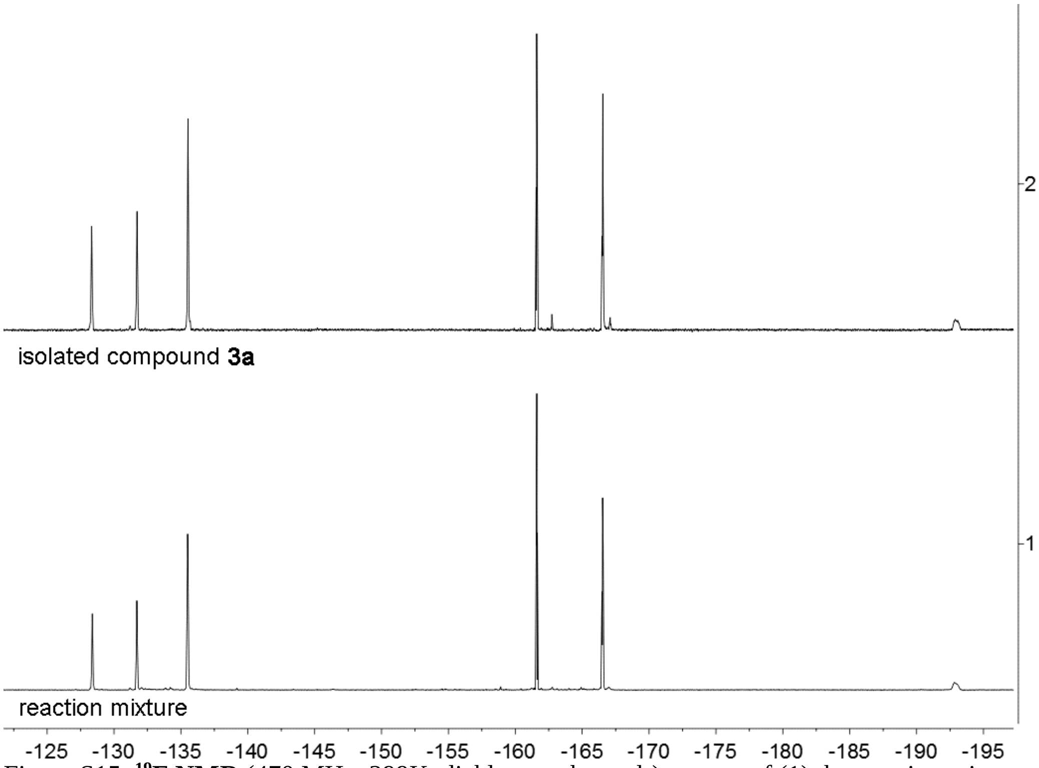

Figure S15. [19]**F NMR** (470 MHz, 299K, dichloromethane-d_2) spectra of (1) the reaction mixture and (2) the isolated compound **3a** (see experiment 1.3).

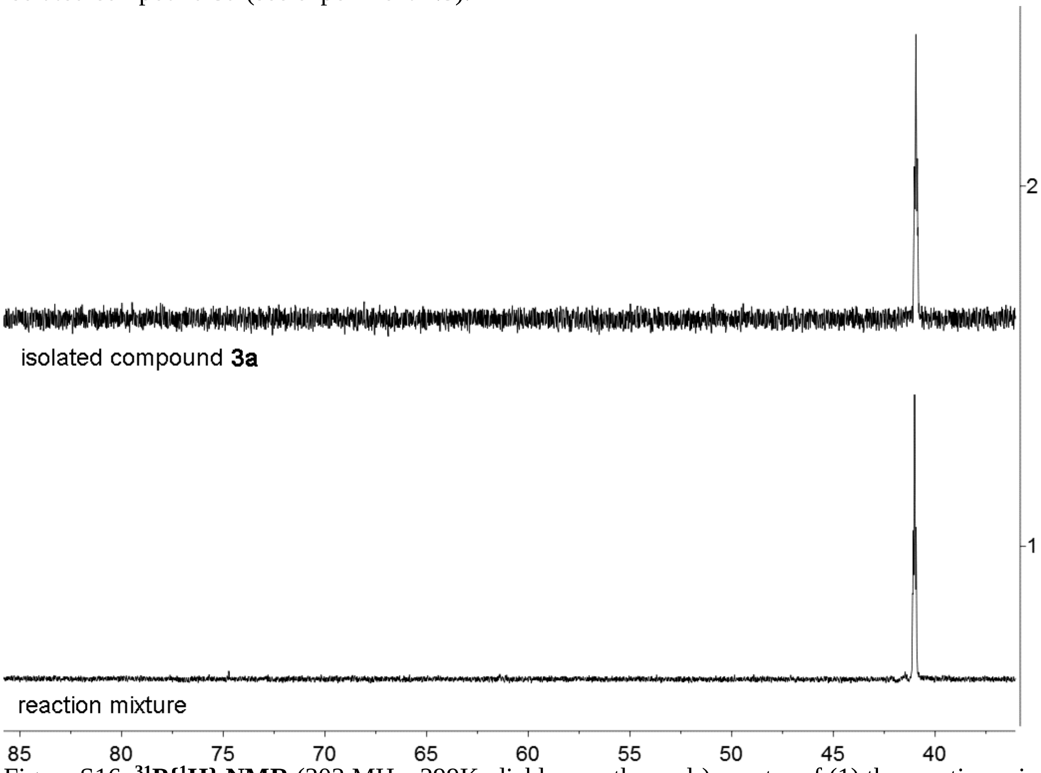

Figure S16. [31]**P{^{1}H} NMR** (202 MHz, 299K, dichloromethane-d_2) spectra of (1) the reaction mixture and (2) the isolated compound **3a** (see experiment 1.3).

2) Reactions of $PhPCy_2$ (**1b**) with $B(C_6F_5)_3$

2.1) Reaction of $PhPCy_2$ (**1b**) with $B(C_6F_5)_3$ in a H_2 atmosphere in the solid state: preparation of compound **4b**

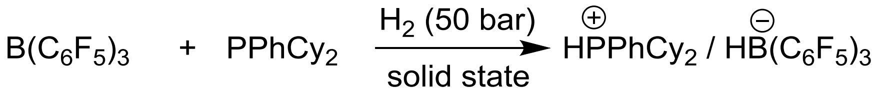

1b **4b**

Scheme S4. Reaction of $PhPCy_2$ (**1b**) with $B(C_6F_5)_3$ in the solid state: preparation of compound **4b**.

A mixture of $B(C_6F_5)_3$ (102.4 mg, 0.2 mmol) and $PhPCy_2$ (54.8 mg, 0.2 mmol) was put in a vial with a double valve adaptor[a] and stirred for 15 min. Then the reaction mixture was stirred in a dihydrogen atmosphere (50 bar) for 3 days. After the reaction time a part of the obtained powder (30 mg) was characterized by NMR experiments in solution (ca. 95% conversion).[1]
The remaining solid was crystallized from dichloromethane/pentane and dried in vacuo to give compound **4b** as a white solid (111.6 mg, 0.142 mmol, yield 71 %).

^{1}H NMR (500 MHz, 299K, dichloromethane-d_2) δ = 7.88 (m, 1H, *p*-Ph), 7.72 (m, 2H, *m*-Ph), 7.62 (m, 2H, *o*-Ph), 6.13 (dt, $^1J_{PH}$ = 459.3 Hz, $^3J_{HH}$ = 6.2 Hz, PH), 3.64 (br 1:1:1:1 q, $^1J_{BH}$ ~ 95 Hz, BH), 2.63 (m, 2H, CH^{Cy}), 2.03/1.36, 1.90/1.42, 1.86/1.37, 1.84/1.32, 1.77/1.21 (each m, each 2H, CH_2^{Cy})[a]. [[a] from the ^{1}H,^{13}C ghsqc experiment].
^{13}C{^{1}H} NMR (126 MHz, 299K, dichloromethane-d_2) δ = 136.4 (d, $^4J_{PC}$ = 2.9 Hz, *p*-Ph), 133.8 (d, $^2J_{PC}$ = 12.4 Hz, *o*-Ph), 131.3 (d, $^3J_{PC}$ = 8.8 Hz, *m*-Ph), 111.3 (d, $^1J_{PC}$ = 76.8 Hz, *i*-Ph), 29.2 (d, $^1J_{PC}$ = 41.1 Hz, CH^{Cy}), 27.7 (d, J_{PC} = 3.2 Hz), 26.7 (d, J_{PC} = 3.6 Hz), 26.0 (d, J_{PC} = 8.1 Hz), 25.9 (d, J_{PC} = 7.8 Hz), 25.1 (d, J_{PC} = 1.6 Hz)(CH_2^{Cy}), [C_6F_5 not listed].
^{11}B NMR (160 MHz, 299K, dichloromethane-d_2) δ = -25.3 (d, $^1J_{BH}$ ~ 94 Hz).
^{11}B{^{1}H} NMR (160 MHz, 299K, dichloromethane-d_2) δ = -25.3 ($\nu_{1/2}$ ~ 40 Hz).
^{19}F NMR (470 MHz, 299K, dichloromethane-d_2) δ = -133.9 (m, 2F, *o*-C_6F_5), -164.5 (t, $^3J_{FF}$ = 20.2 Hz, 1F, *p*-C_6F_5), -167.5 (m, 2F, *m*-C_6F_5), [$\Delta\delta^{19}F_{m,p}$ = 3.0].
^{31}P NMR (202 MHz, 299K, dichloromethane-d_2) δ = 30.6 (br d, $^1J_{PH}$ ~ 461 Hz).
^{31}P{^{1}H} NMR (202 MHz, 299K, dichloromethane-d_2) δ = 30.6 ($\nu_{1/2}$ ~ 5 Hz).

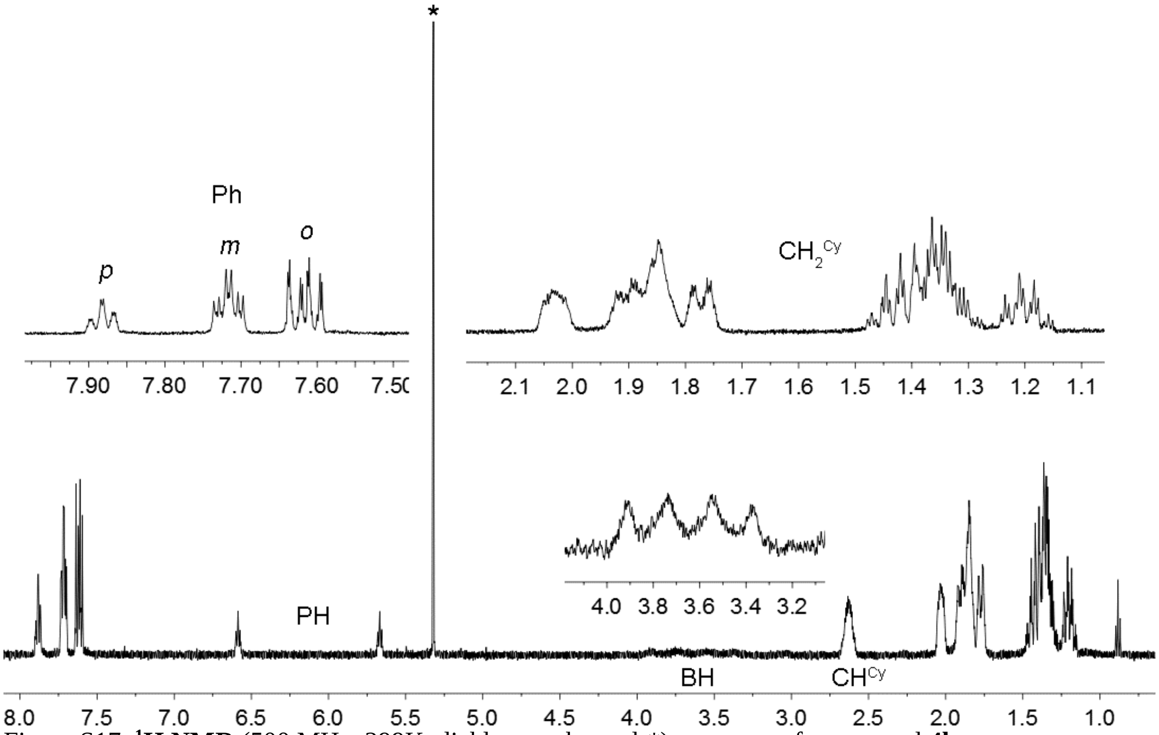

Figure S17. ^{1}**H NMR** (500 MHz, 299K, dichloromethane-d$_2$*) spectrum of compound **4b**.

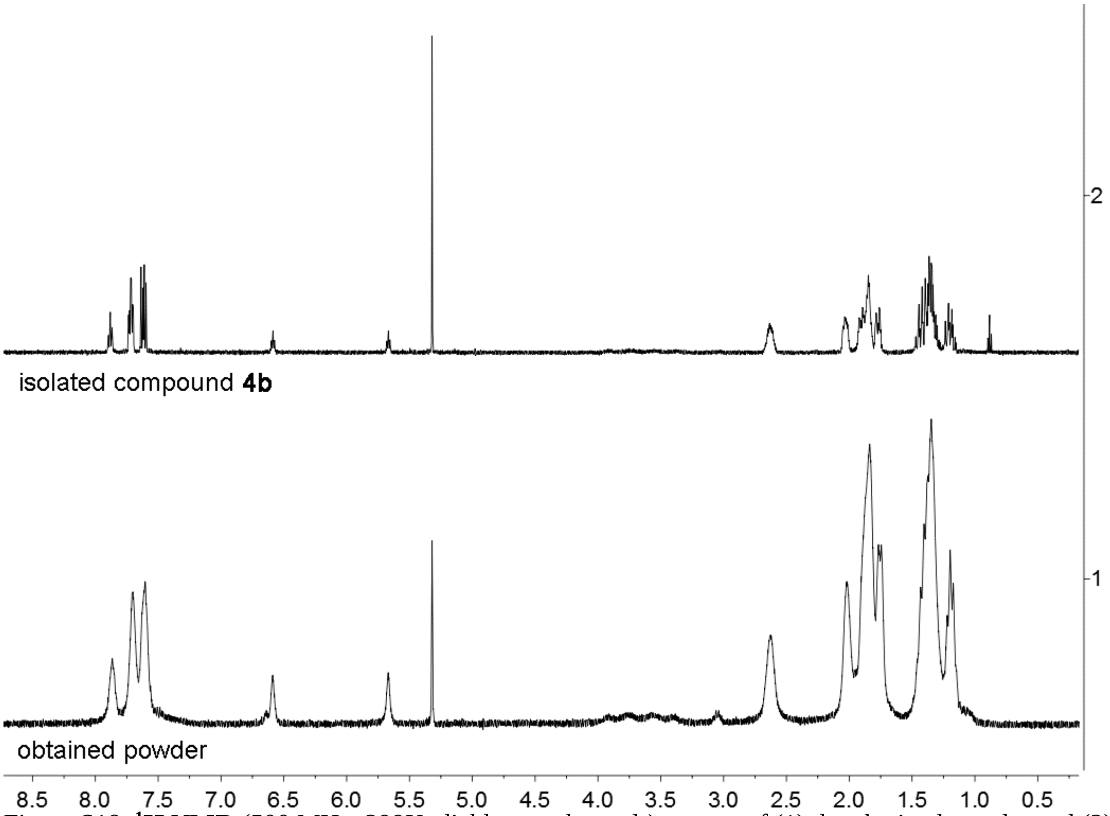

Figure S18. ^{1}**H NMR** (500 MHz, 299K, dichloromethane-d$_2$) spectra of (1) the obtained powder and (2) the isolated compound **4b**.

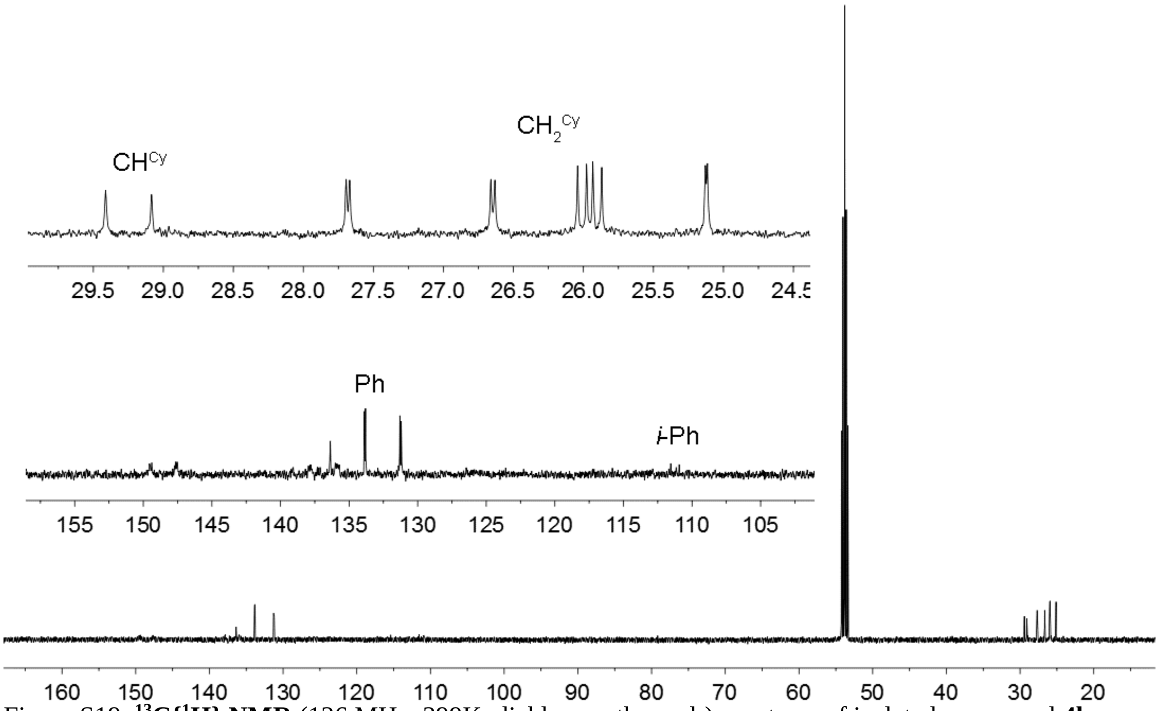

Figure S19. **^{13}C{^{1}H} NMR** (126 MHz, 299K, dichloromethane-d$_2$) spectrum of isolated compound **4b**.

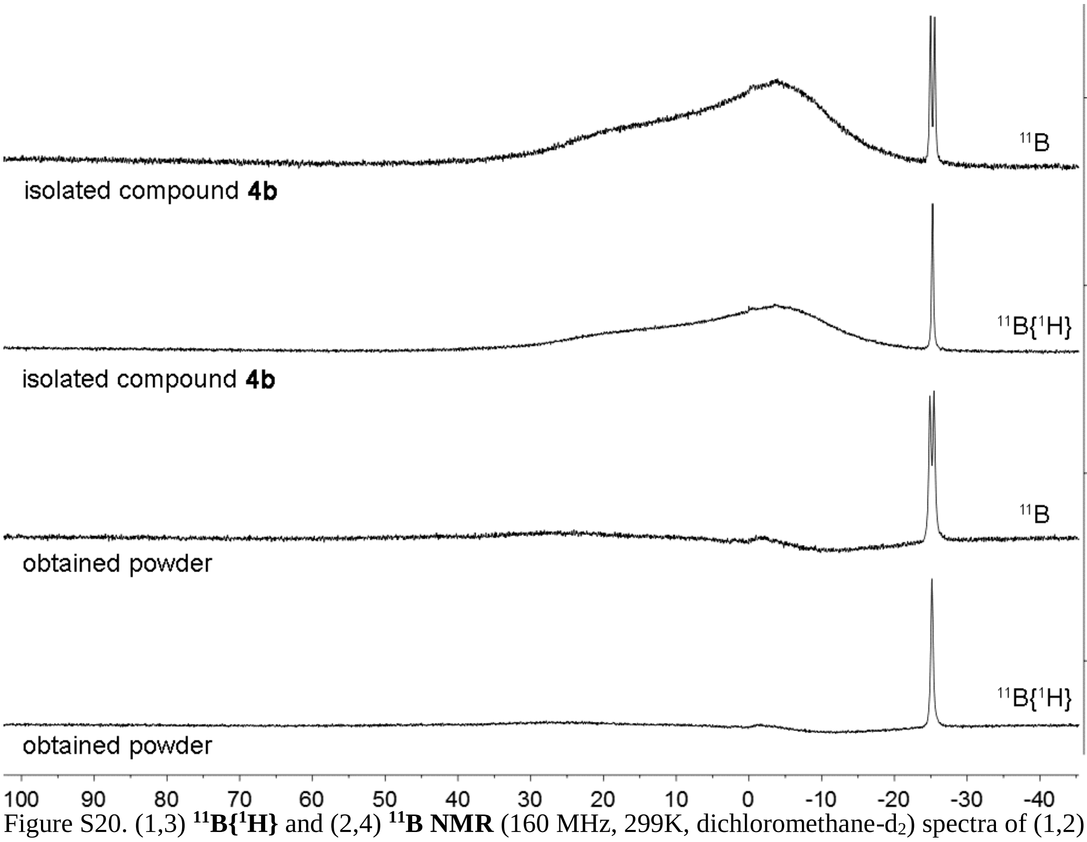

Figure S20. (1,3) **^{11}B{^{1}H}** and (2,4) **^{11}B NMR** (160 MHz, 299K, dichloromethane-d$_2$) spectra of (1,2) the obtained powder and (3,4) the isolated compound **4b**.

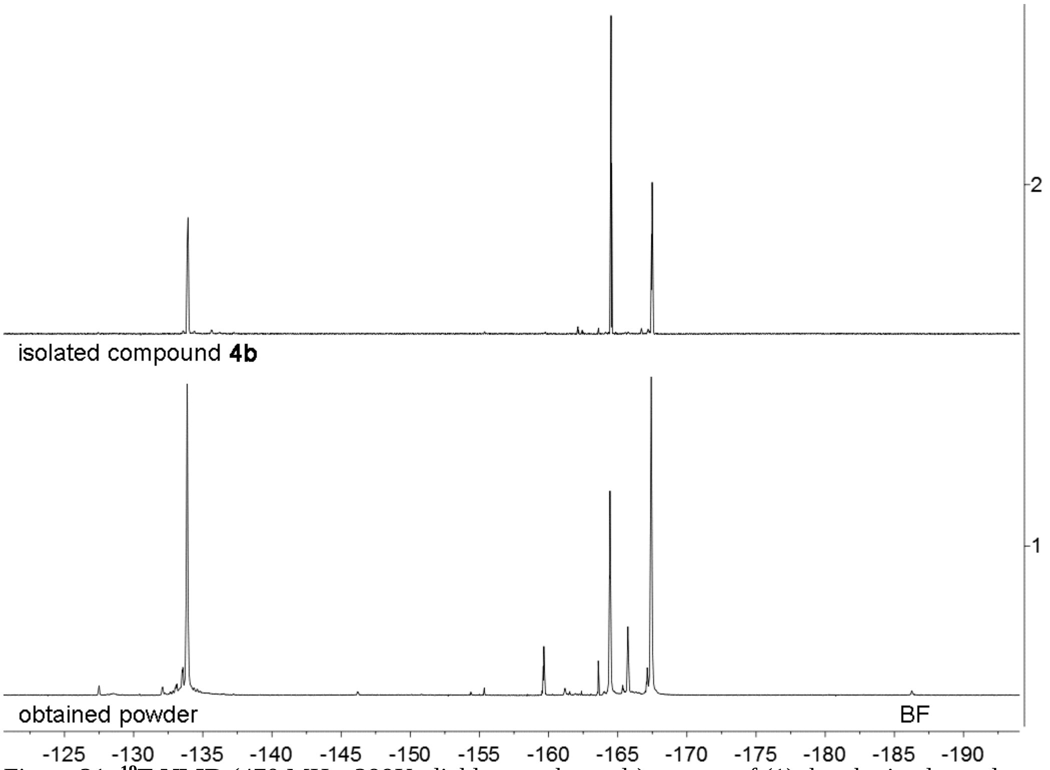

Figure 21. **^{19}F NMR** (470 MHz, 299K, dichloromethane-d_2) spectra of (1) the obtained powder and (2) the isolated compound **4b** [BF: tentatively assigned as $FB(C_6F_5)_3^-$].

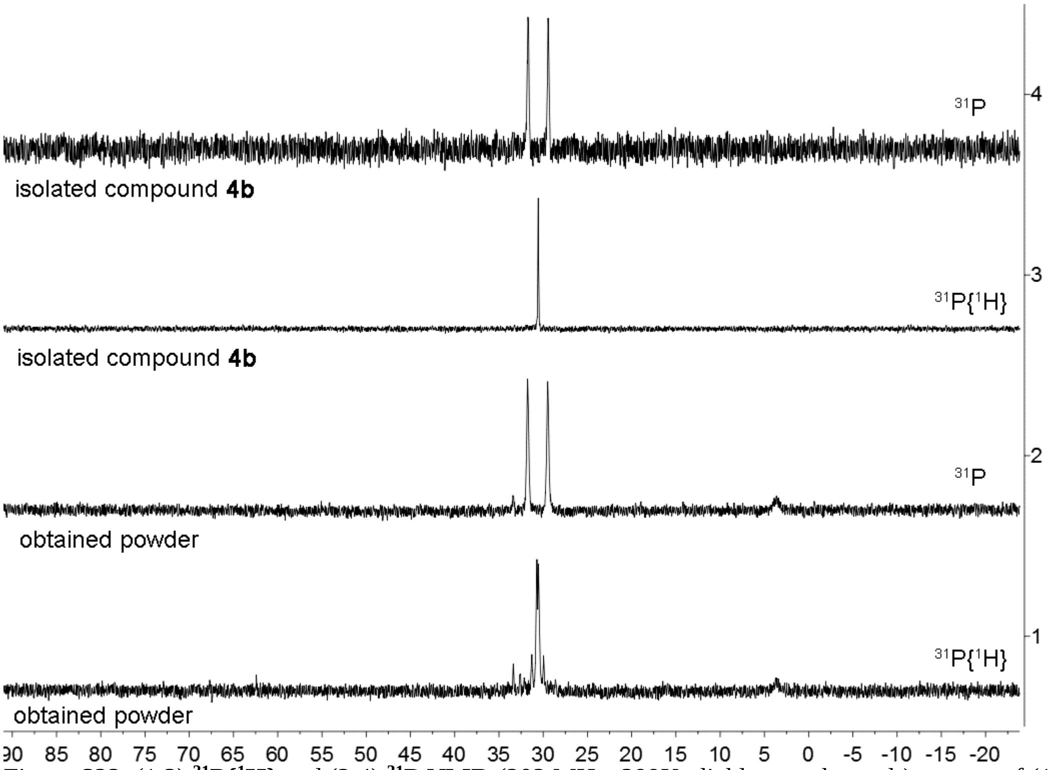

Figure S22. (1,3) **^{31}P{^{1}H}** and (2,4) **^{31}P NMR** (202 MHz, 299K, dichloromethane-d_2) spectra of (1,2) the obtained powder and (3,4) the isolated compound **4b**.

2.2) Reaction of $PhPCy_2$ (**1b**) with $B(C_6F_5)_3$ in solution: generation of compound **3b**

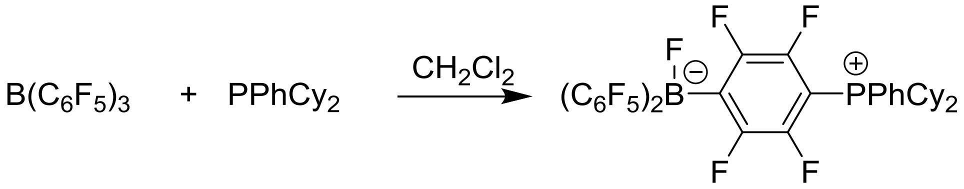

1b **3b**

Scheme S5. Reaction of $PhPCy_2$ (**1b**) with $B(C_6F_5)_3$ in solution: generation of compound **3b**.

A solution of $PhPCy_2$ (82.3 mg, 0.3 mmol) in CH_2Cl_2 (3 mL) was added to a solution of $B(C_6F_5)_3$ (153.6 mg, 0.3 mmol) in CH_2Cl_2 (3 mL). After the mixture was stirred at room temperature for 12 hours, all volatiles were removed in vacuo to give a white solid which was washed with pentane (1mL × 3) and dried in vacuo to finally give compound **3b** as a white solid (193.2 mg, 0.246 mmol, 82 %).

^{1}H NMR (600 MHz, 299K, dichloromethane-d_2) δ = 7.86 (m, 1H, *p*-Ph), 7.73 (m, 2H, *m*-Ph), 7.58 (m, 2H, *o*-Ph), 3.06 (m, 2H, CH^{Cy}), 2.01/1.24, 1.97/1.05, 1.94, 1.89, 1.49, 1.45, 1.76/1.12 (each m, each 2H, CH_2^{Cy}).

^{13}C{^{1}H} NMR (151 MHz, 299K, dichloromethane-d_2) δ = 135.6 (d, $^4J_{PC}$ = 3.1 Hz, *p*-Ph), 132.8 (d, $^2J_{PC}$ = 8.4 Hz, *o*-Ph), 130.5 (d, $^3J_{PC}$ = 12.2 Hz, *m*-Ph), 113.1 (d, $^1J_{PC}$ = 80.3 Hz, *i*-Ph), 31.2 (d, $^1J_{PC}$ = 43.9 Hz, CH^{Cy}), 26.23 (d, J_{PC} = 13.6 Hz), 26.17 (d, J_{PC} = 14.2 Hz), 25.8 (d, J_{PC} = 3.9 Hz), 25.7 (d, J_{PC} = 3.2 Hz), 25.3 (d, J_{PC} = 1.6 Hz)(CH_2^{Cy}), [C_6F_5 and C_6F_4 not listed].

^{11}B{^{1}H} NMR (192 MHz, 299K, dichloromethane-d_2) δ = -0.6 (br d, $^1J_{FB}$ ~ 70 Hz).

^{19}F NMR (564 MHz, 299K, dichloromethane-d_2) δ = -127.5 (br), -128.4 (br m)(each 2F, C_6F_4), -135.5 (m, 4F, *o*), -161.6 (m, $^3J_{FF}$ = 20.1 Hz, 2F, *p*), -166.5 (m, 4F, *m*)(C_6F_5) [$\Delta\delta^{19}F_{m,p}$ = 4.9], -192.9 (br m, 1F, BF).

^{31}P{^{1}H} NMR (243 MHz, 299K, dichloromethane-d_2) δ = 33.6 (m).

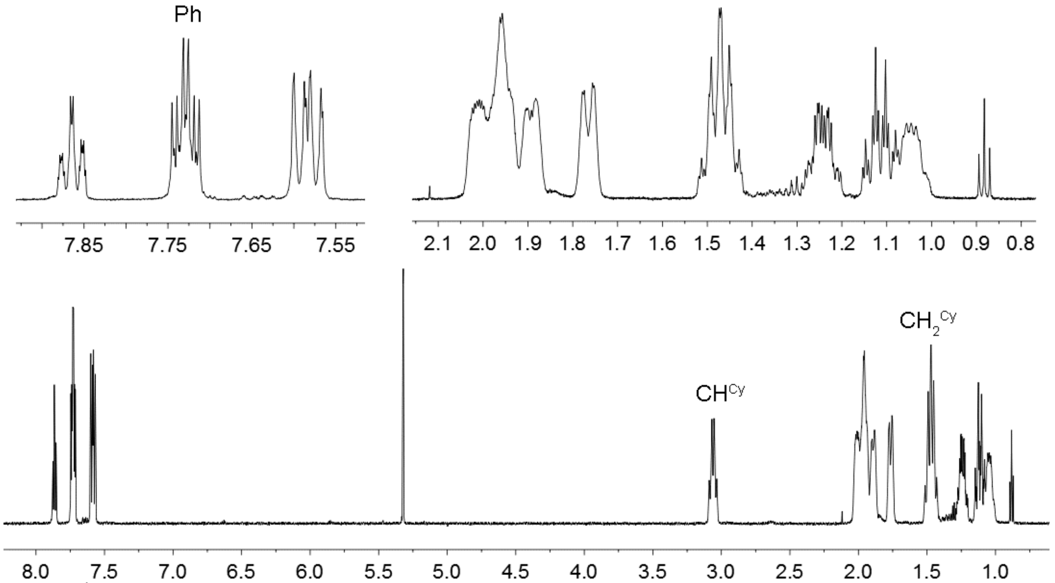

Figure S23. **^{1}H NMR** (600 MHz, 299K, dichloromethane-d_2) spectrum of compound **3b**.

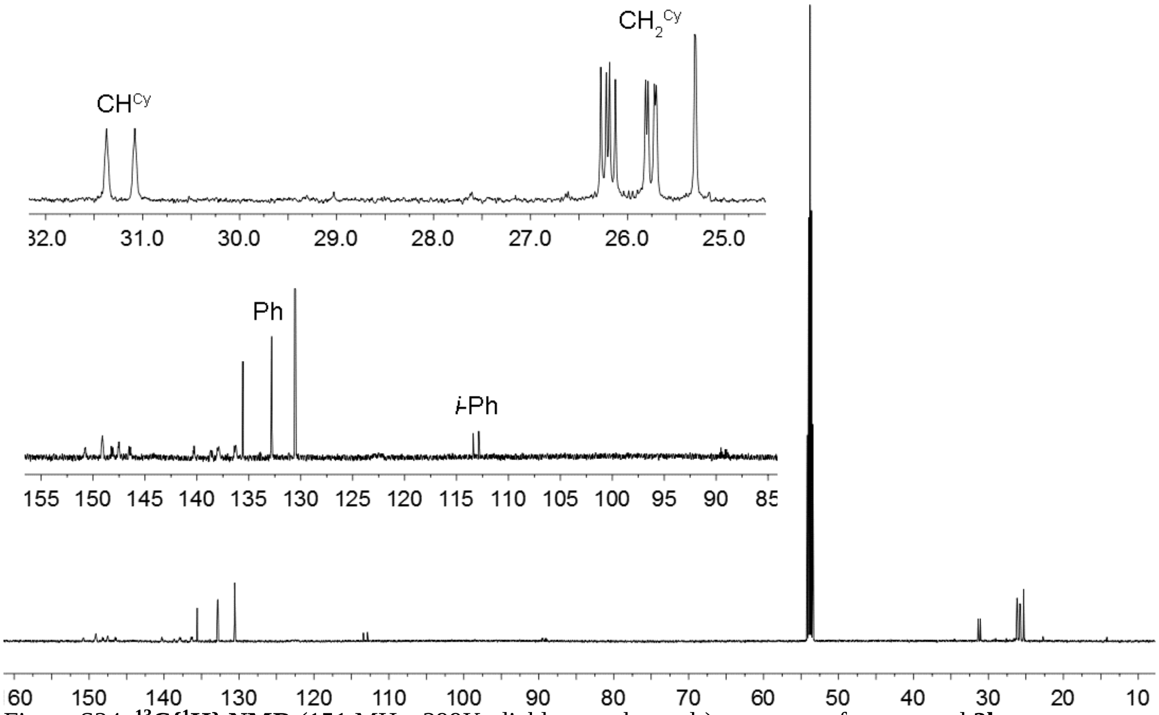

Figure S24. **$^{13}C\{^1H\}$ NMR** (151 MHz, 299K, dichloromethane-d_2) spectrum of compound **3b**

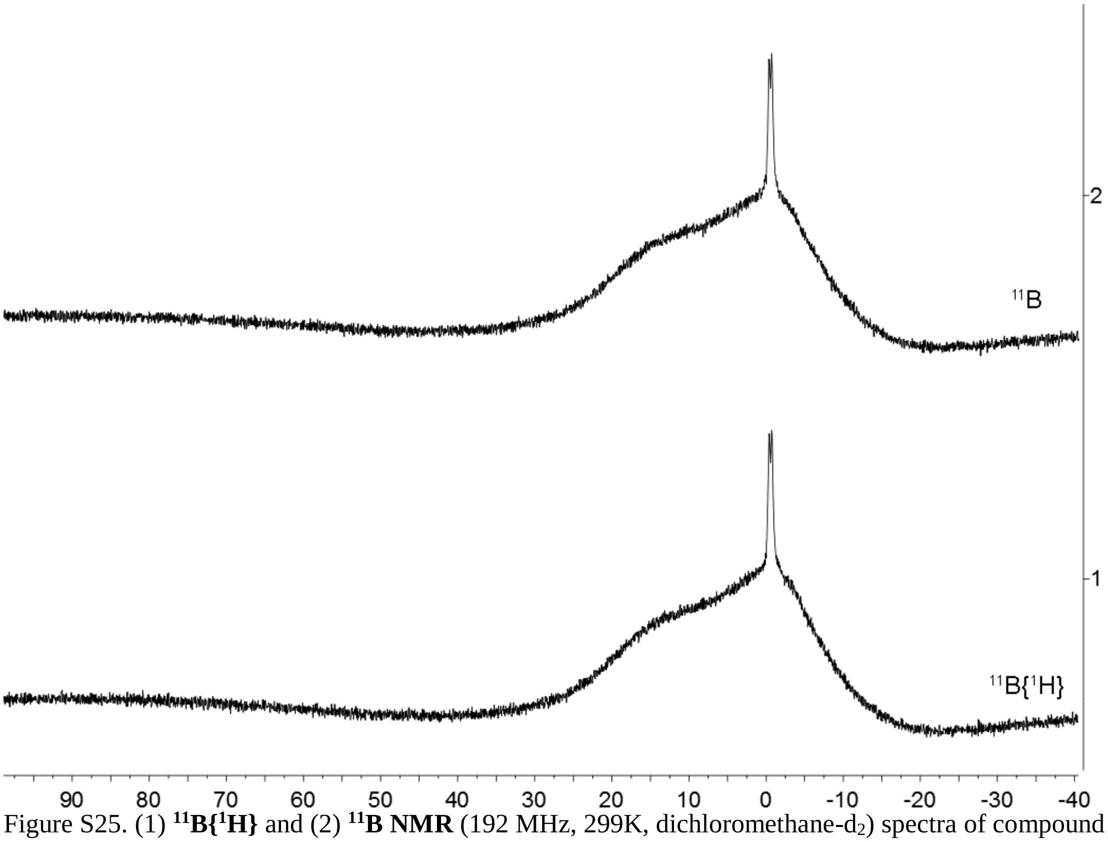

Figure S25. (1) **$^{11}B\{^1H\}$** and (2) **^{11}B NMR** (192 MHz, 299K, dichloromethane-d_2) spectra of compound **3b**.

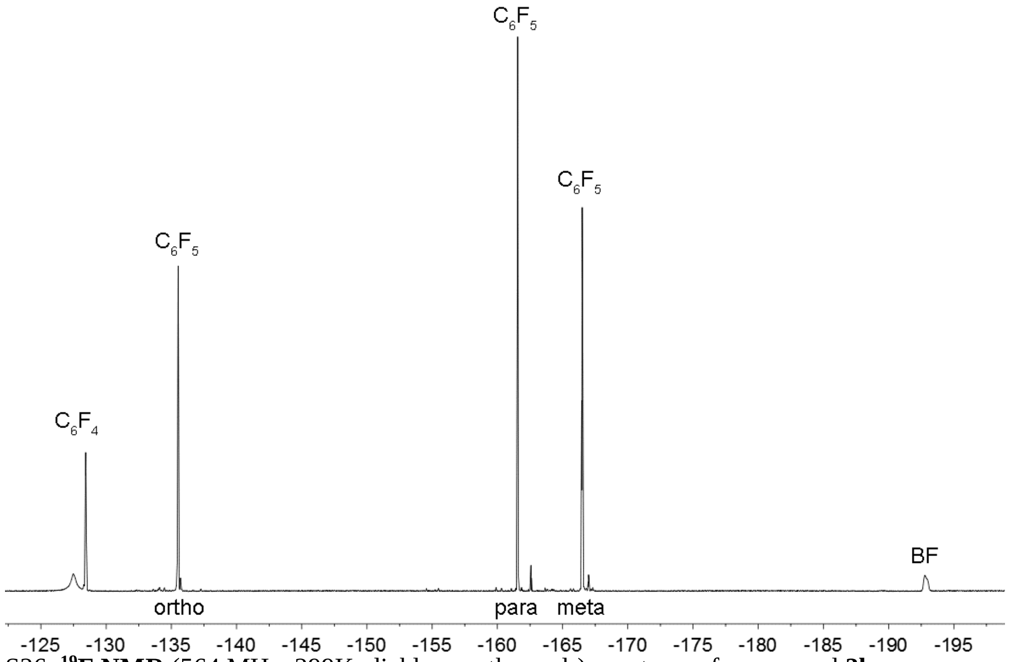

Figure S26. ^{19}**F NMR** (564 MHz, 299K, dichloromethane-d_2) spectrum of compound **3b**.

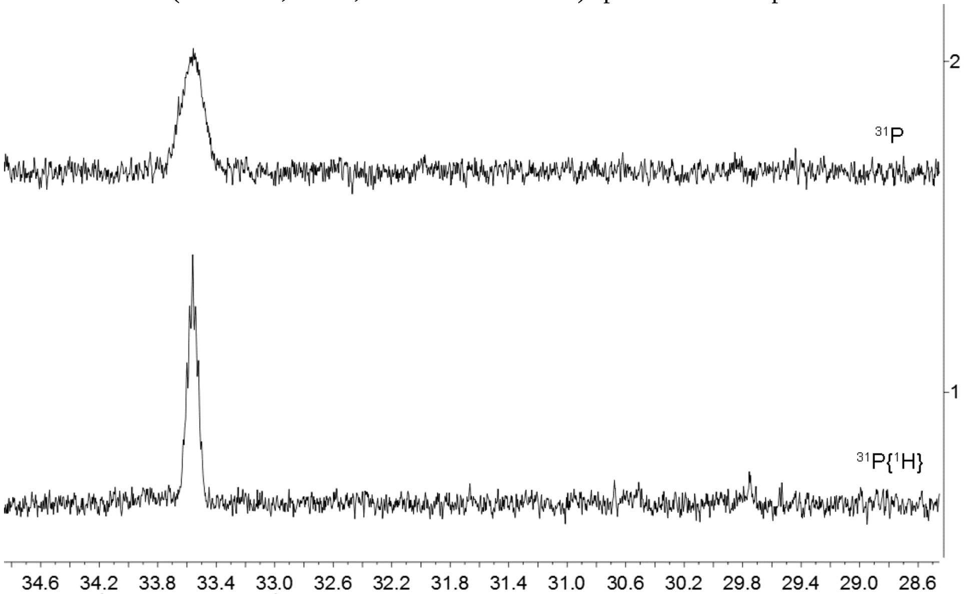

Figure S27. (1) ^{31}**P{^{1}H}** and (2) ^{31}**P NMR** (243 MHz, 299K, dichloromethane-d_2) spectra of compound **3b**.

2.3) Reaction of $PhPCy_2$ (**1b**) with $B(C_6F_5)_3$ in solution in a H_2 atmosphere: generation of a mixture of compounds **3b** and **4b**

A solution of $PhPCy_2$ (13.7 mg, 0.05 mmol) and $B(C_6F_5)_3$ (25.6 mg, 0.05 mmol) in CD_2Cl_2 (1 mL) was put in a vial with a double valve adaptor[a] and stirred for 12 hours in an H_2 atmosphere (50 bar). Then the reaction solution was characterized by NMR experiments.[1]

[Comment: the obtained NMR data showed a mixture of compounds **3b** and **4b** (ratio ca. 6 : 1 (^{1}H)]

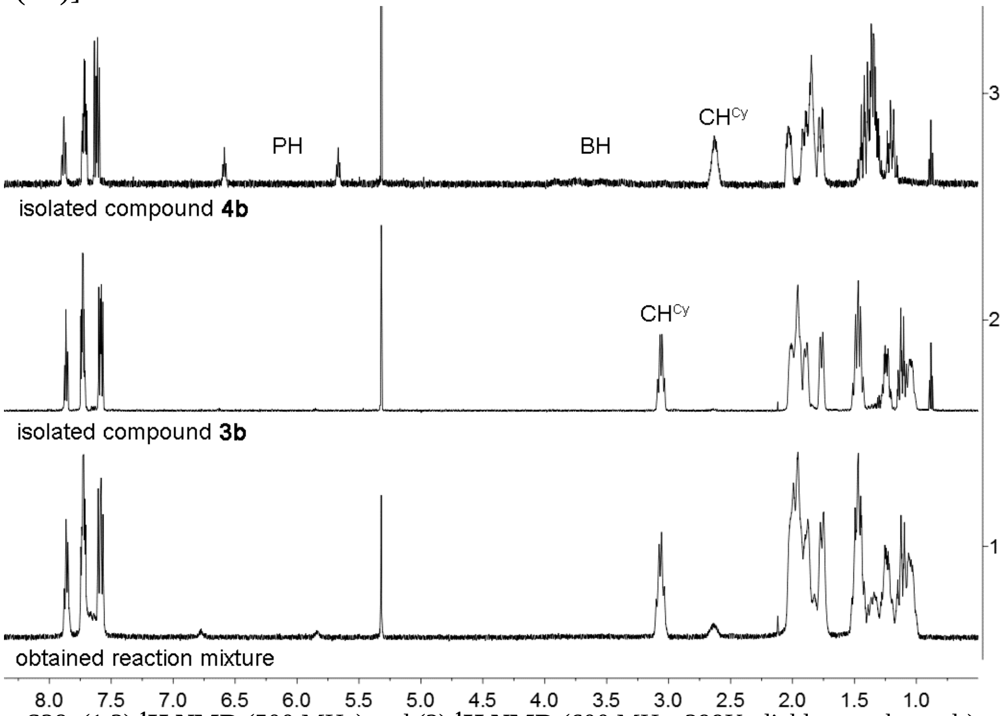

Figure S28. (1,3) **^{1}H NMR** (500 MHz) and (2) **^{1}H NMR** (600 MHz, 299K, dichloromethane-d$_2$) spectra of (1) the obtained reaction mixture, (2) the isolated compound **3b** and (3) the isolated compound **4b**.

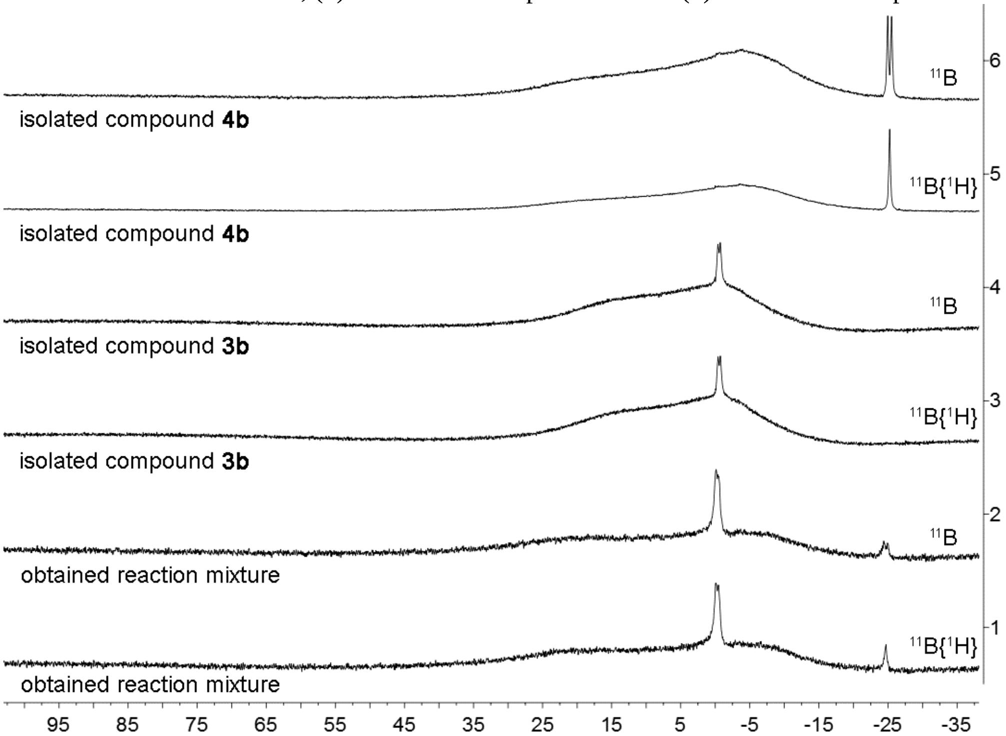

Figure S29. (1,5) **^{11}B{^{1}H}**/ (2,6) **^{11}B NMR** (160 MHz) and (3) **^{11}B{^{1}H}**/ (4) **^{11}B NMR** (192 MHz, 299K, dichloromethane-d$_2$) spectra of (1,2) the obtained reaction mixture, (3,4) the isolated compound **3b** and (5,6) the isolated compound **4b**.

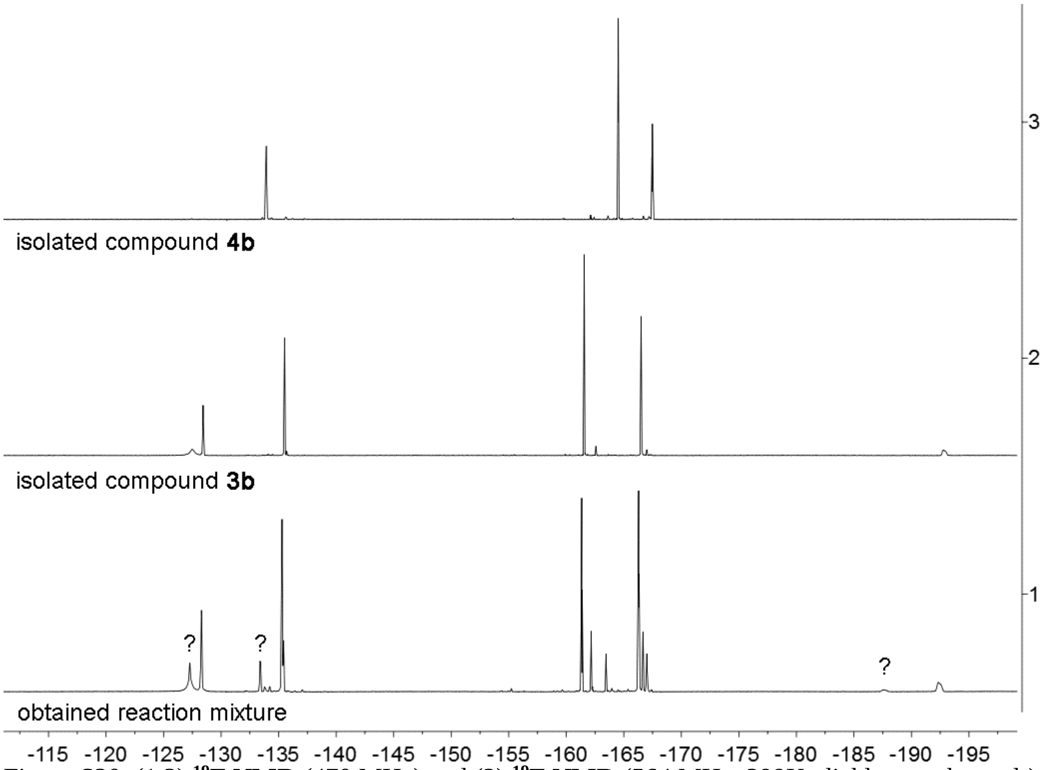

Figure S30. (1,3) ^{19}**F NMR** (470 MHz) and (2) ^{19}**F NMR** (564 MHz, 299K, dichloromethane-d_2) spectra of (1) the obtained reaction mixture, (2) the isolated compound **3b** and (3) the isolated compound **4b**.

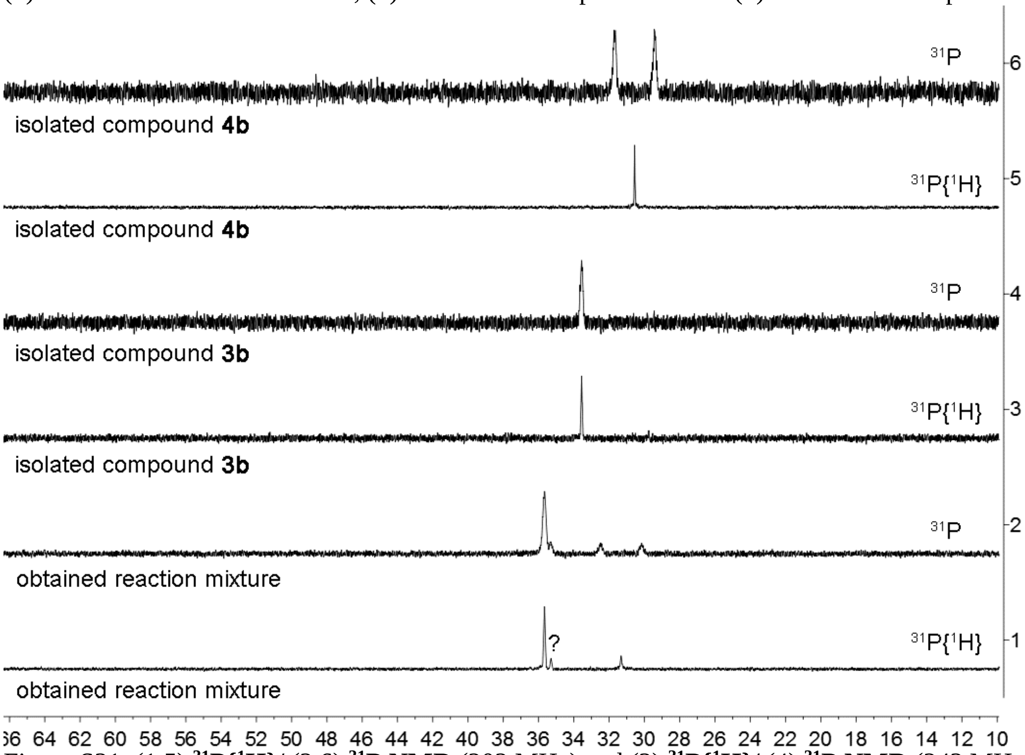

Figure S31. (1,5) ^{31}**P{^{1}H}**/ (2,6) ^{31}**P NMR** (202 MHz) and (3) ^{31}**P{^{1}H}**/ (4) ^{31}**P NMR** (243 MHz, 299K, dichloromethane-d_2) spectra of (1,2) the obtained reaction mixture, (3,4) the isolated compound **3b** and (5,6) the isolated compound **4b**.

3) Reactions of Ph_2P^tBu (**1c**) with $B(C_6F_5)_3$

3.1) Reaction of Ph_2P^tBu (**1c**) with $B(C_6F_5)_3$ in a H_2 atmosphere in the solid state: preparation of compound **4c**

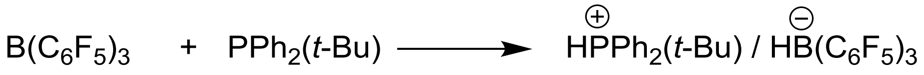

1c **4c**

Scheme S6. Reaction of Ph_2P^tBu (**1c**) with $B(C_6F_5)_3$ in the solid state: preparation of compound **4c**

A mixture of $B(C_6F_5)_3$ (102.4 mg, 0.2 mmol) and Ph_2P^tBu (48.5 mg, 0.2 mmol) was put in a vial with a double valve adaptor[a] and stirred for 15 min. Then the reaction mixture was stirred in a dihydrogen atmosphere (50 bar) for 3 days. After the reaction time a part of the obtained powder (30 mg) was characterized by NMR experiments in solution(ca 95% conversion).[1]

The remaining solid was crystallized from dichloromethane/pentane and dried in vacuo to give compound **4c** as a white solid (90.5 mg, 0.120 mmol, yield 60 %).

^{1}H NMR (500 MHz, 299K, dichloromethane-d_2) δ = 7.89 (m, 2H, *p*-Ph), 7.82 (m, 4H, *o*-Ph), 7.73 (m, 4H, *m*-Ph), 7.12 (d, $^1J_{PH}$ = 473.7 Hz, 1H, PH), 3.67 (br 1:1:1:1q, $^1J_{BH}$ ~ 90.9 Hz, 1H, BH), 1.48 (d, $^3J_{PH}$ = 19.1 Hz, 9H, *t*-Bu).
^{13}C{^{1}H} NMR (126 MHz, 299K, dichloromethane-d_2) δ = 148.6 (dm, $^1J_{FC}$ ~ 235 Hz, C_6F_5), 138.2 (dm, $^1J_{FC}$ ~ 245 Hz, C_6F_5), 136.8 (dm, $^1J_{FC}$ ~ 250 Hz, C_6F_5), 125.3 (i- C_6F_5), 136.4 (d, $^4J_{PC}$ = 3.0 Hz, *p*-Ph), 133.9 (d, $^2J_{PC}$ = 9.5 Hz, *o*-Ph), 131.3 (d, $^3J_{PC}$ = 12.4 Hz, *m*-Ph), 114.1 (d, $^1J_{PC}$ = 78.3 Hz, *i*-Ph), 33.4 (d, $^1J_{PC}$ = 41.5 Hz, *t*-Bu), 25.7 (d, $^2J_{PC}$ = 2.0 Hz, *t*-Bu).
^{11}B NMR (160 MHz, 299K, dichloromethane-d_2) δ = -25.2 (d, $^1J_{BH}$ ~ 91 Hz).
^{11}B{^{1}H} NMR (160 MHz, 299K, dichloromethane-d_2) δ = -25.2 ($\nu_{1/2}$ ~ 40 Hz).
^{19}F NMR (470 MHz, 299K, dichloromethane-d_2) δ = -133.9 (m, 2F, *o*-C_6F_5), -164.5 (t, $^3J_{FF}$ = 20.2 Hz, 1F, *p*-C_6F_5), -167.5 (m, 2F, *m*-C_6F_5), [$\Delta\delta^{19}F_{m,p}$ = 3.0].
^{31}P NMR (202 MHz, 299K, dichloromethane-d_2) δ = 31.4 (br d, $^1J_{PH}$ ~ 474 Hz).
^{31}P{^{1}H} NMR (202 MHz, 299K, dichloromethane-d_2) δ = 31.4 ($\nu_{1/2}$ ~ 2 Hz).

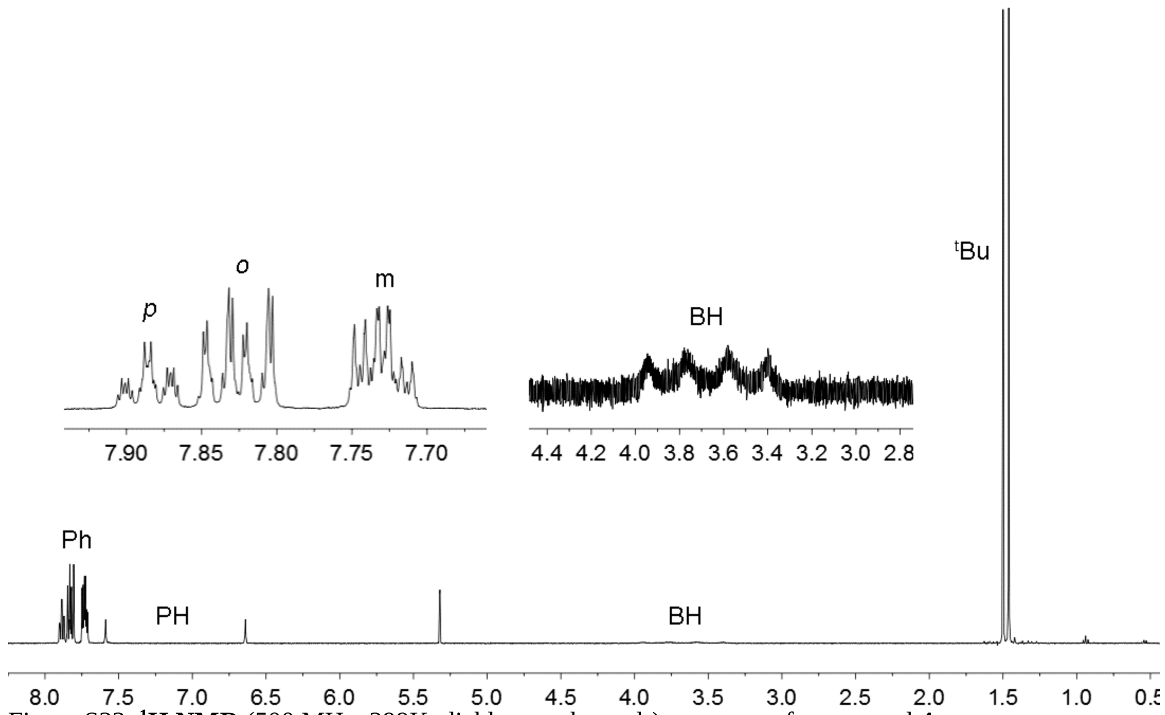

Figure S32. ^{1}**H NMR** (500 MHz, 299K, dichloromethane-d_2) spectrum of compound **4c**.

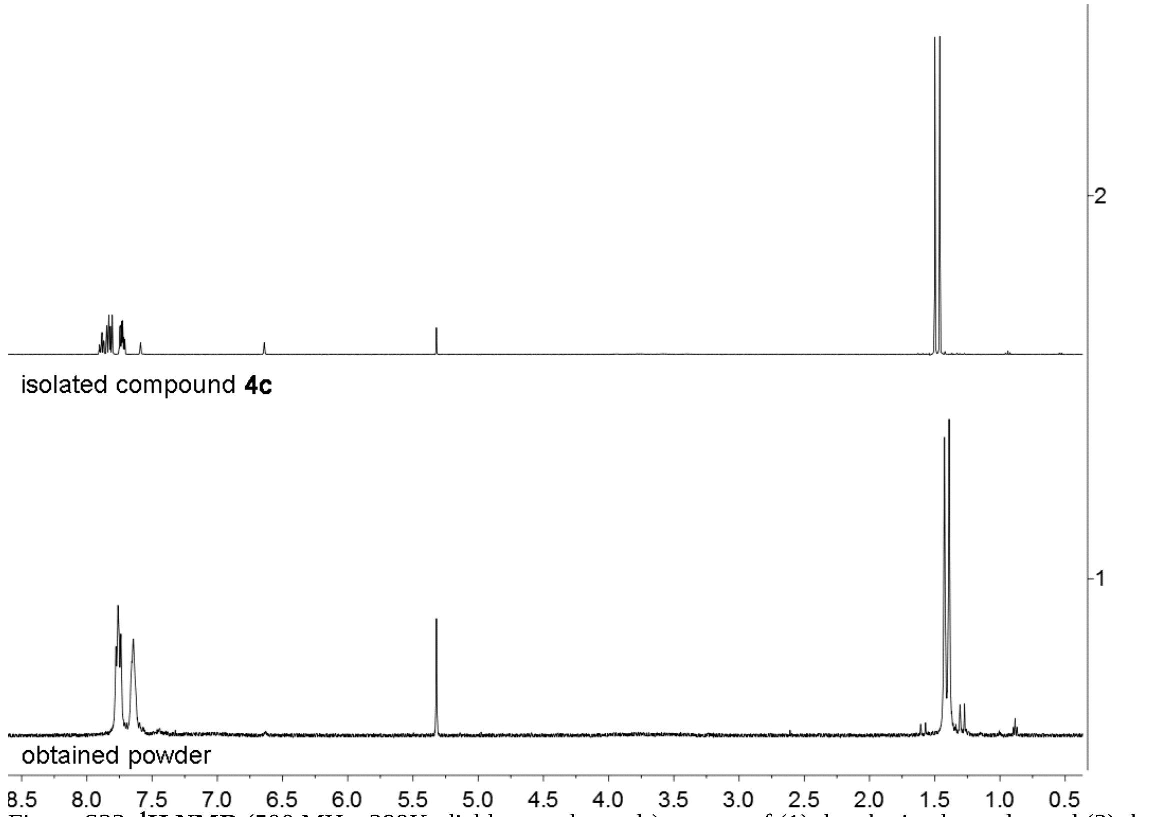

Figure S33. ^{1}**H NMR** (500 MHz, 299K, dichloromethane-d_2) spectra of (1) the obtained powder and (2) the isolated compound **4c**.

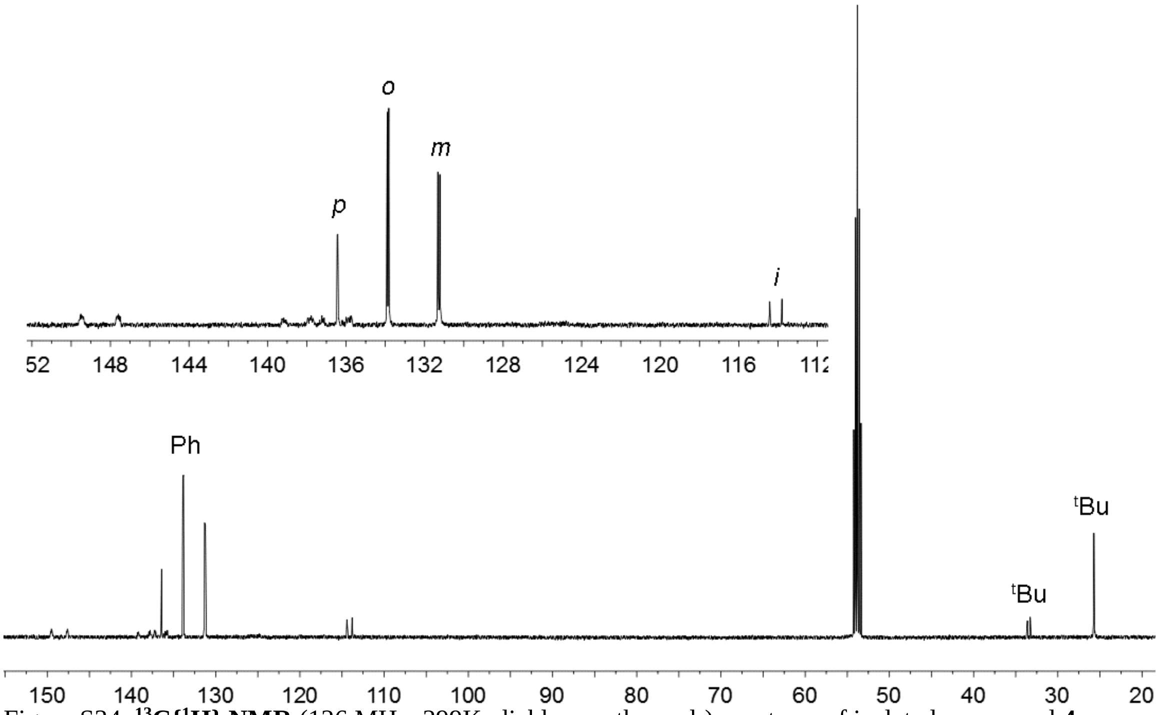

Figure S34. **$^{13}C\{^1H\}$ NMR** (126 MHz, 299K, dichloromethane-d_2) spectrum of isolated compound **4c**.

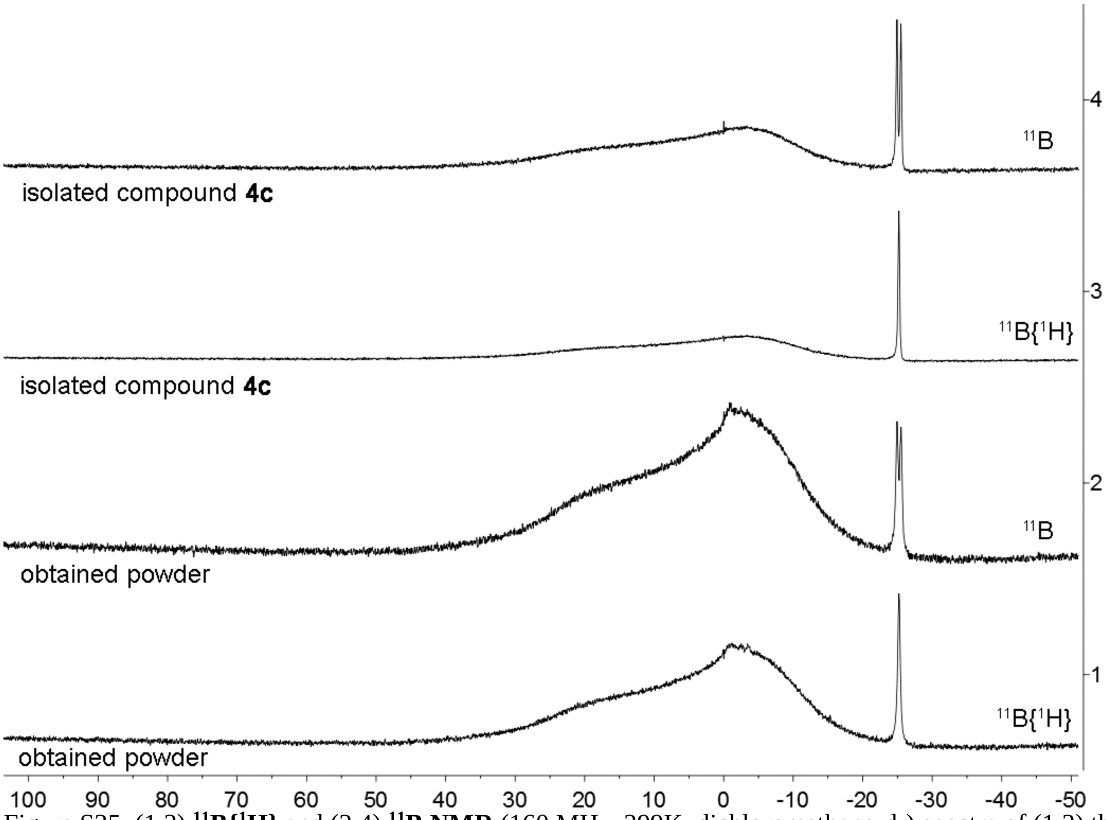

Figure S35. (1,3) **$^{11}B\{^1H\}$** and (2,4) **^{11}B NMR** (160 MHz, 299K, dichloromethane-d_2) spectra of (1,2) the obtained powder and (3,4) the isolated compound **4c**.

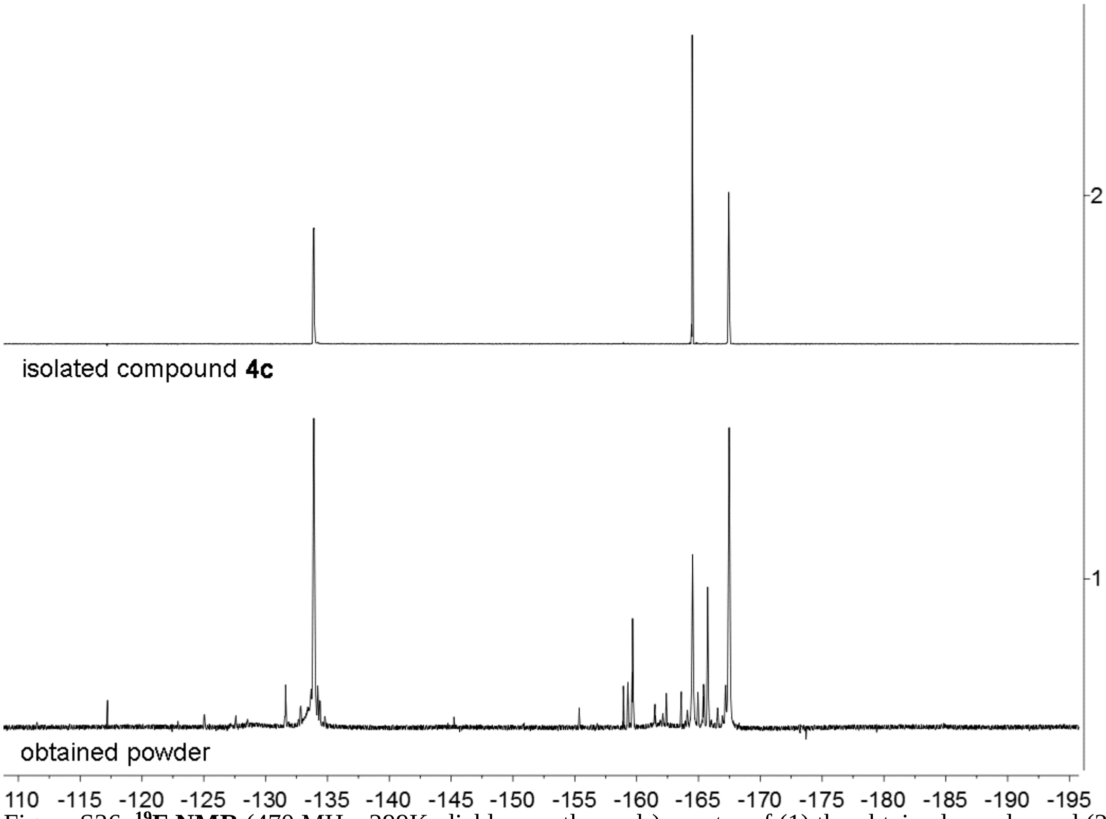

Figure S36. **^{19}F NMR** (470 MHz, 299K, dichloromethane-d_2) spectra of (1) the obtained powder and (2) the isolated compound **4c**.

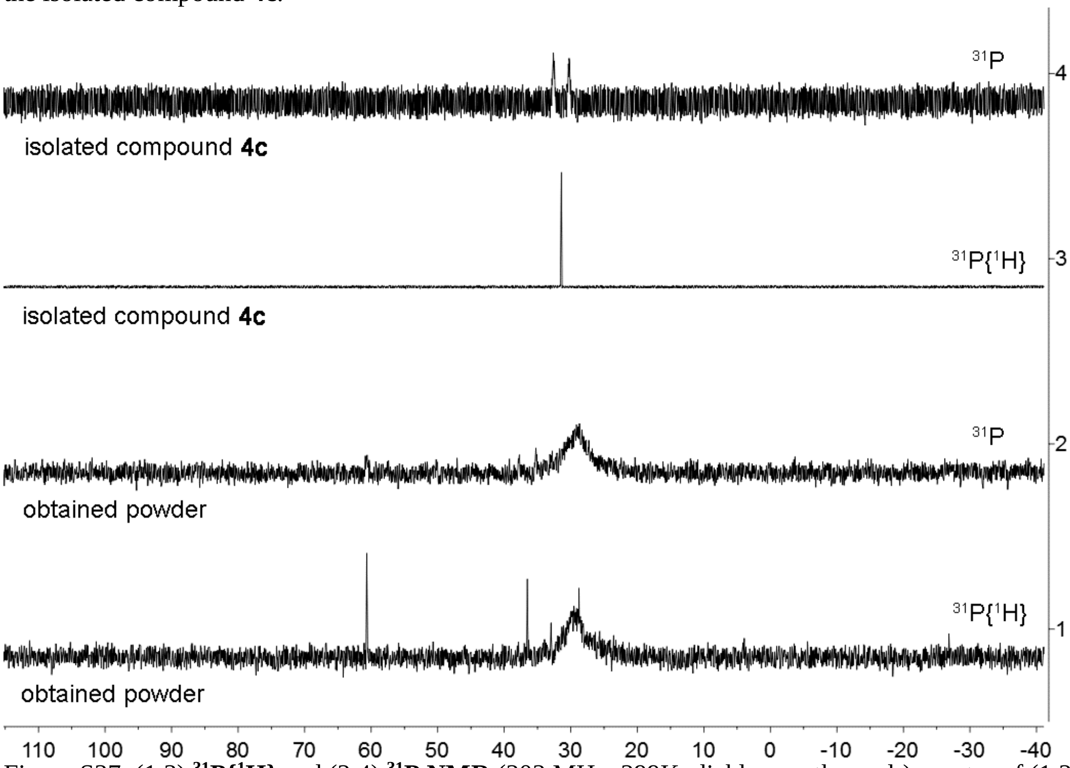

Figure S37. (1,3) **^{31}P{^{1}H}** and (2,4) **^{31}P NMR** (202 MHz, 299K, dichloromethane-d_2) spectra of (1,2) the obtained powder and (3,4) the isolated compound **4c**.

3.2) Reaction of Ph_2P^tBu (**1c**) with $B(C_6F_5)_3$ in solution: generation of compound **3c**

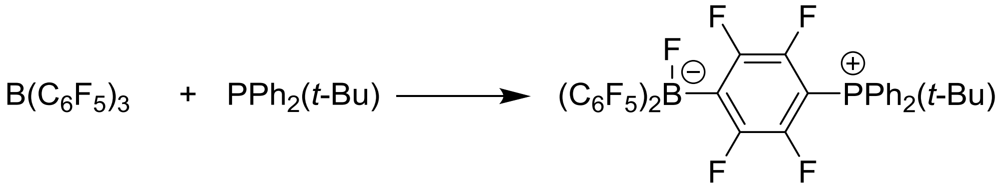

Scheme S7. Reaction of Ph_2P^tBu (**1c**) with $B(C_6F_5)_3$ in solution: generation of compound **3c**

A solution of Ph_2P^tBu (72.7 mg, 0.3 mmol) in CH_2Cl_2 (3 mL) was added to a solution of $B(C_6F_5)_3$ (153.6 mg, 0.3 mmol) in CH_2Cl_2 (3 mL). After the mixture was stirred at room temperature for 12 hours, all volatiles were removed in vacuo to give a white solid which was washed with pentane (1mL × 3) and dried in vacuo to finally give compound **3c** as a white solid (172.0 mg, 0.228 mmol, 76 %).

^{1}H NMR (500 MHz, 299K, dichloromethane-d_2) δ = 7.86 (m, 2H, *p*-Ph), 7.71 (m, 8H, *o,m*-Ph), 1.60 (d, $^3J_{PH}$ = 18.6 Hz, 9H, tBu).

^{13}C{^{1}H} NMR (126 MHz, 299K, dichloromethane-d_2) δ = 135.7 (*p*-Ph), 133.7 (d, J_{PC} = 9.2 Hz), 130.9 (d, J_{PC} = 12.6 Hz)(*o,m*-Ph), 117.7 (d, $^1J_{PC}$ = 81.7 Hz, *i*-Ph), 37.4 (d, $^1J_{PC}$ = 40.6 Hz, tBu), 27.8 (br m, tBu), [C_6F_5 and C_6F_4 not listed].

^{11}B{^{1}H} NMR (160 MHz, 299K, dichloromethane-d_2) δ = -0.6 (br d, $^1J_{FB}$ ~ 68 Hz).

^{19}F NMR (470 MHz, 299K, dichloromethane-d_2) δ = -124.0, -128.6 (each br m, each 2F, C_6F_4), -135.5 (m, 4F, *o*), -161.5 (t, $^3J_{FF}$ = 20.2 Hz, 2F, *p*), -166.5 (m, 4F, *m*)(C_6F_5)[$\Delta\delta^{19}F_{m,p}$ = 5.0], -192.9 (br m, 1F, BF),.

^{31}P{^{1}H} NMR (202 MHz, 299K, dichloromethane-d_2) δ = 33.3 (m).

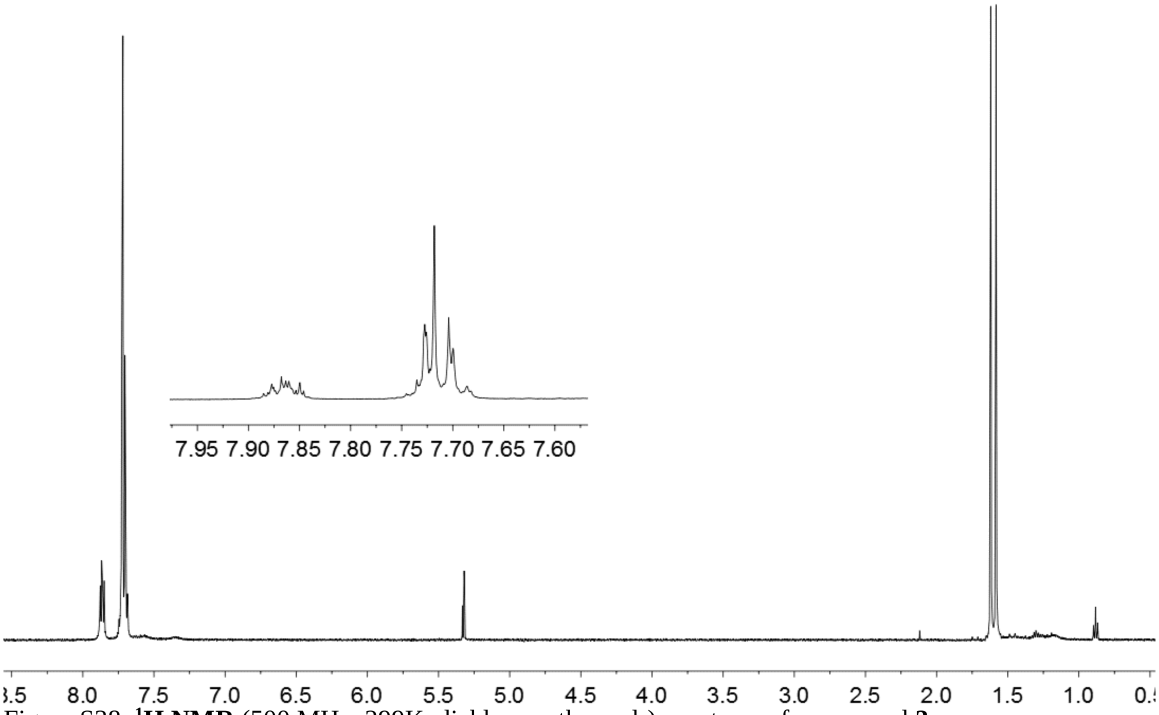

Figure S38. **^{1}H NMR** (500 MHz, 299K, dichloromethane-d_2) spectrum of compound **3c**.

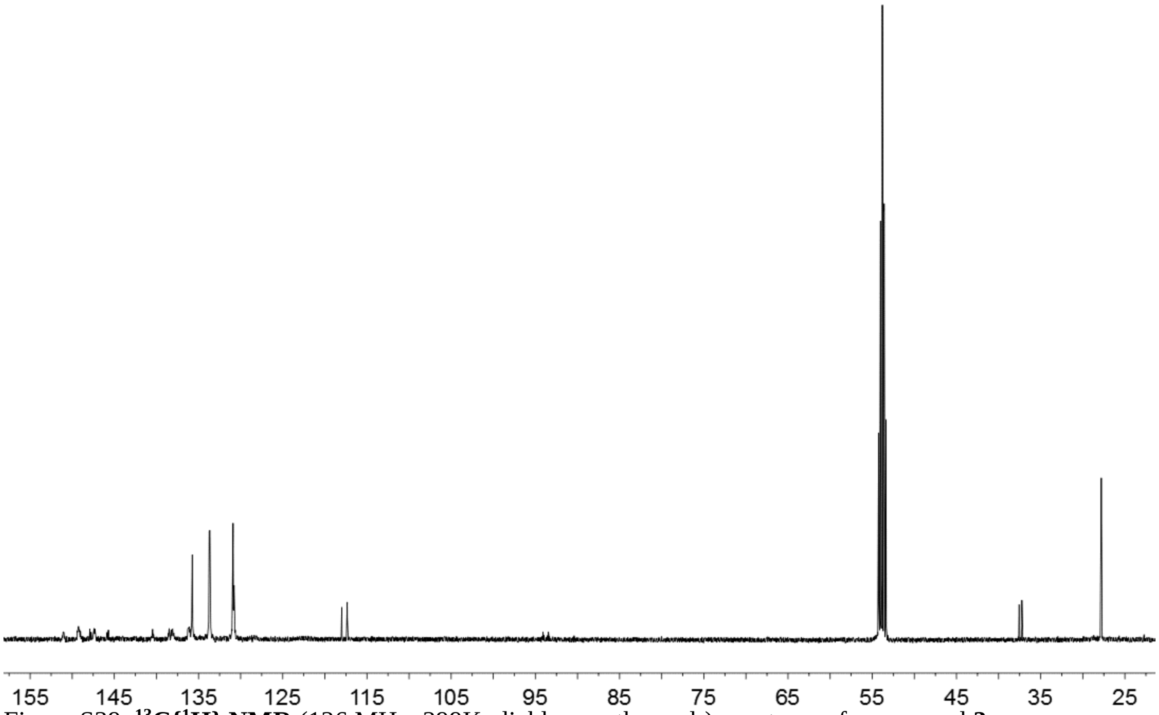

Figure S39. **$^{13}C\{^1H\}$ NMR** (126 MHz, 299K, dichloromethane-d_2) spectrum of compound **3c**

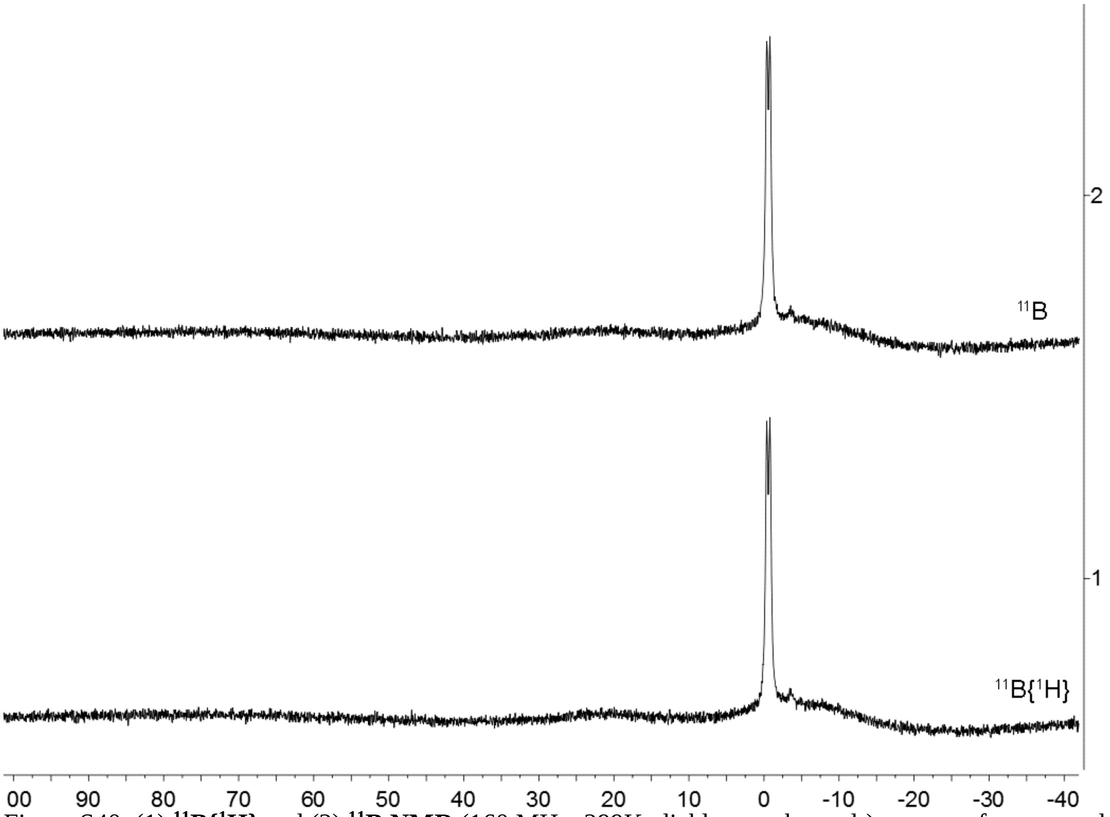

Figure S40. (1) **$^{11}B\{^1H\}$** and (2) **^{11}B NMR** (160 MHz, 299K, dichloromethane-d_2) spectra of compound **3c**.

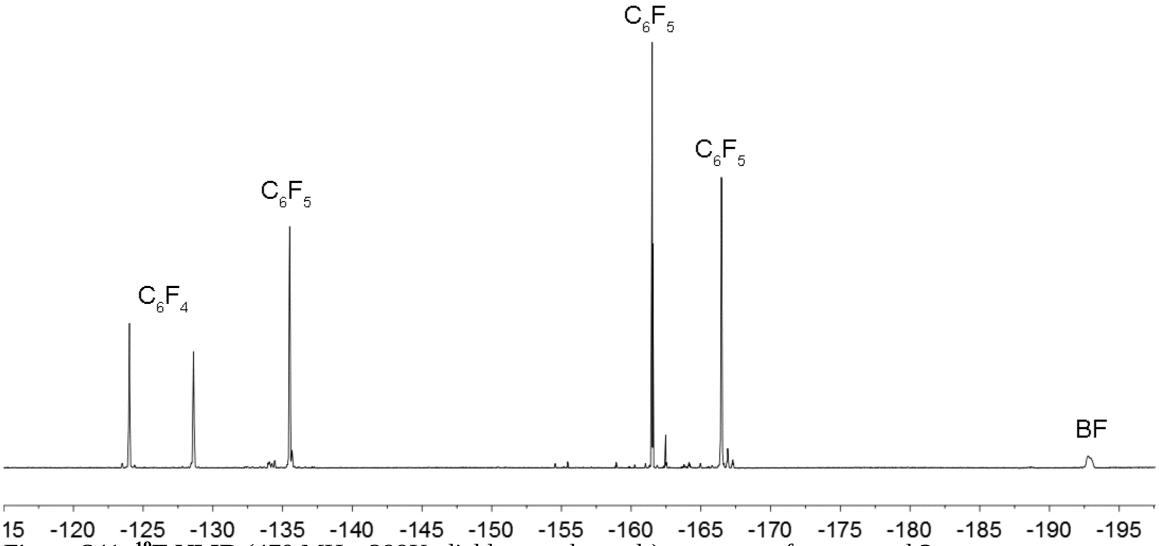

Figure S41. ^{19}**F NMR** (470 MHz, 299K, dichloromethane-d_2) spectrum of compound **3c**.

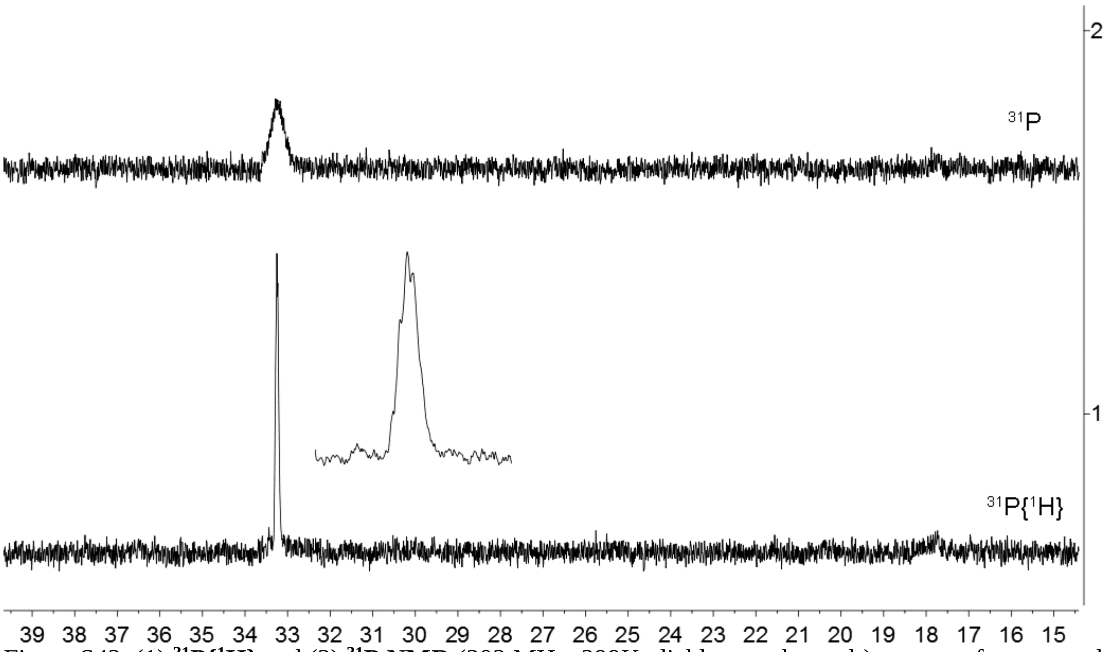

Figure S42. (1) ^{31}**P{^{1}H}** and (2) ^{31}**P NMR** (202 MHz, 299K, dichloromethane-d_2) spectra of compound **3c**.

<u>3.3) Reaction of Ph_2P^tBu (**1c**) with $B(C_6F_5)_3$ in solution in a H_2 atmosphere: generation of a mixture of compounds **3c** and **4c**</u>

A solution of Ph_2P^tBu (12.1 mg, 0.05 mmol) and $B(C_6F_5)_3$ (25.6 mg, 0.05 mmol) in CD_2Cl_2 (1 mL) was put in a vial with a double valve adaptor[a] and stirred for 12 hours in an H_2 atmosphere (50 bar). Then the reaction solution was characterized by NMR experiments.[1]

[Comment: the obtained NMR data showed a mixture of compounds **3c** and **4c** (ratio ca. 2:1 (^{11}B))]

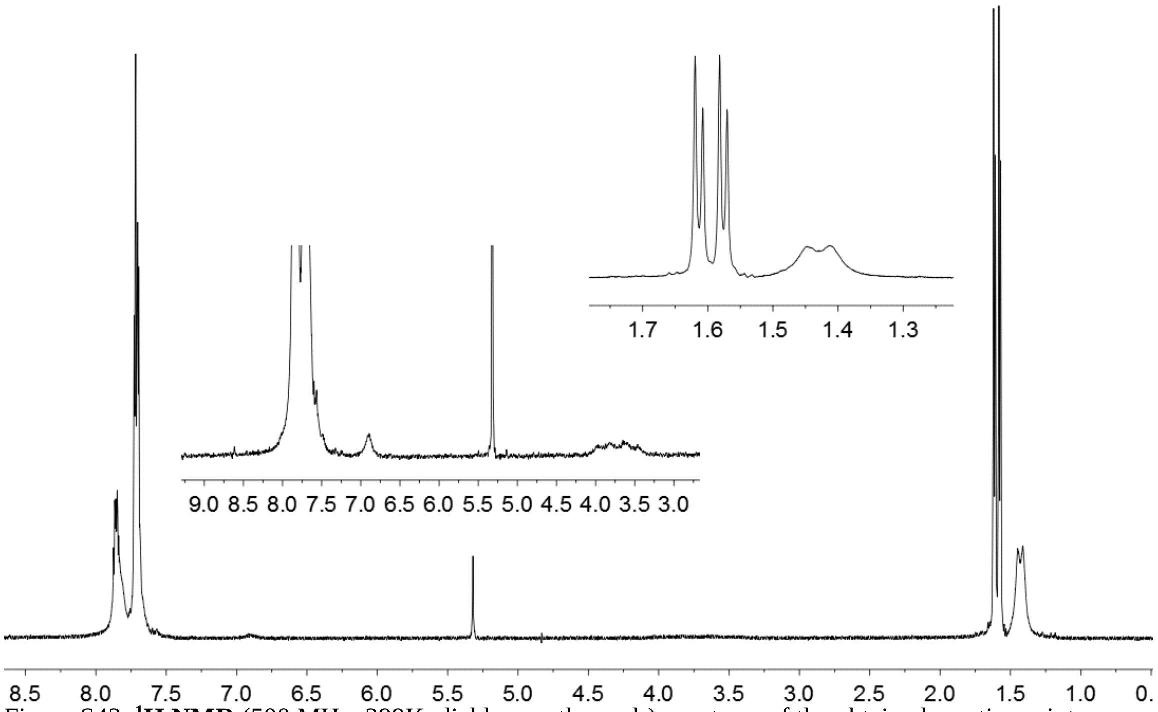

Figure S43. ^{1}**H NMR** (500 MHz, 299K, dichloromethane-d_2) spectrum of the obtained reaction mixture.

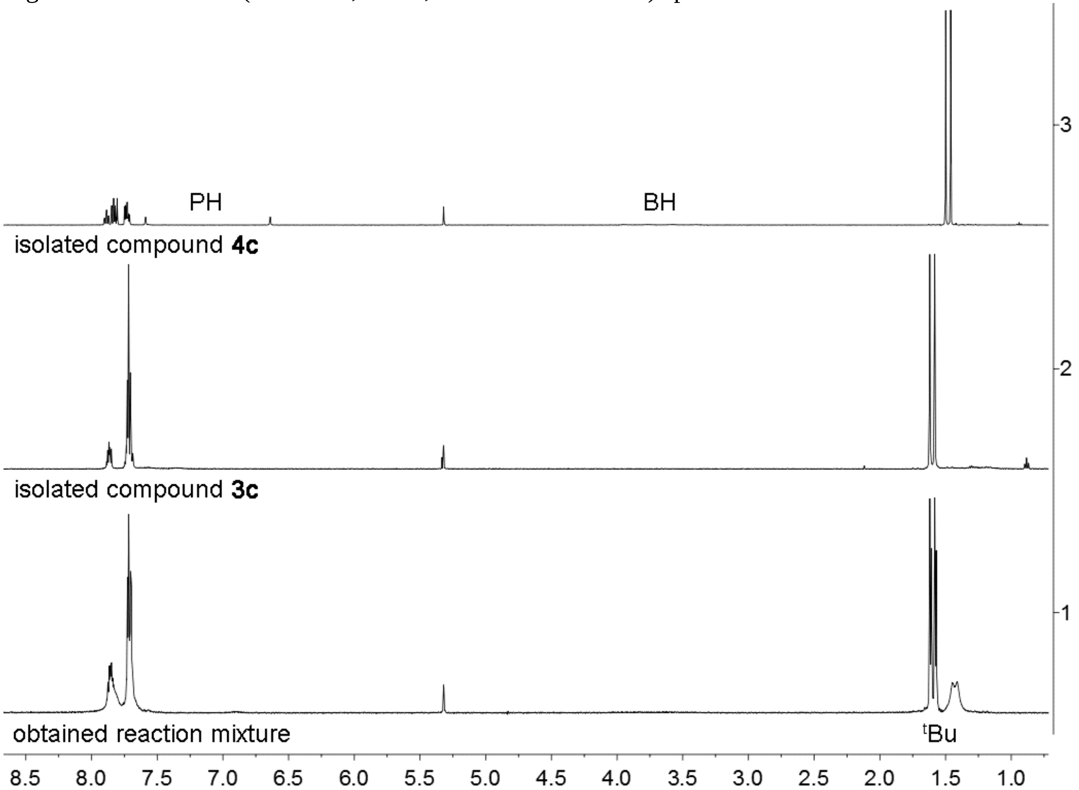

Figure S44. ^{1}**H NMR** (500 MHz, 299K, dichloromethane-d_2) spectra of (1) the obtained reaction mixture, (2) the isolated compound **3c** and (3) the isolated compound **4c**.

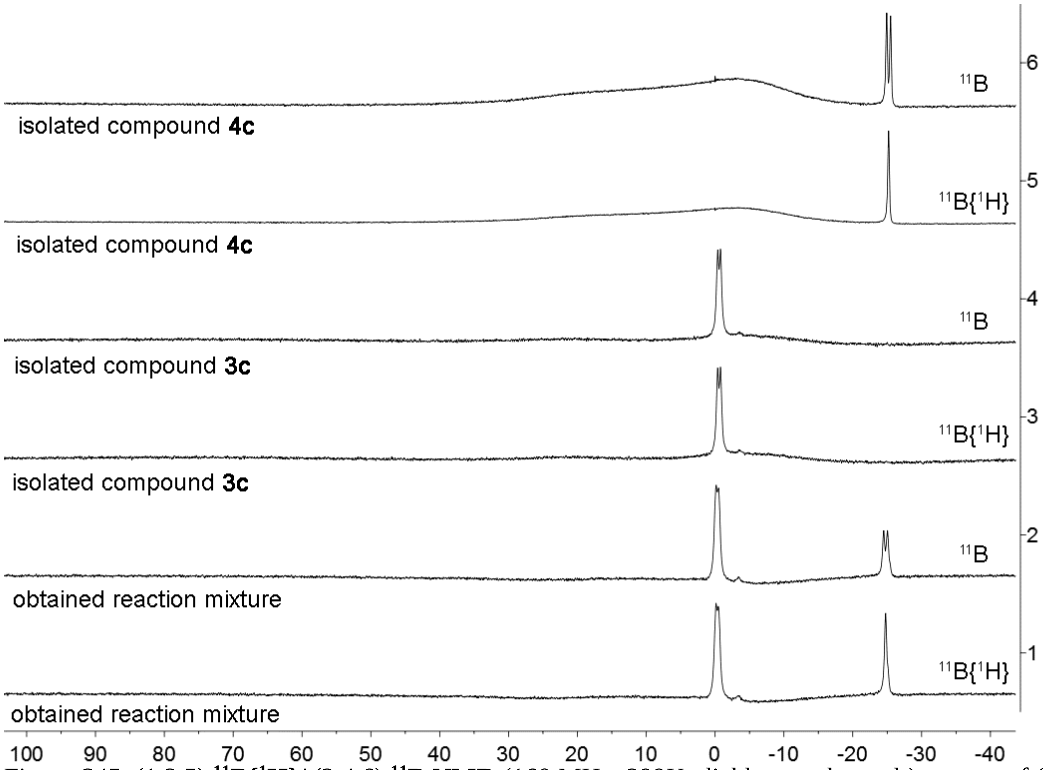

Figure S45. (1,3,5) **^{11}B{^{1}H}**/ (2,4,6) **^{11}B NMR** (160 MHz, 299K, dichloromethane-d_2) spectra of (1,2) the obtained reaction mixture, (3,4) the isolated compound **3c** and (5,6) the isolated compound **4c**.

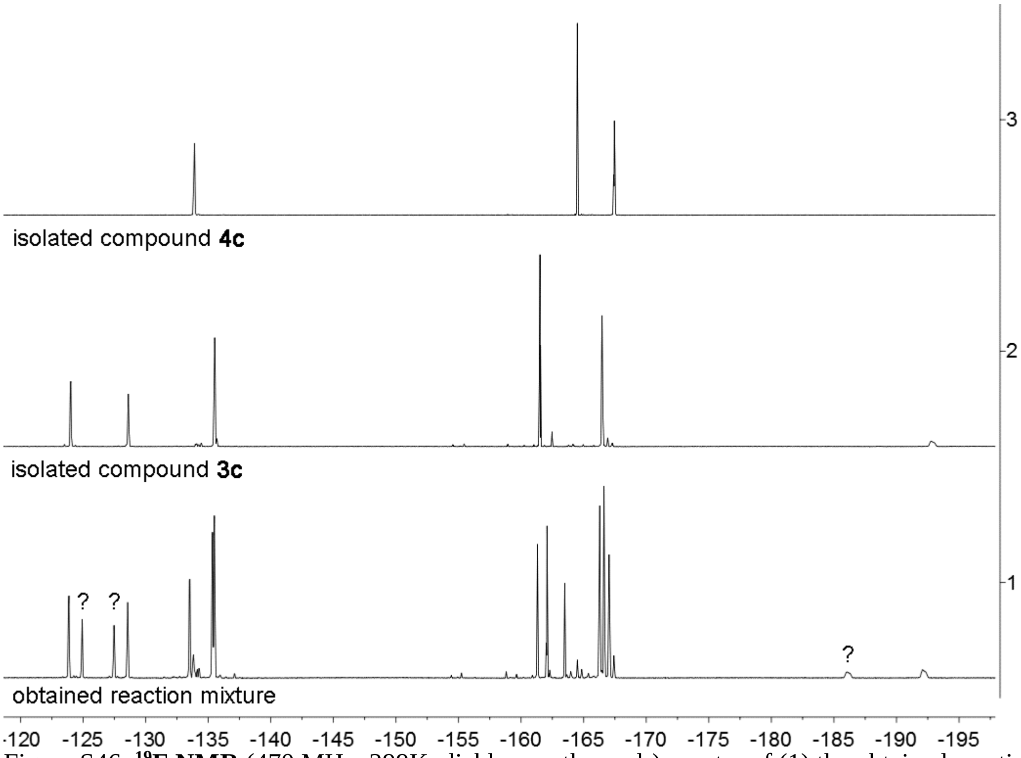

Figure S46. **^{19}F NMR** (470 MHz, 299K, dichloromethane-d_2) spectra of (1) the obtained reaction mixture ((the signals marked ? are likely due to subsequent BH vs BF exchange), (2) the isolated compound **3c** and (3) the isolated compound **4c**.

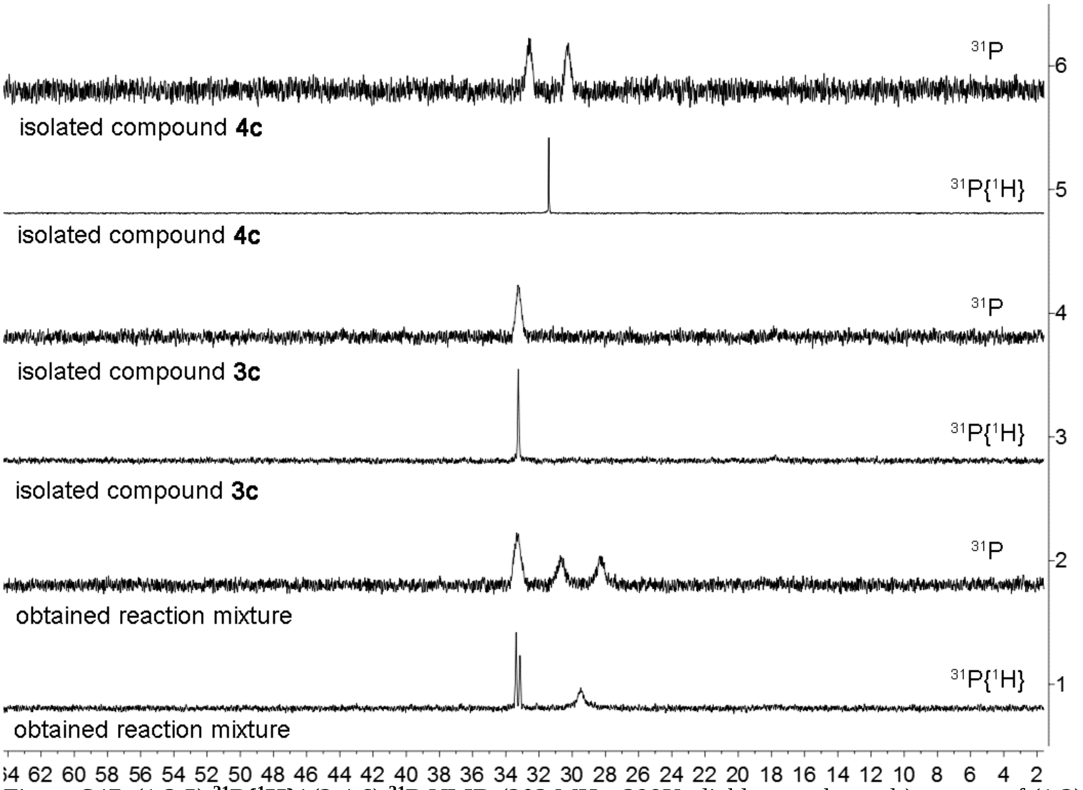

Figure S47. (1,3,5) **^{31}P{^{1}H}**/ (2,4,6) **^{31}P NMR** (202 MHz, 299K, dichloromethane-d_2) spectra of (1,2) the obtained reaction mixture, (3,4) the isolated compound **3c** and (5,6) the isolated compound **4c**

4) Reactions of PCy_3 (**1a**) and $B(C_6F_5)_3$ with SO_2

4.1) Reaction of PCy_3 (**1a**) and $B(C_6F_5)_3$ with SO_2 in the solid state: preparation of compound **5**

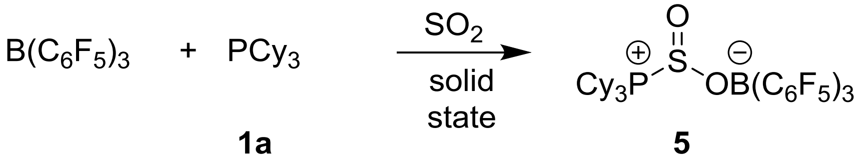

Scheme S8. Reaction of PCy_3 (**1a**) and $B(C_6F_5)_3$ with SO_2 in the solid state: preparation of compound **5**

In a Schlenk flask $B(C_6F_5)_3$ (102.4 mg, 0.2 mmol) and PCy_3 (56.1 mg, 0.2 mmol) were mixed and stirred for 15 min. Then the mixture was exposed to SO_2 (1.5 bar) and stirred for 4 hours. After the reaction time the obtained powder was characterized by NMR experiments in solution and in the solid state.

The obtained NMR data of the obtained powder in solution (dichloromethane-d_2) were consistent to those given for the isolated compound **5** (see below).

4.2) Reaction of PCy_3 (**1a**) and $B(C_6F_5)_3$ with SO_2 in the solid state: isolation of compound **5**

In a Schlenk flask $B(C_6F_5)_3$ (102.4 mg, 0.2 mmol) and PCy_3 (56.1 mg, 0.2 mmol) were mixed and stirred for 15 min. Then the mixture was exposed to SO_2 (1.5 bar) and stirred for 16 hours. After the SO_2 gas was removed in vacuo, the obtained pale yellow solid was crystallized from dichloromethane/pentane and dried in vacuo to give compound **5** (143.9 mg, 0.168 mmol, 84 %). The obtained crystals were suitable for the X-ray crystal structure analysis.

X-ray crystal structure analysis of compound 5: formula $C_{36}H_{33}BF_{15}O_2PS$, M = 856.46, colourless crystal, 0.23 x 0.20 x 0.05 mm, a = 10.9369(2), b = 12.6020(2), c = 15.7611(4) Å, α = 90.384(1), β = 97.992(1), γ = 111.759(2)°, V = 1993.9(1) Å^3, ρ_{calc} = 1.427 gcm^{-3}, μ = 0.223 mm^{-1}, empirical absorption correction (0.950 ≤ T ≤ 0.988), Z = 2, triclinic, space group $P\bar{1}$ (No. 2), λ = 0.71073 Å, T = 223(2) K, ω and φ scans, 18732 reflections collected ($\pm h$, $\pm k$, $\pm l$), 6810 independent (R_{int} = 0.040) and 5755 observed reflections [I>2σ(I)], 505 refined parameters, R = 0.049, wR^2 = 0.117, max. (min.) residual electron density 0.35 (-0.39) e.Å^{-3}, hydrogen atoms were calculated and refined as riding atoms.

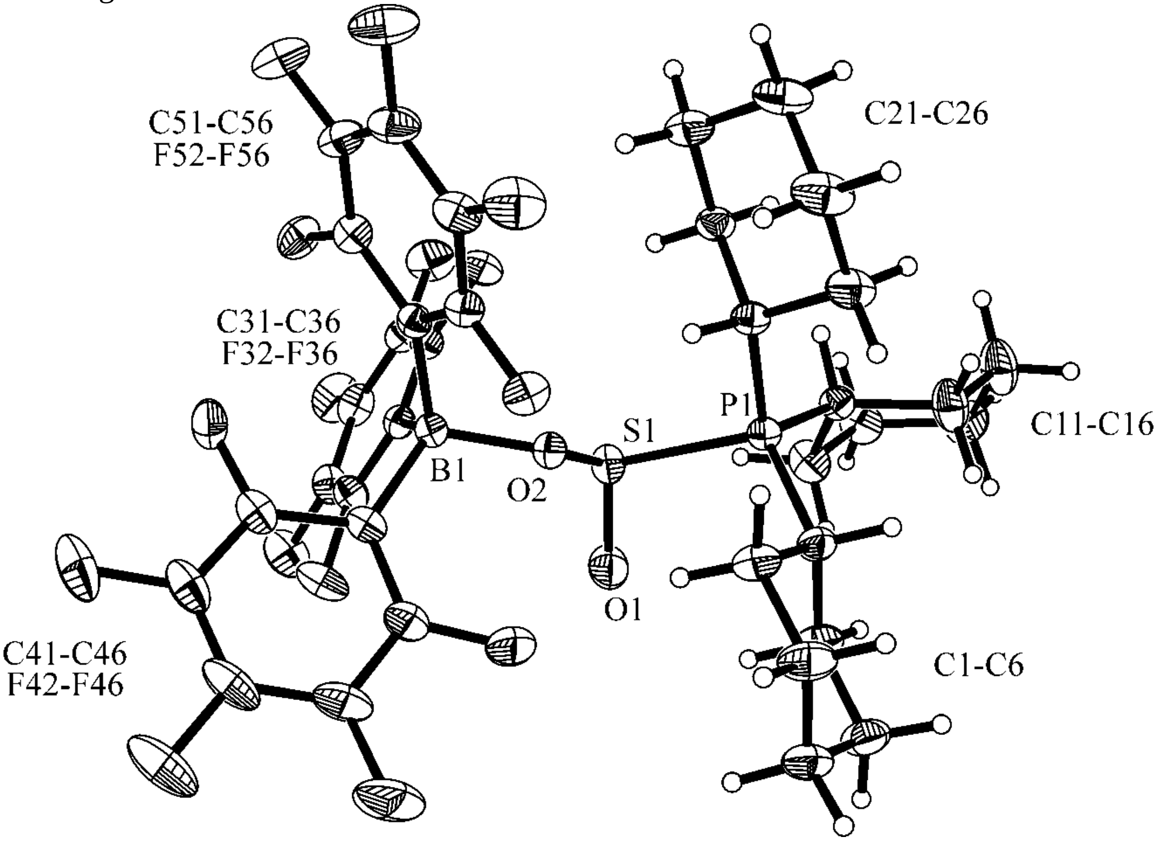

Figure S48. X-ray crystal structure analysis of compound **5**.

^{1}H NMR (500 MHz, 299K, dichloromethane-d$_2$) δ = 2.84 (m, 1H, CH^{Cy}), 2.16, 2.08, 1.89/1.37. 1.86/1.32, 1.78/1.30, 1.64, 1.62 (each br m, each 1H, CH$_2$^{Cy}).
^{13}C{^{1}H} NMR (126 MHz, 299K, dichloromethane-d$_2$) δ = 148.3 (dm, $^{1}J_{FC}$ ~ 240 Hz, C_6F_5), 140.1 (dm, $^{1}J_{FC}$ ~ 250 Hz, C_6F_5), 137.3 (dm, $^{1}J_{FC}$ ~ 245 Hz, C_6F_5), 119.4 (br, i-C_6F_5), 32.2 (d, $^{1}J_{PC}$ = 17.4 Hz, CH^{Cy}), 27.70 (d, $^{2}J_{PC}$ = 2.8 Hz)^{t}, 27.65 (d, $^{2}J_{PC}$ = 2.0 Hz)^{t}, 27.1 (d, J_{PC} = 11.2 Hz), 26.9 (d, J_{PC} = 11.2 Hz), 25.7 (d, J_{PC} = 1.5 Hz)(CH$_2$^{Cy}), [^{t} tentatively assigned]
^{11}B{^{1}H} NMR (160 MHz, 299K, dichloromethane-d$_2$) δ = -0.3 ($\nu_{1/2}$ ~ 250 Hz).
^{19}F NMR (470 MHz, 299K, dichloromethane-d$_2$) δ = -132.3 (m, 2F, *o*-C_6F_5), -159.1 (t, $^{3}J_{FF}$ = 20.3 Hz, 1F, *p*-C_6F_5), -165.5 (m, 2F, *m*-C_6F_5), [$\Delta\delta^{19}F_{m,p}$ = 6.4].
^{31}P{^{1}H} NMR (202 MHz, 299K, dichloromethane-d$_2$) δ = 50.0 ($\nu_{1/2}$ ~ 10 Hz).

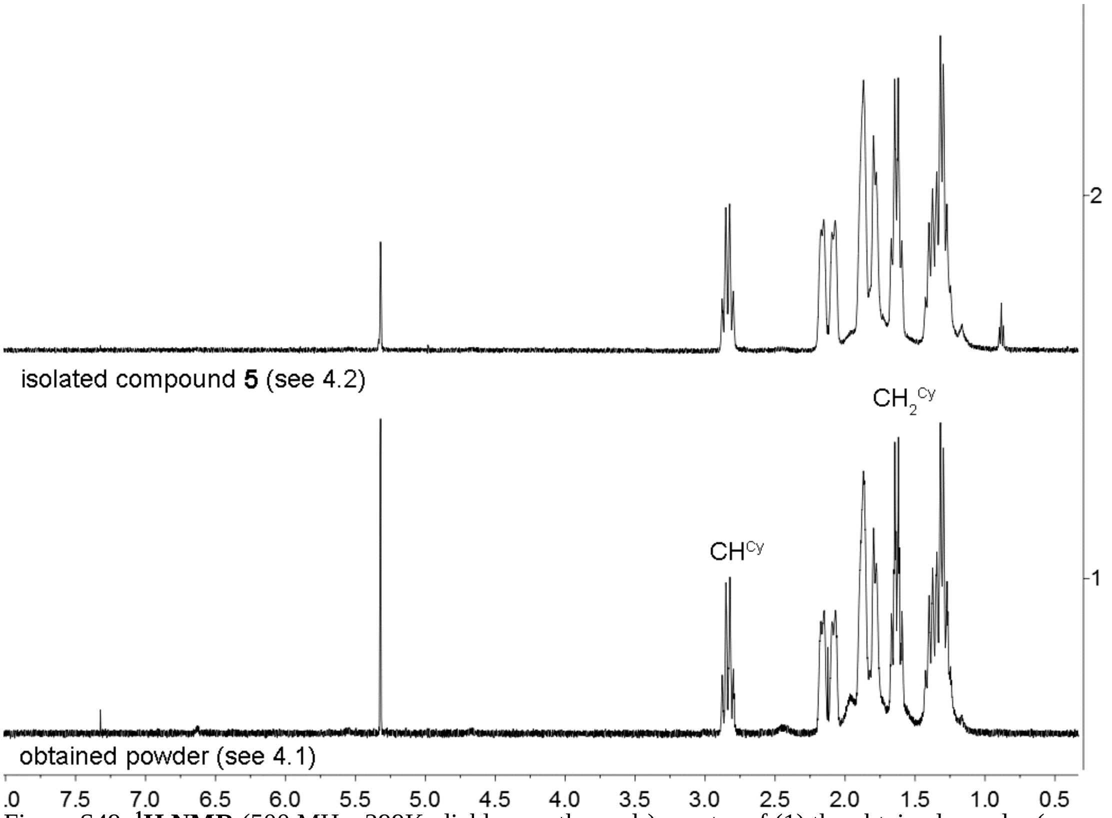

Figure S49. **^{1}H NMR** (500 MHz, 299K, dichloromethane-d$_2$) spectra of (1) the obtained powder (see experiment 4.1) and (2) the isolated compound **5** (see experiment 4.2).

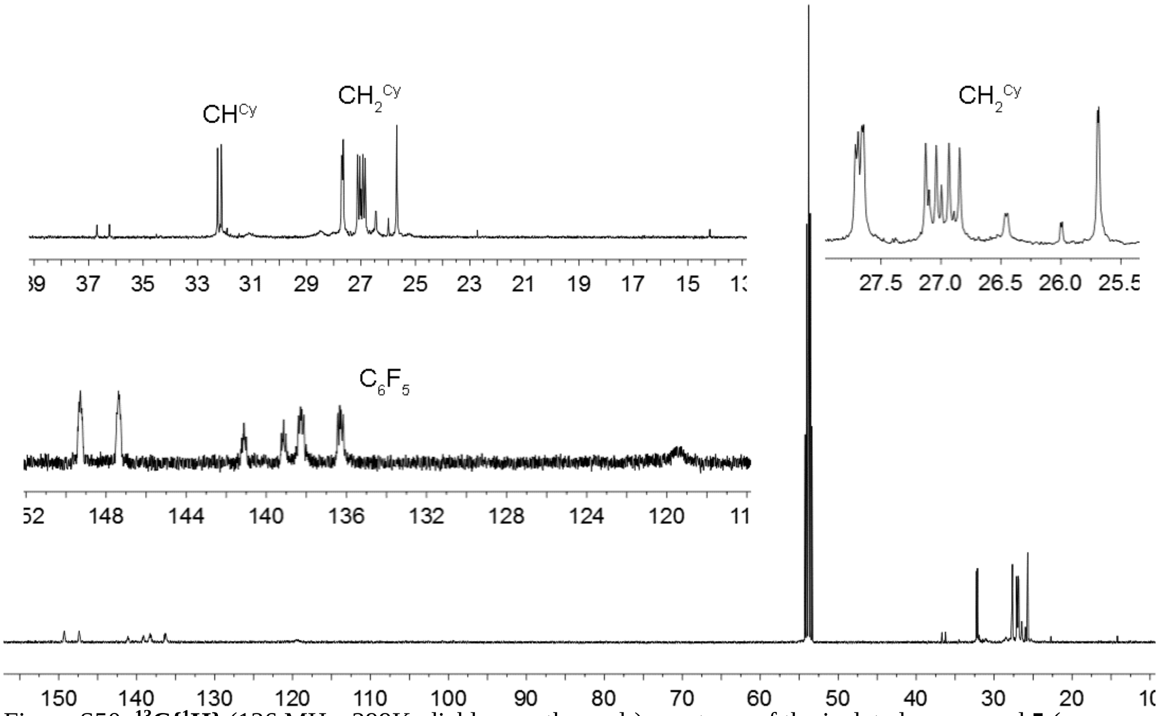

Figure S50. **^{13}C{^{1}H}** (126 MHz, 299K, dichloromethane-d_2) spectrum of the isolated compound **5** (see experiment 4.2).

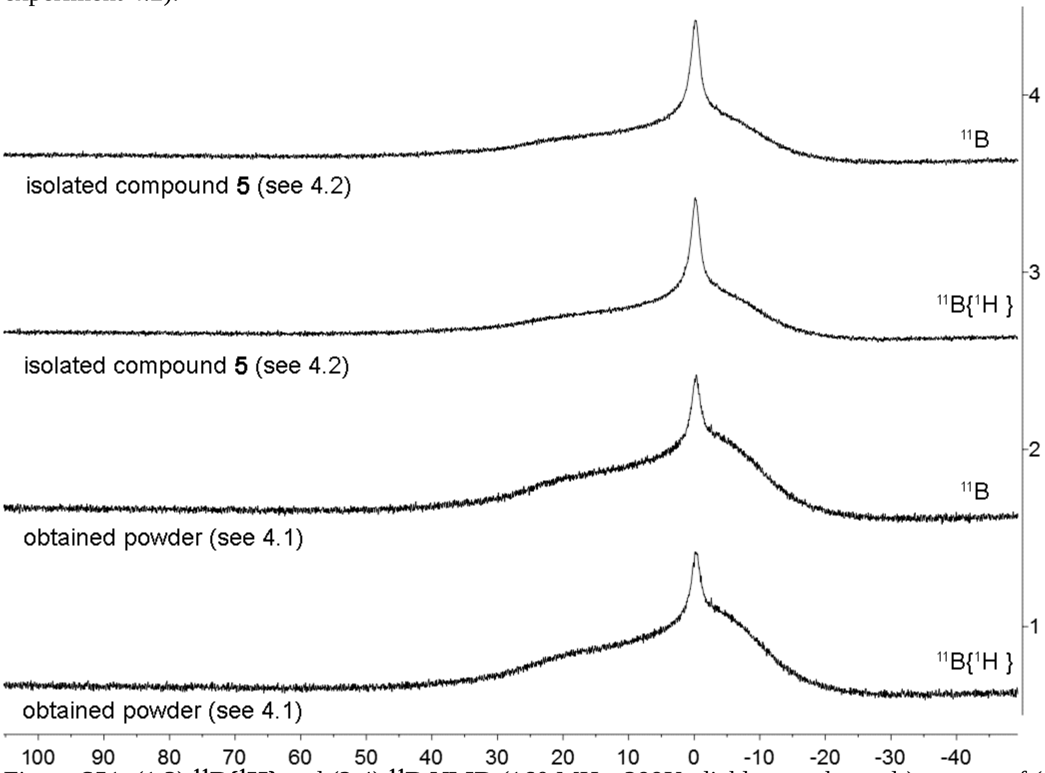

Figure S51. (1,3) **^{11}B{^{1}H}** and (2,4) **^{11}B NMR** (160 MHz, 299K, dichloromethane-d_2) spectra of (1,2) the obtained powder (see experiment 4.1) and (3,4) the isolated compound **5** (see experiment 4.2).

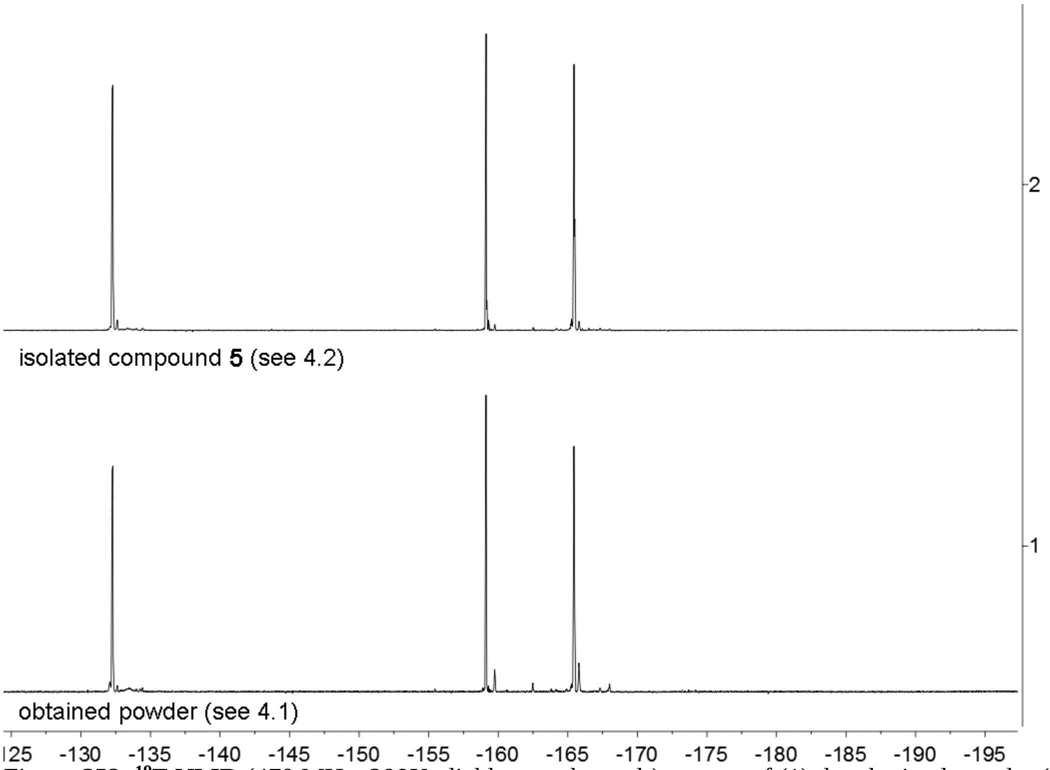

Figure S52. ^{19}**F NMR** (470 MHz, 299K, dichloromethane-d_2) spectra of (1) the obtained powder (see experiment 4.1) and (2) the isolated compound **5** (see experiment 4.2).

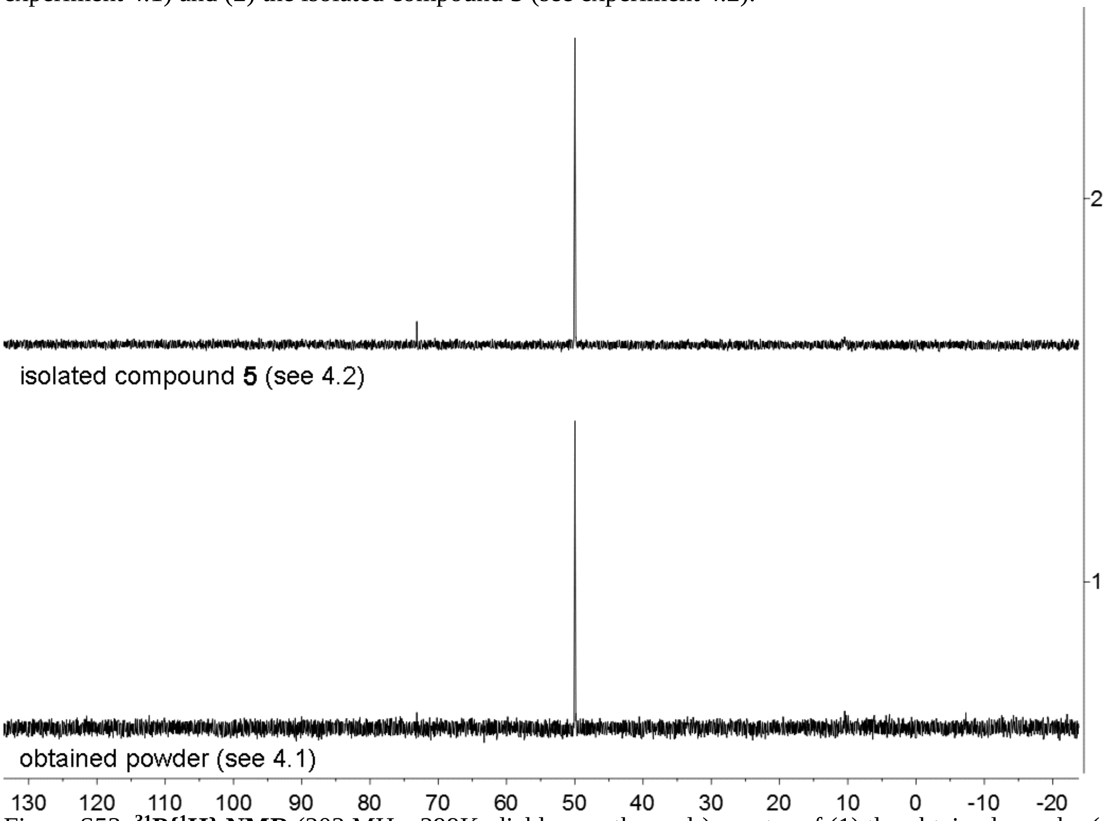

Figure S53. ^{31}**P{^{1}H} NMR** (202 MHz, 299K, dichloromethane-d_2) spectra of (1) the obtained powder (see experiment 4.1) and (2) the isolated compound **5** (see experiment 4.2).

4.3) Reaction of PCy_3 (**1a**) and $B(C_6F_5)_3$ with SO_2 in solution: generation of compound **3a**

A solution of $B(C_6F_5)_3$ (51.2 mg, 0.1 mmol) and PCy_3 (28.0 mg, 0.1 mmol) in CD_2Cl_2 (1 mL) was exposed to SO_2 (1.5 bar) and stirred for 4 hours. Then the mixture was characterized by NMR experiments.
[Comment: the NMR data were consistent to those listed for compound **3a** (see experiment 1.3]

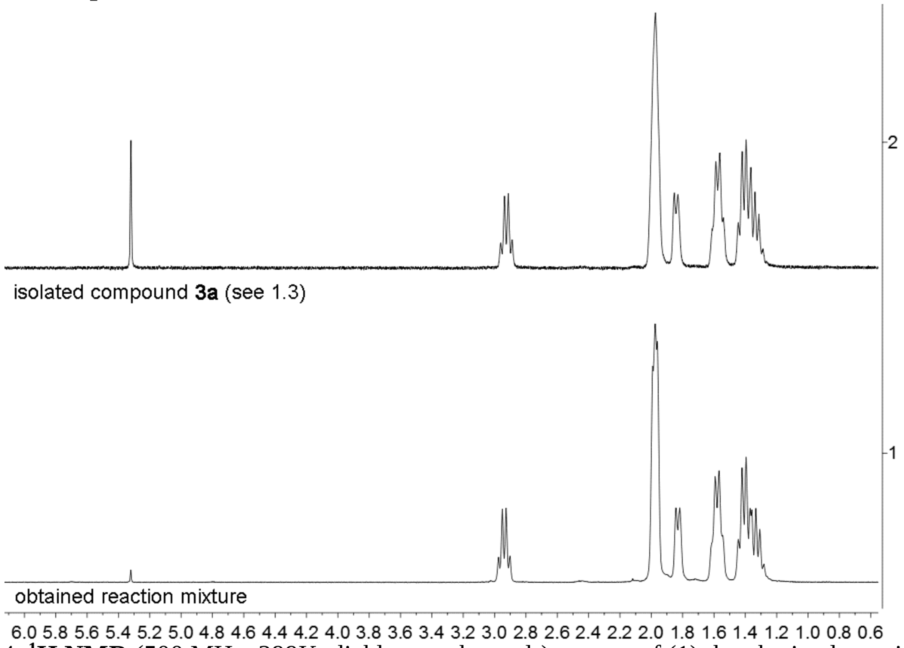

Figure S54. ^{1}**H NMR** (500 MHz, 299K, dichloromethane-d_2) spectra of (1) the obtained reaction mixture and (2) the isolated compound **3a** (see experiment 1.3).

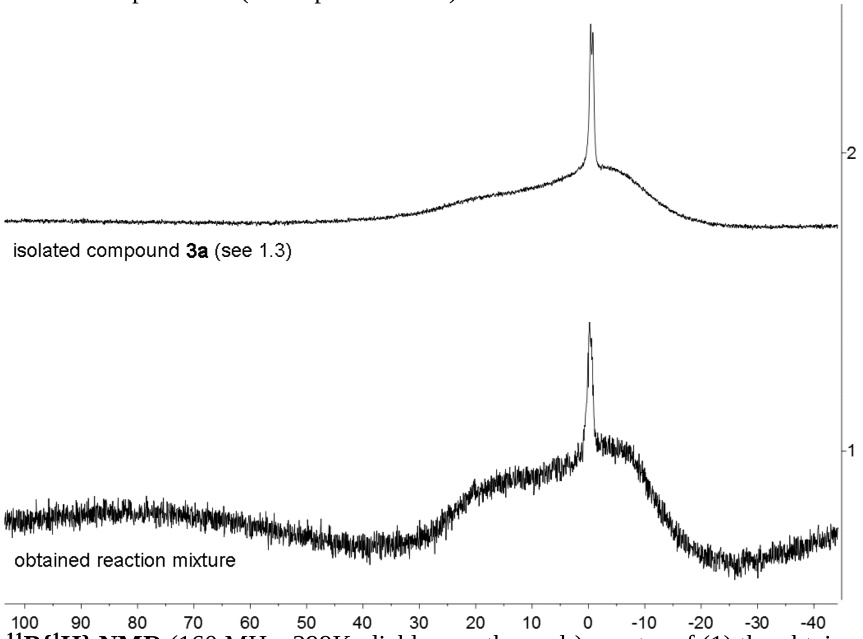

Figure S55. ^{11}**B{^{1}H} NMR** (160 MHz, 299K, dichloromethane-d_2) spectra of (1) the obtained reaction mixture and (2) the isolated compound **3a** (see experiment 1.3).

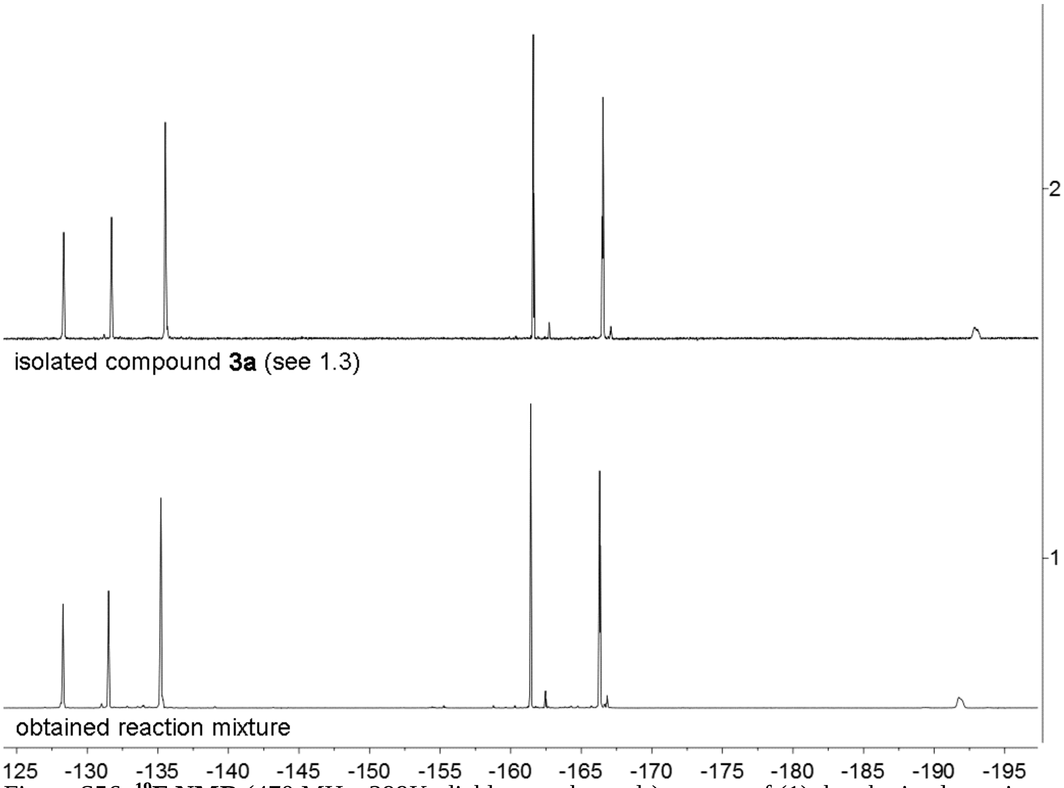

Figure S56. **^{19}F NMR** (470 MHz, 299K, dichloromethane-d_2) spectra of (1) the obtained reaction mixture and (2) the isolated compound **3a** (see experiment 1.3).

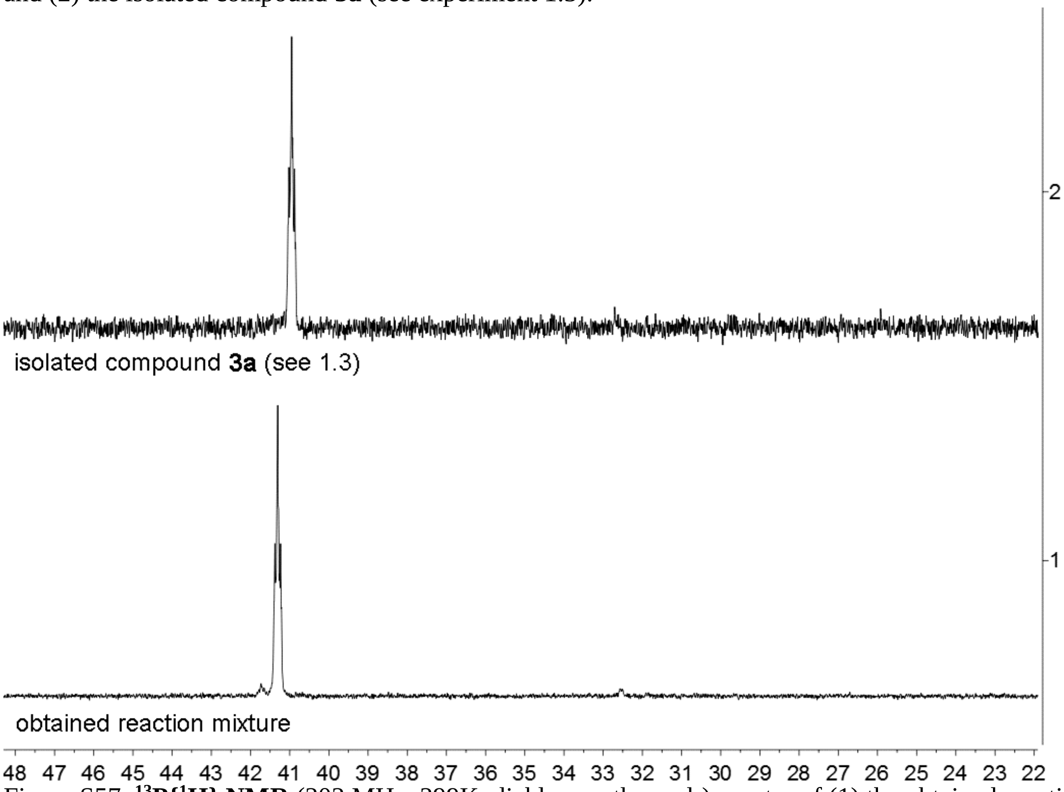

Figure S57. **^{13}P{^{1}H} NMR** (202 MHz, 299K, dichloromethane-d_2) spectra of (1) the obtained reaction mixture and (2) the isolated compound **3a** (see experiment 1.3).

5) Reactions in perfluoromethylcyclohexane solvent

5.1) Reactions of PCy_3 (**1a**) with $B(C_6F_5)_3$ in perfluoromethylcyclohexane in a H_2 atmosphere and control experiment

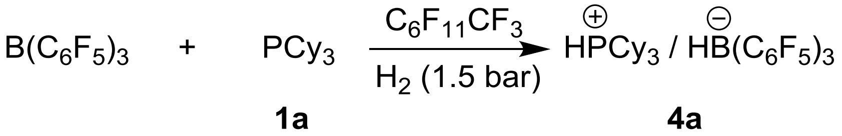

Scheme S9. Reactions of PCy_3 (**1a**) with $B(C_6F_5)_3$ in perfluoromethylcyclohexane in a H_2 atmosphere.

In a Schlenk flask with a magnetic stirring bar, $B(C_6F_5)_3$ (51.2 mg, 0.1 mmol) and PCy_3 (28.0 mg, 0.1 mmol) were suspended in perfluoromethylcyclohexane (2 mL). The reaction mixture was evacuated carefully under -78 °C (dry ice / isopropanol bath) and then stirred under H_2 (1.5 bar) at room temperature for 10 hours. After evaporation of all the volatiles to give a white powder, 15 mg of the powder in dichloromethane-d_2 (1 mL) was determined by NMR and compared with isolated compound **4a** (see experiment 1.2). The NMR shows that ca 60 % of the starting materials converted to **4a**. The white powder was also measured by solid state NMR.

Control experiment: In a Schlenk flask with a magnetic stirring bar, $B(C_6F_5)_3$ (51.2 mg, 0.1 mmol) and PCy_3 (28.0 mg, 0.1 mmol) were suspended in perfluoromethylcyclohexane (2 mL). The mixture was stirred in glovebox for 10 hours. White powder was obtained after evaporation of all the volatiles from this suspension. The white powder from this control experiment was also measured by NMR in solid state.

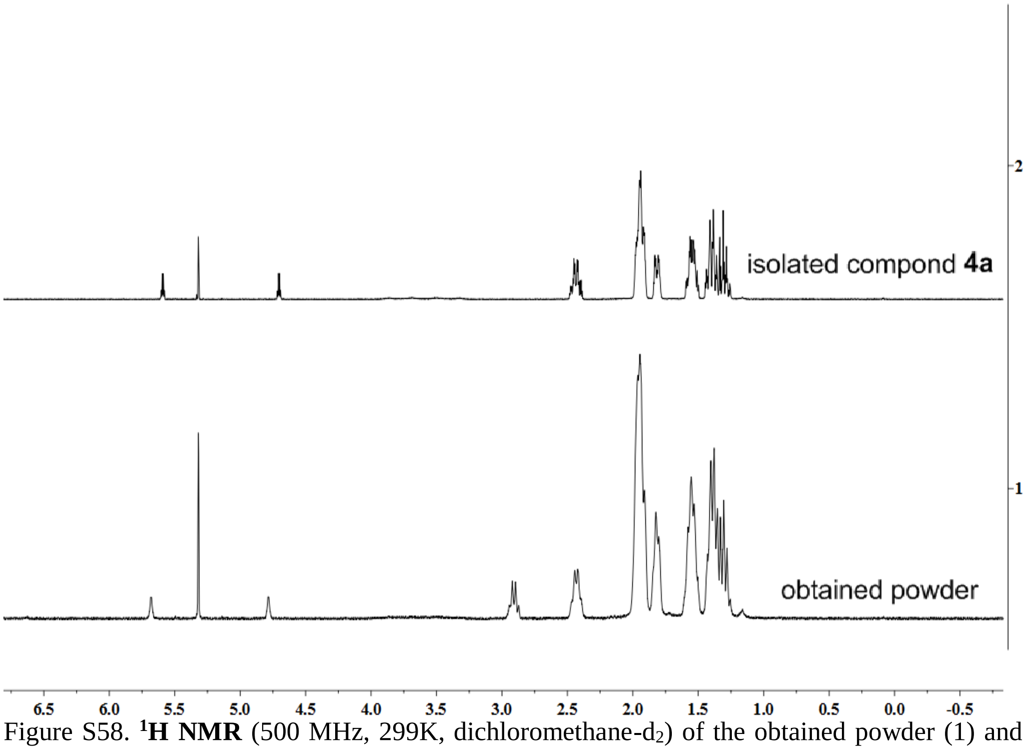

Figure S58. **^{1}H NMR** (500 MHz, 299K, dichloromethane-d_2) of the obtained powder (1) and the isolated compound **4a** (2)

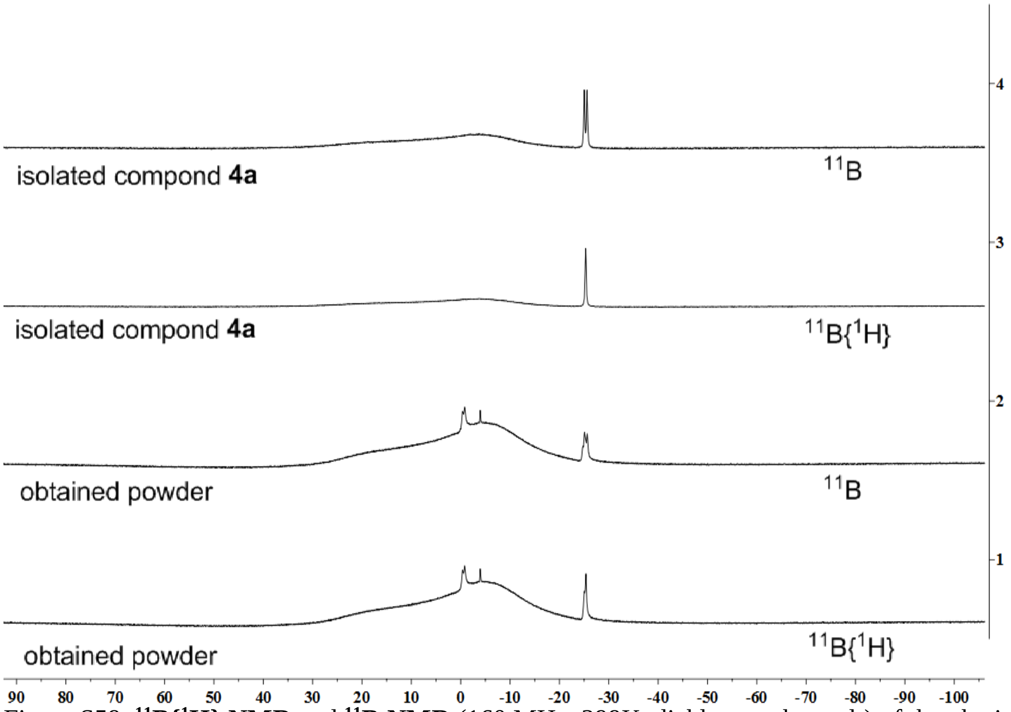

Figure S59. **$^{11}B\{^1H\}$ NMR** and **^{11}B NMR** (160 MHz, 299K, dichloromethane-d_2) of the obtained powder (1, 2) and the isolated compound **4a** (3, 4)

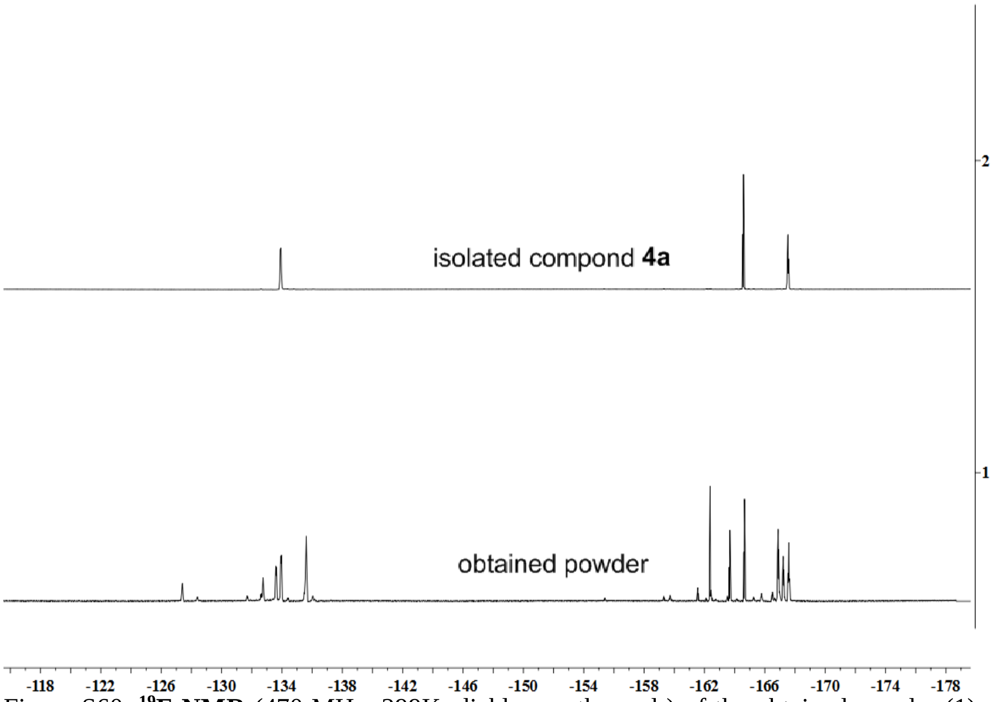

Figure S60. **^{19}F NMR** (470 MHz, 299K, dichloromethane-d_2) of the obtained powder (1) and the isolated compound **4a** (2)

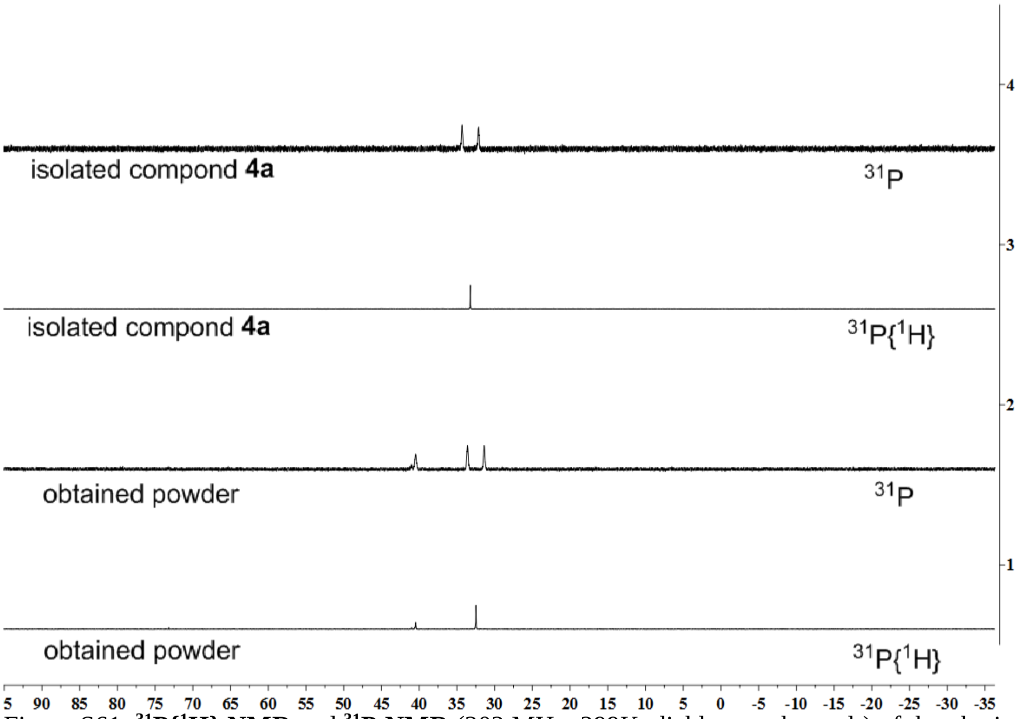

Figure S61. **$^{31}P\{^1H\}$ NMR** and **^{31}P NMR** (202 MHz, 299K, dichloromethane-d_2) of the obtained powder (1, 2) and the isolated compound **4a** (3, 4)

5.2) Reactions of $PhPCy_2$ (**1b**) with $B(C_6F_5)_3$ in perfluoromethylcyclohexane in a H_2 atmosphere

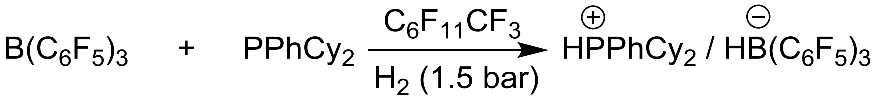

1b **4b**

Scheme S10. Reactions of $PhPCy_2$ (**1b**) with $B(C_6F_5)_3$ in perfluoromethylcyclohexane in a H_2 atmosphere.

In a Schlenk flask with a magnetic stirring bar, $B(C_6F_5)_3$ (51.2 mg, 0.1 mmol) and $PPhCy_2$ (27.4 mg, 0.1 mmol) were suspended in perfluoromethylcyclohexane (2 mL). The reaction mixture was evacuated carefully under -78 ^oC (dry ice / isopropanol bath) and then stirred under H_2 (1.5 bar) at room temperature for 10 hours. After evaporation of all the volatiles to give a white powder, 15 mg of the powder in dichloromethane-d_2 (1 mL) was determined by NMR and compared with isolated compound **4b** (see experiment 2.1). The NMR shows that the starting materials were almost fully converted to **4b** just with little impurities (ca. 95% conversion).

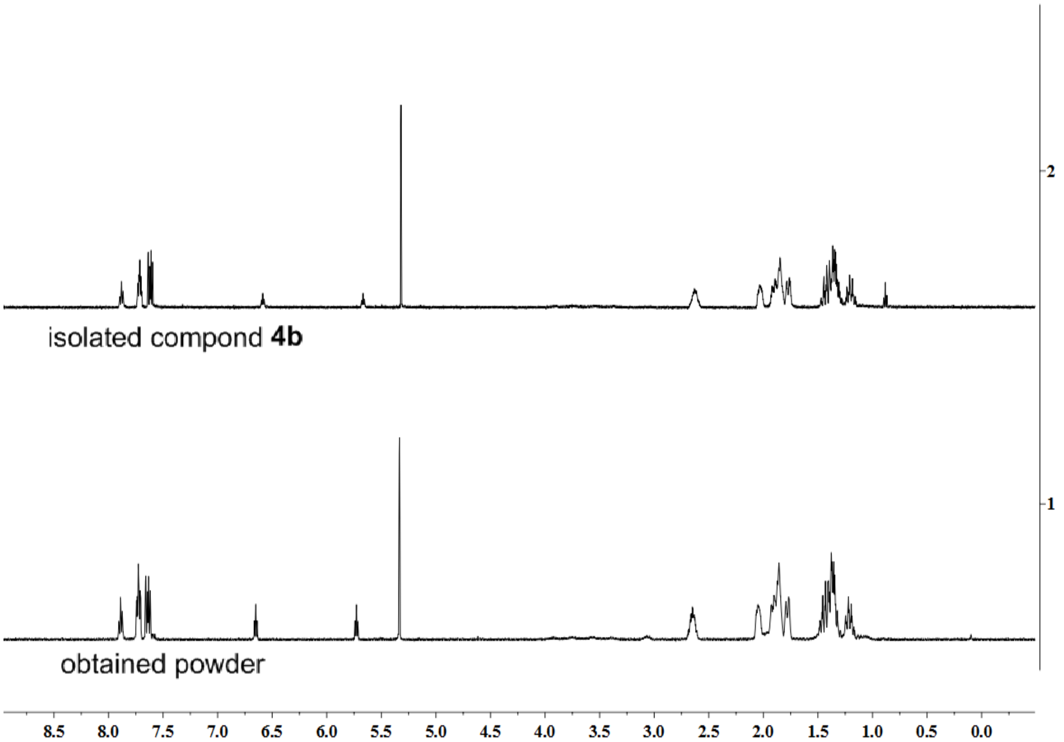

Figure S62. ^{1}**H NMR** (500 MHz, 299K, dichloromethane-d_2) of the obtained powder (1) and the isolated compound **4b** (2)

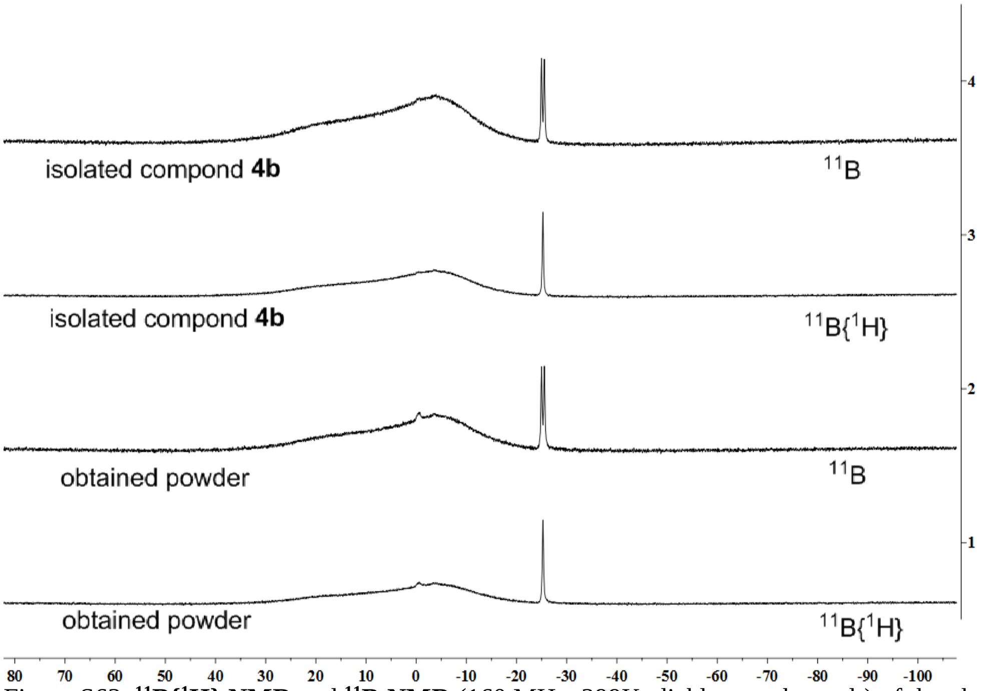

Figure S63. ^{11}**B{**^{1}**H} NMR** and ^{11}**B NMR** (160 MHz, 299K, dichloromethane-d_2) of the obtained powder (1, 2) and the isolated compound **4b** (3, 4)

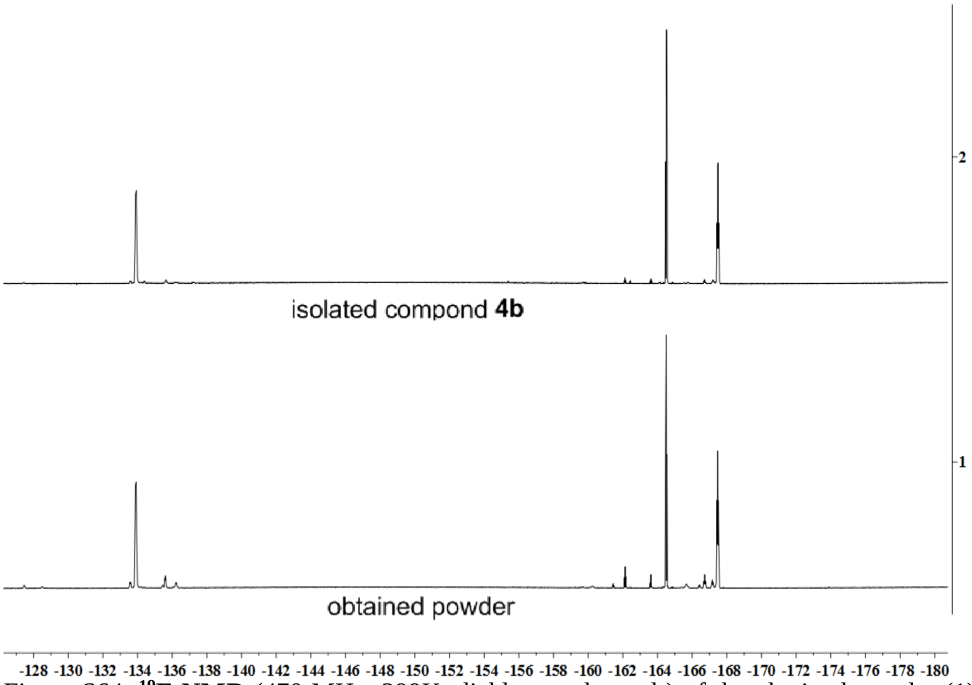

Figure S64. **^{19}F NMR** (470 MHz, 299K, dichloromethane-d_2) of the obtained powder (1) and the isolated compound **4b** (2)

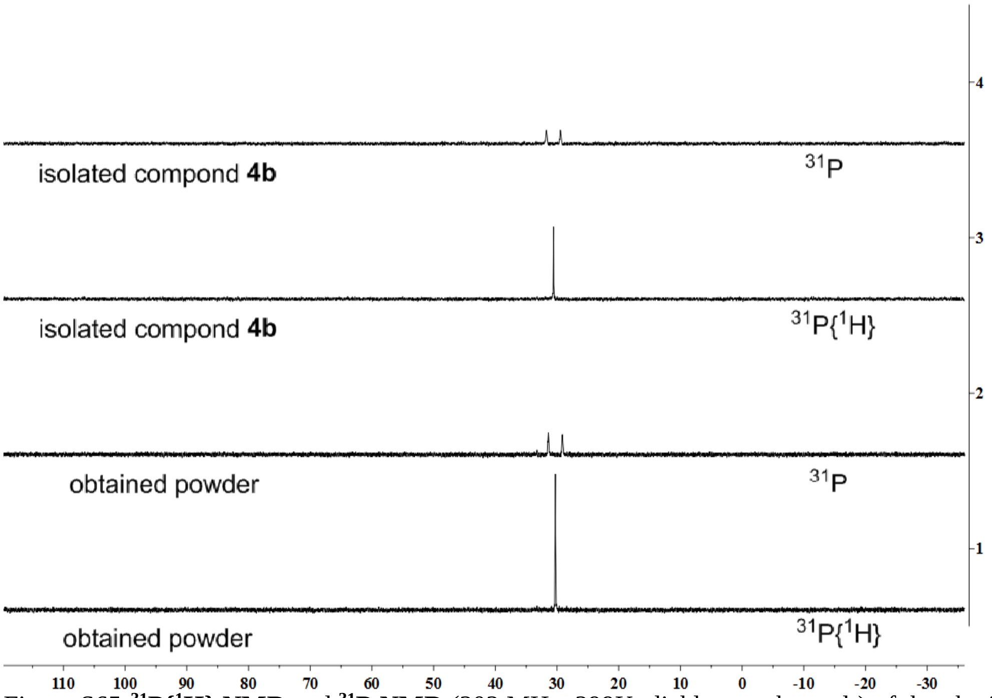

Figure S65. **^{31}P{^{1}H} NMR** and **^{31}P NMR** (202 MHz, 299K, dichloromethane-d_2) of the obtained powder (1, 2) and the isolated compound **4b** (3, 4)

5.3) Reactions of PCy_3 **(1c)** with $B(C_6F_5)_3$ in perfluoromethylcyclohexane in a H_2 atmosphere

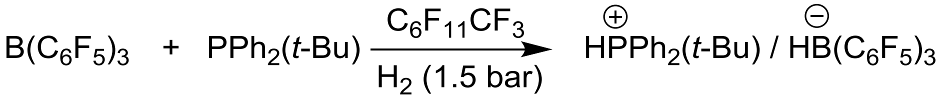

1c **4c**

Scheme S11. Reactions of PCy_3 **(1c)** with $B(C_6F_5)_3$ in perfluoromethylcyclohexane in a H_2 atmosphere

In a Schlenk flask with a magnetic stirring bar, $B(C_6F_5)_3$ (51.2 mg, 0.1 mmol) and PPh_2(*t*-Bu) (24.2 mg, 0.1 mmol) were suspended in perfluoromethylcyclohexane (2 mL). The reaction mixture was evacuated carefully under -78 °C (dry ice / isopropanol bath) and then stirred under H_2 (1.5 bar) at room temperature for 10 hours. After evaporation of all the volatiles to give a white powder, 15 mg of the powder in dichloromethane-d_2 (1 mL) was determined by NMR and compared with isolated compound **4c** (see experiment 3.1). The NMR shows that the starting materials were almost fully converted to **4c** just with little impurities (ca. 98% conversion).

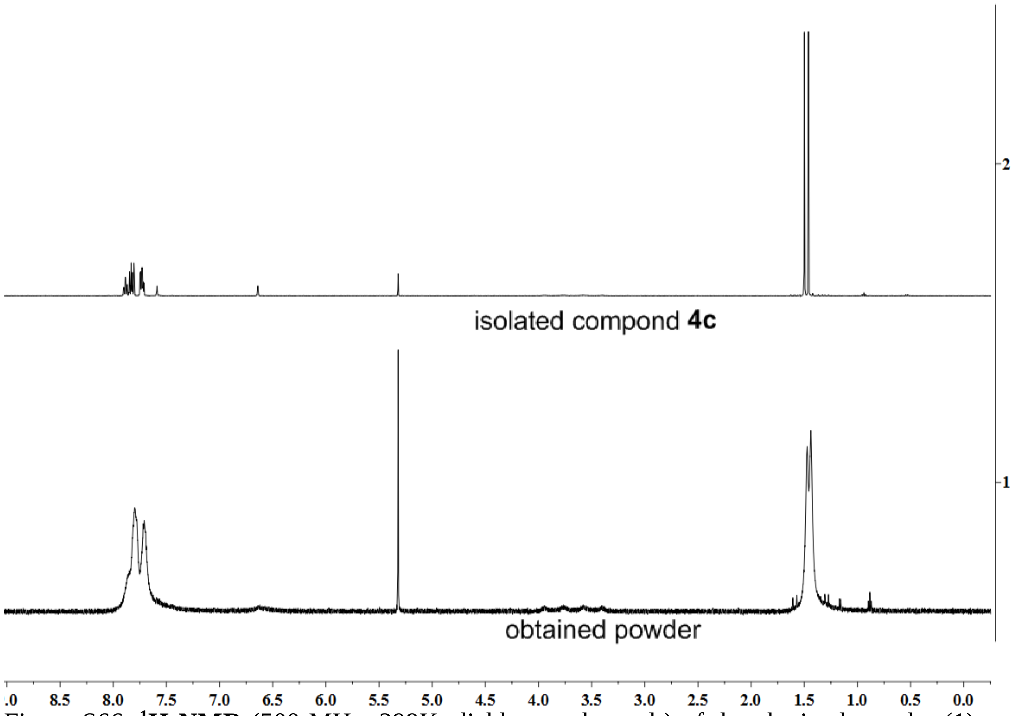

Figure S66. ^{1}**H NMR** (500 MHz, 299K, dichloromethane-d_2) of the obtained powder (1) and the isolated compound **4c** (2)

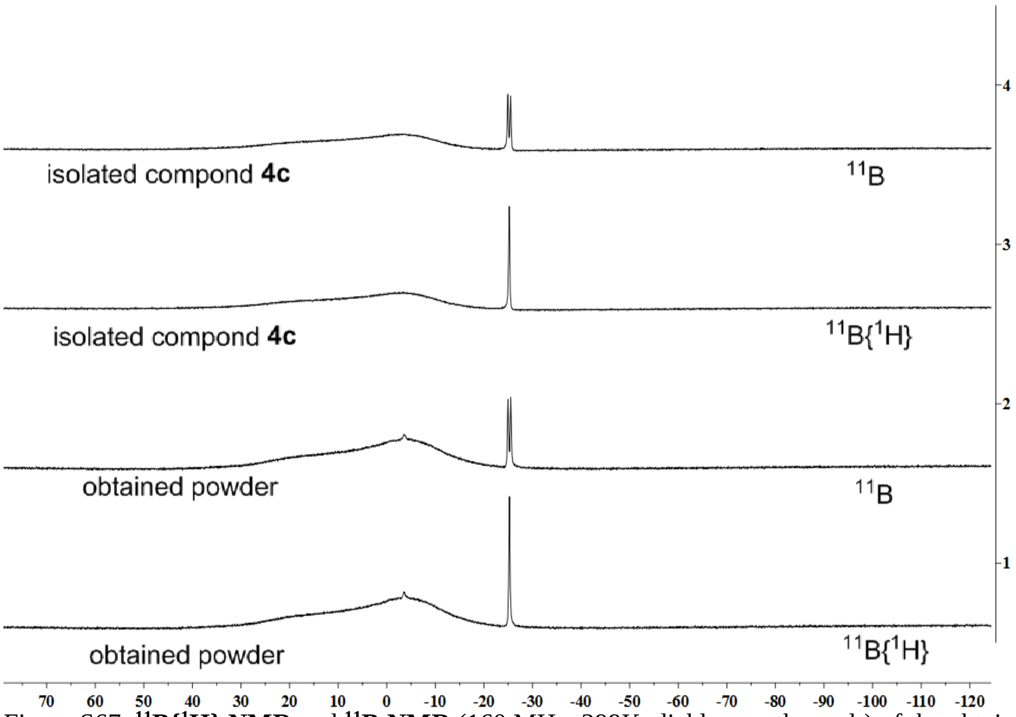

Figure S67. **$^{11}B\{^1H\}$ NMR** and **^{11}B NMR** (160 MHz, 299K, dichloromethane-d_2) of the obtained powder (1, 2) and the isolated compound **4c** (3, 4)

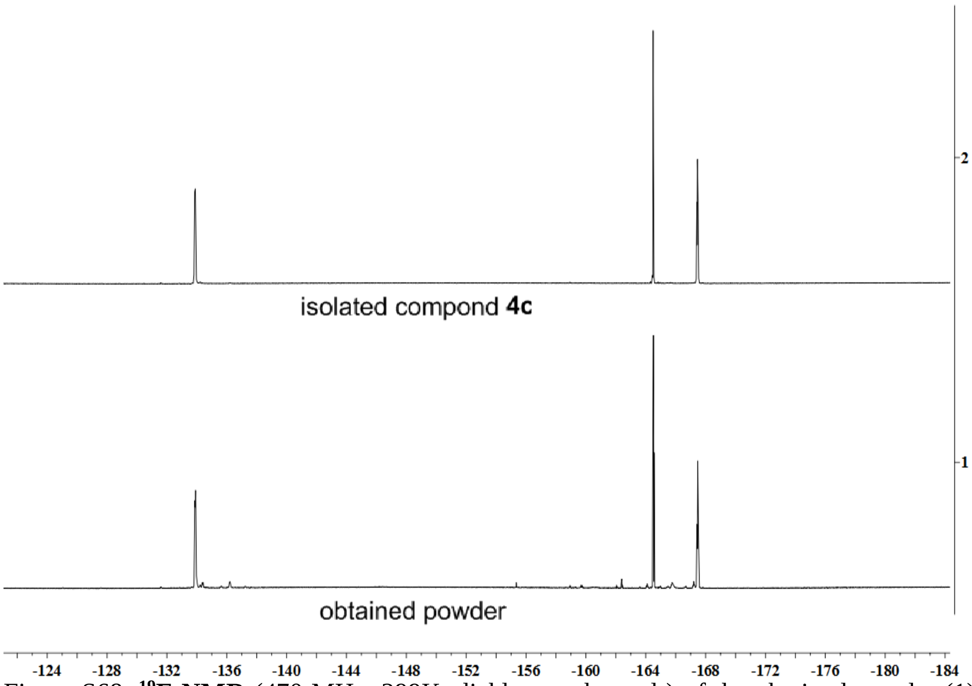

Figure S68. **^{19}F NMR** (470 MHz, 299K, dichloromethane-d_2) of the obtained powder (1) and the isolated compound **4c** (2)

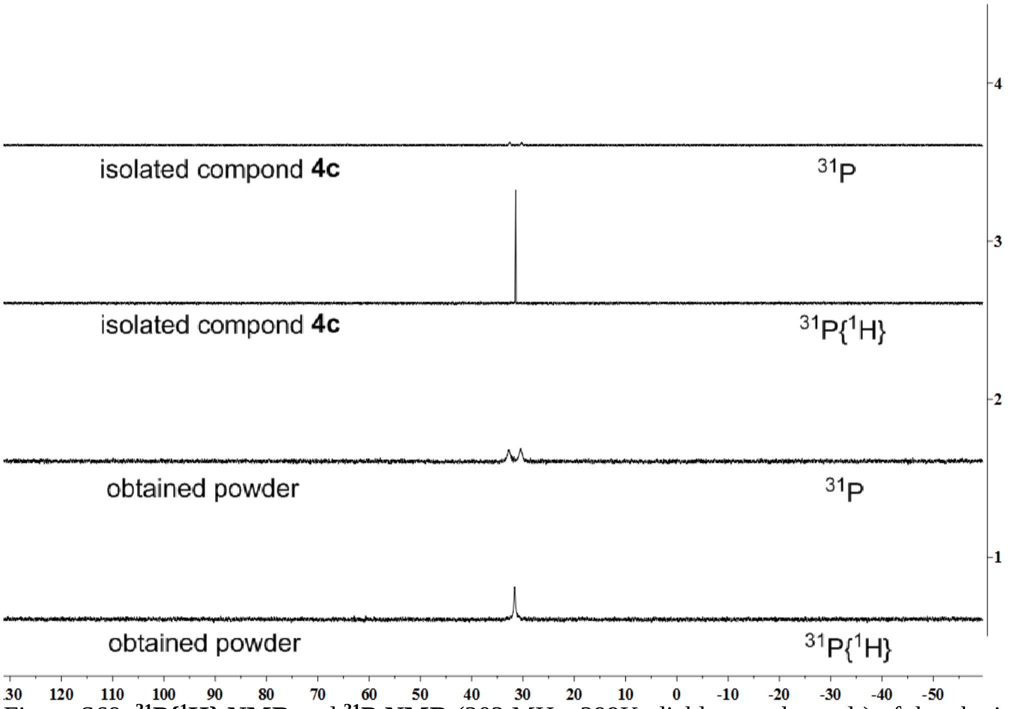

Figure S69. **$^{31}P\{^1H\}$ NMR** and **^{31}P NMR** (202 MHz, 299K, dichloromethane-d_2) of the obtained powder (1, 2) and the isolated compound **4c** (3, 4)

6) Reduction of an imine by compound **4a**

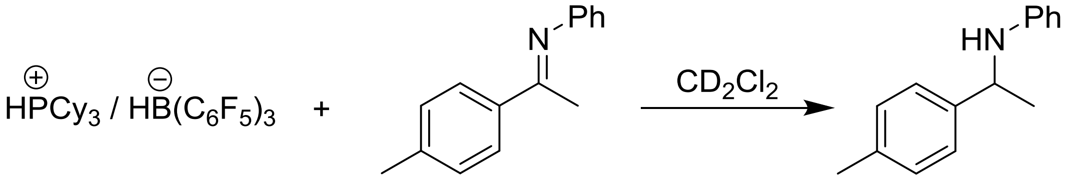

4a **6** **7**

Scheme S12. Reduction of an imine by compound **4a**.

Compound **4a** (23.8 mg, 0.03 mmol) and imine **6** (6.3 mg, 0.03 mmol) were mixed in dichloromethane-d_2 (1 mL). The solution was heated in a sealed *J*-Young tube at 70 ^oC for 24 hours and then measured by NMR. The NMR analysis shows that the conversion of the reaction is ca. 86%.

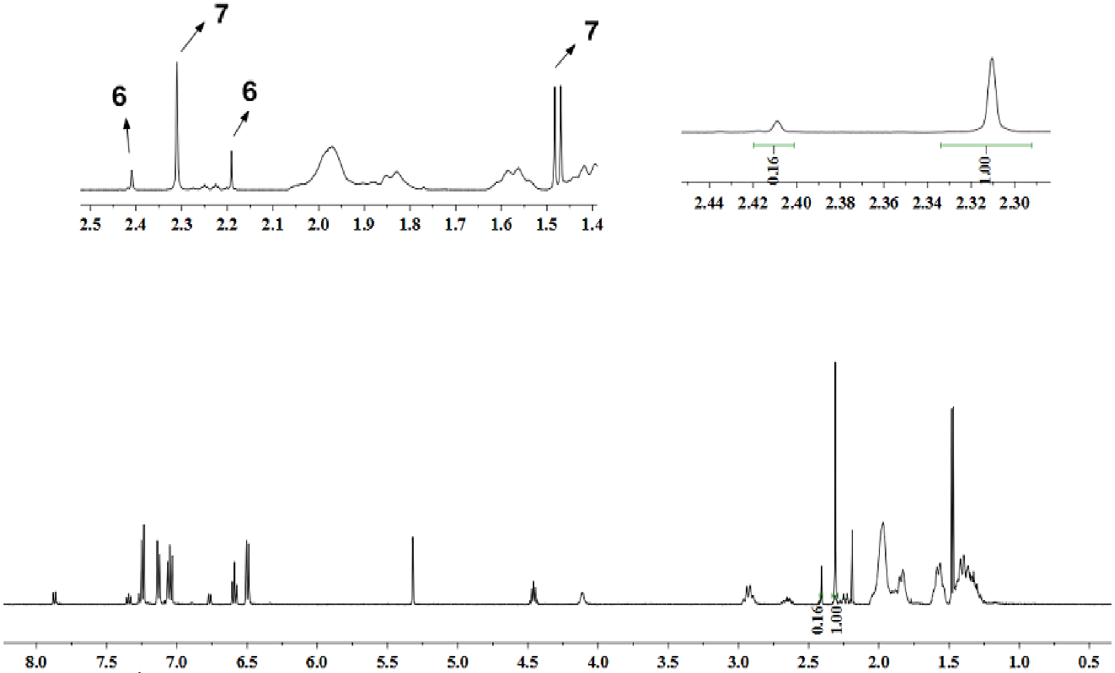

Figure S70. ^{1}**H NMR** (500 MHz, 299K, dichloromethane-d_2) of the reaction mixture

Supplementary Information (Part 2. Solid-State NMR)

Melanie Brinkkötter[b], Thomas Wiegand[c], Anna-Lena Wübker[b], Hellmut Eckert[b,d,*]

[b] Institut für Physikalische Chemie and Graduate School of Chemistry, Westfälische Wilhelms-Universität Münster, Corrensstraße 30, 48149 Münster, Germany (solid state NMR spectroscopy)

[c] Laboratorium für Physikalische Chemie, ETH Zürich, Vladimir-Prelog-Weg 2, 8093 Zürich, Switzerland (high-field, fast rotation ^{1}H MAS-NMR)

[d] Institute of Physics in Sao Carlos, University of Sao Paulo, CEP 369, Sao Carlos SP 13566-590, Brazil (solid-state NMR spectroscopy).

Corresponding line shape simulations for the ^{11}B{^{1}H} MAS and ^{31}P{^{1}H} CPMAS NMR spectra of the hydrogenation sample and the control mixture sample (from experiment 1.1) are shown in Figure S71. Solid-state NMR characterization was carried out also for the analogous reaction in suspension (hydrogenation sample and control mixture sample: see experiment 5.1). Both reaction types yield the same reaction products (Figure S72). In suspension, the investigated product shows a higher amount of substitution product in proportion to the hydrogenation product.
The ^{1}H{^{11}B} REAPDOR experiment in Figure S73 confirms the B-H bond near 4 ppm which was also proven by ^{1}H MAS and ^{11}B{^{1}H} HETCOR. ^{11}B{^{31}P} REDOR and ^{31}P{^{11}B} CP-REAPDOR experiments (Figure S74 and Figure S75, respectively) show an insignificant dephasing effect. REDOR reveals a B-P distance of at least 5.5 Å. These experiments confirm the presence of isolated ions which was already shown by comparison of ^{1}H MAS NMR spectra and DFT calculations.

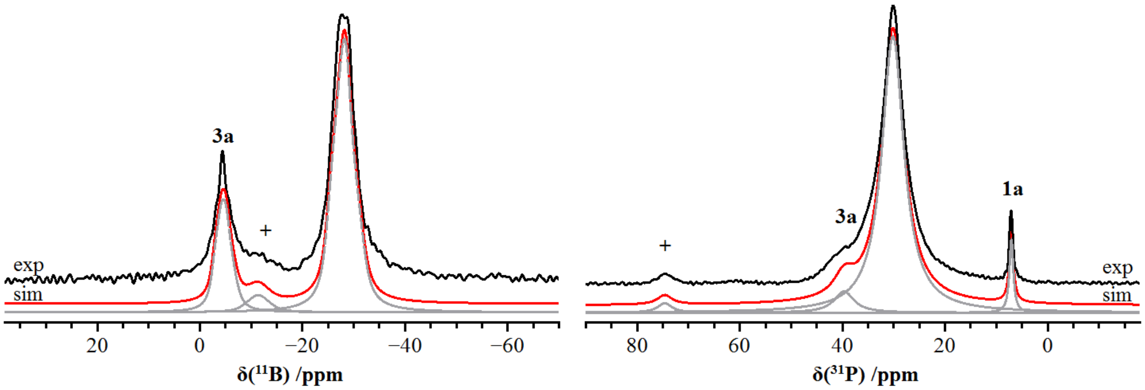

Figure S71. ^{11}B{^{1}H} MAS (left, top) and ^{31}P{^{1}H} CPMAS NMR spectra (right, top) of the hydrogenation sample (from experiment 1.1) measured at 7.05 T with a MAS frequency of 12.0 kHz. Line shape simulations (lower traces) result in δ_{iso}(^{11}B) = -24.9 ppm, C_Q = 0.99 MHz, η_Q = 0.08 and δ_{iso}(^{31}P) = 30.1 ppm. A minor unidentified side product is labelled by +.

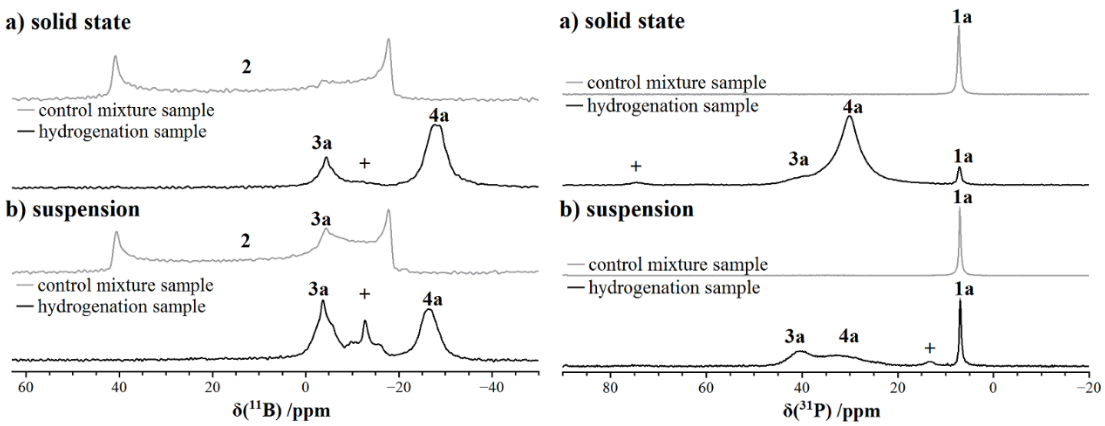

Figure S72. $^{11}B\{^{1}H\}$ MAS (left) and $^{31}P\{^{1}H\}$ CPMAS NMR spectra (right) of the $B(C_6F_5)_3/PCy_3$ control mixture sample (grey trace) and the hydrogenation sample (black trace, the reaction with H_2). The upper traces show the spectra for the reaction in the solid state, the lower ones belong to the reaction in suspension. A minor unidentified compound is labelled by +. (solid state see experiment 1.1, suspension in perfluoromethylcyclohexane see experiment 5.1).

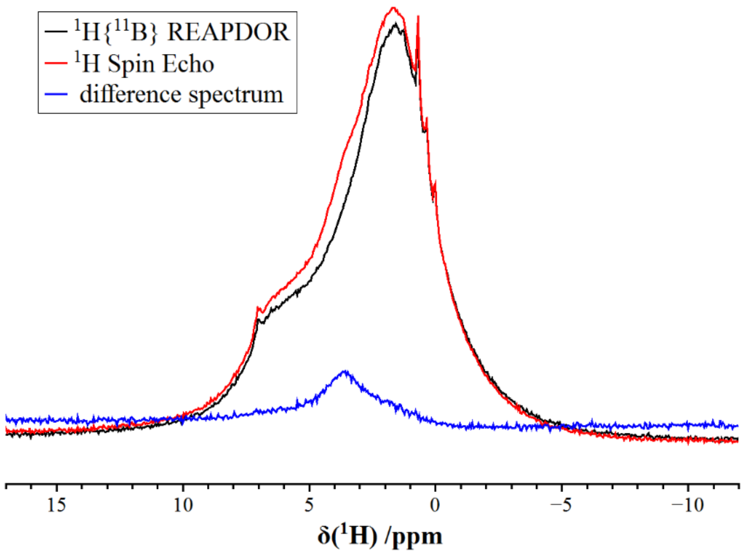

Figure S73. $^{1}H\{^{11}B\}$ REAPDOR spectrum (black) and ^{1}H Spin Echo (red) of the hydrogenation sample (from experiment 1.1) measured at 11.74 T with a MAS frequency of 29.8 kHz. The difference spectrum (blue) reveals the boron bonded hydrogen species at $\delta_{iso}(^{1}H)$ = 3.7 ppm.

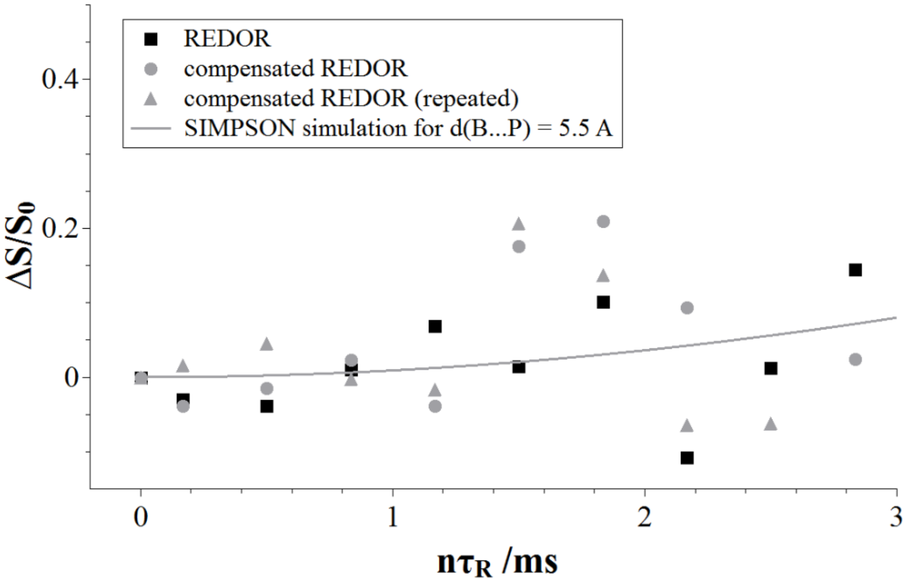

Figure S74. $^{11}B\{^{31}P\}$ REDOR of the hydrogenation sample (from experiment 1.1) measured at 7.05 T with a MAS frequency of 12.0 kHz and SIMPSON simulation for a boron-phosphorus distance of 5.5 Å.

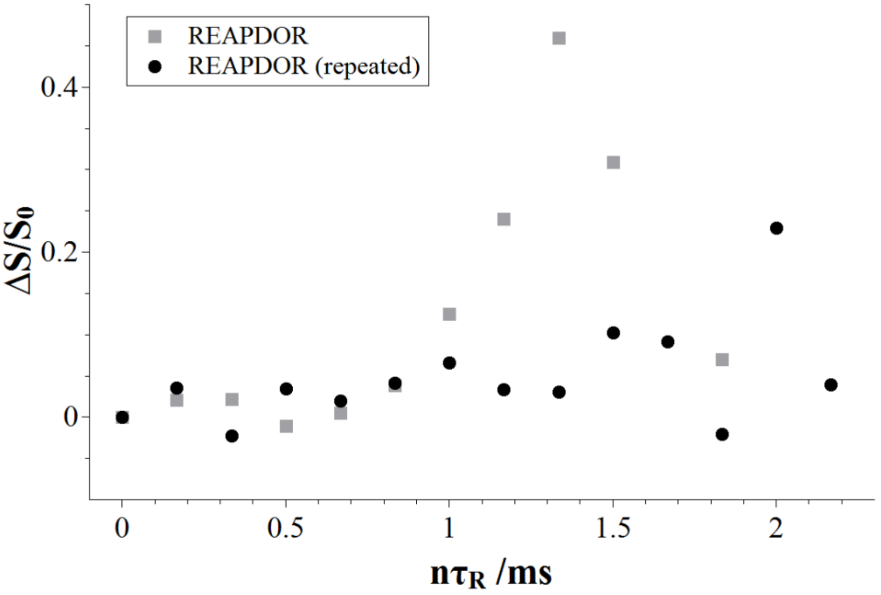

Figure S75. $^{31}P\{^{11}B\}$ CP-REAPDOR of the hydrogenation sample (from experiment 1.1) measured at 7.05 T with a MAS frequency of 12.0 kHz.

The substitution product (compound **3a** from experiment 1.3) and the solid-state SO_2 adduct (sample from experiment 4.1) were also characterized by solid-state NMR. Figures S76 to S79 summarize the solid state NMR characterization of compound **3a**, whereas Figures S80-84 show the characterization of the SO_2 adduct sample from experiment 4.1**.**

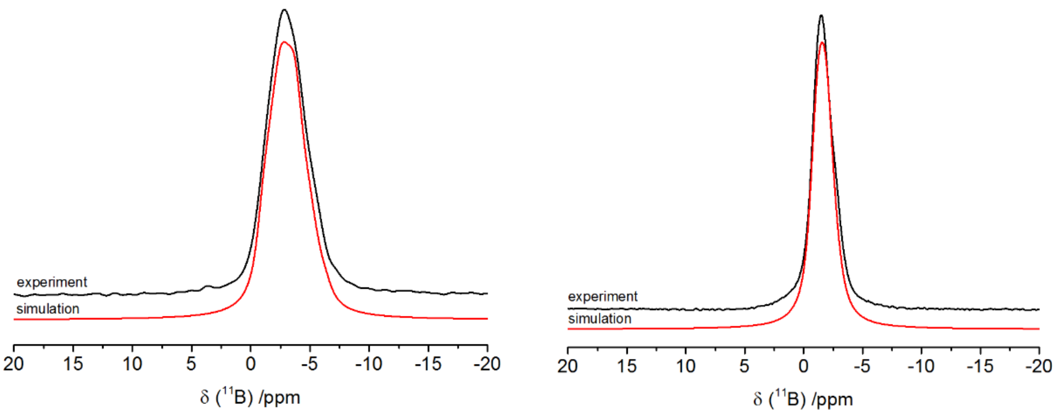

Figure S76. $^{11}B\{^1H\}$ MAS NMR spectrum (top) of the substitution product (compound **3a** from experiment 1.3) measured at 7.05 T (left) and at 11.7 T (right) with a MAS frequency of 12.0 kHz. Line shape simulation (lower traces) results in $\delta_{iso}(^{11}B)$ = -0.6 ppm, C_Q = 0.93 MHz and η_Q = 0.54 and $\delta_{iso}(^{11}B)$ = -0.5 ppm, C_Q = 1.03 MHz and η_Q = 0.47, respectively.

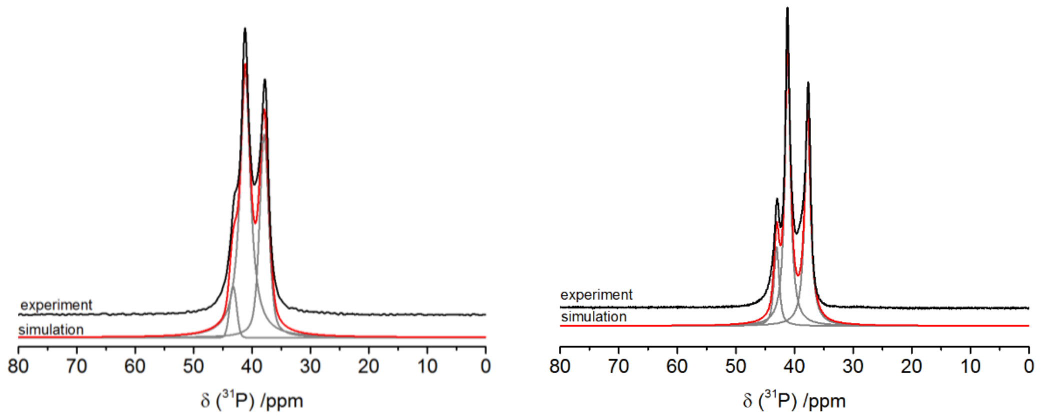

Figure S77. $^{31}P\{^1H\}$ CPMAS NMR spectrum (top) of the substitution product (compound **3a** from experiment 1.3) measured at 7.05 T (left) with a MAS frequency of 12.0 kHz. Line shape simulation (lower traces) was done by using three signal components at $\delta_{iso}(^{31}P)$ = 43.3 ppm, 41.2 ppm and 38.0 ppm. Three phosphorus species were also verified by a $^{31}P\{^1H\}$ CPMAS NMR spectrum at 11.74 T (right).

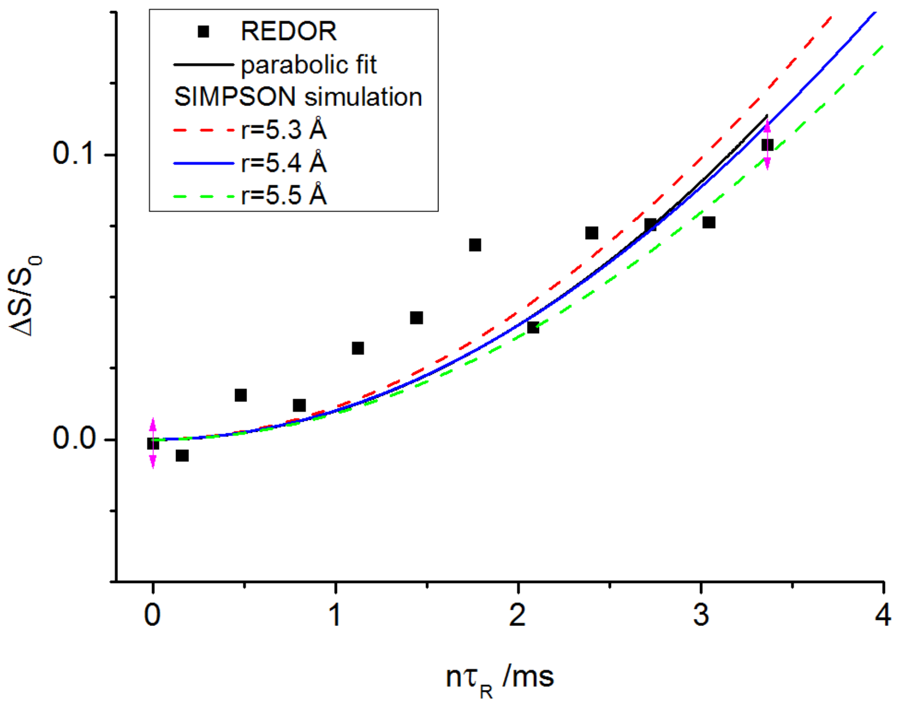

Figure S78. $^{11}B\{^{31}P\}$ REDOR data of the substitution product (compound **3a** from experiment 1.3) measured at 7.05 T with a MAS frequency of 12.5 kHz. The experimental data are compared with SIMPSON simulations for different assumed P—B distances. The simulation most consistent with the experimental data suggests a boron-phosphorus distance of 5.4 Å. Included is also a parabolic fit, following the approach[4] resulting in a second moment value of M_2 = 0.74•10^{-5} rad^2/s^2 from the parabolic fit (black curve). This approach yields a boron-phosphorus distance of 5.4 Å for an assumed two-spin interaction.

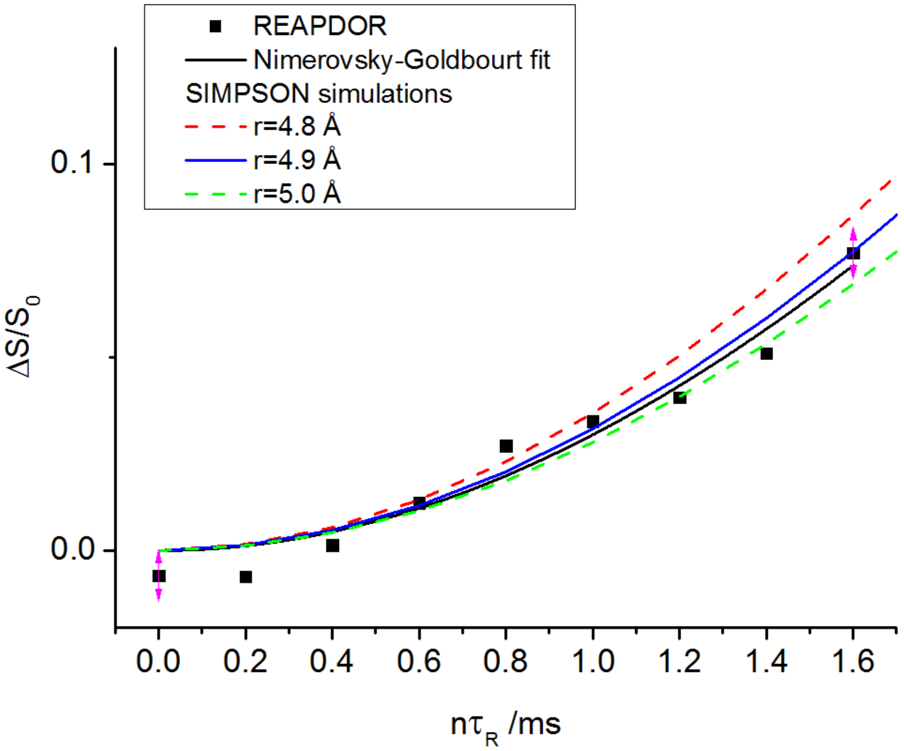

Figure S79. $^{31}P\{^{11}B\}$ CP-REAPDOR of the substitution product (compound **3a** from experiment 1.3) measured at 7.05 T with a MAS frequency of 10.0 kHz. Included are SIMPSON simulation for different assumed B--P distances. The simulation most consistent with the experimental data is obtained for a boron-phosphorus distance of 5.0 Å and the fit to the analytical expression[5] given in results in 4.9 Å.

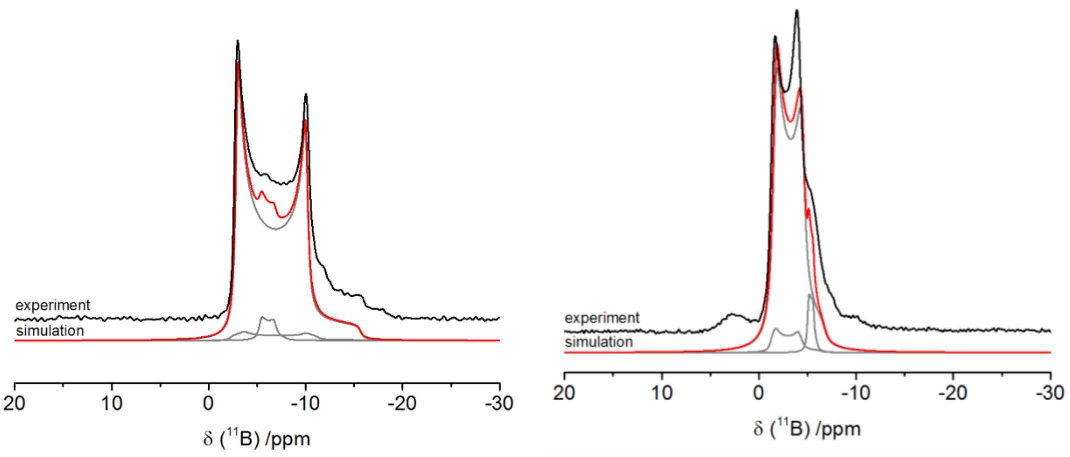

Figure S80. $^{11}B\{^1H\}$ MAS NMR spectrum (top) of the SO_2 adduct sample (sample from experiment 4.1) measured at 7.05 T (left) and 11.7 T (right) with a MAS frequency of 12.0 kHz. Line shape simulation (lower traces) results in $\delta_{iso}(^{11}B)$ = -0.6 ppm, C_Q = 1.54 MHz and η_Q = 0.15. The spectrum shows a weak overlapping component arising from a second and a small third species which could be resolved by $^{11}B\{^1H\}$ MQMAS.

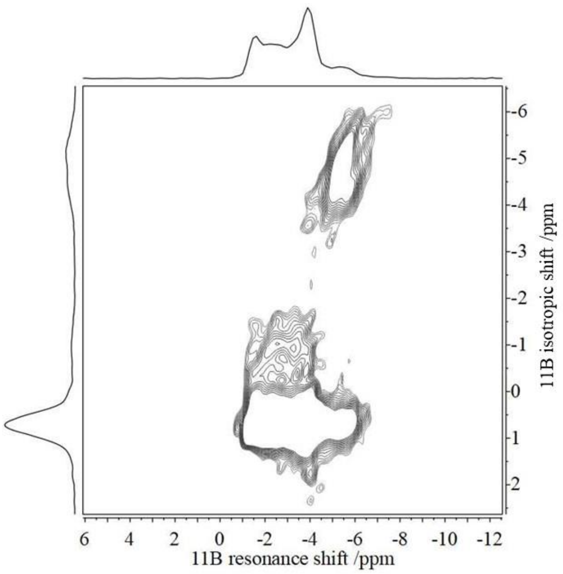

Figure S81. $^{11}B\{^1H\}$ MQMAS spectrum of the SO_2 adduct sample (sample from experiment 4.1) measured at 11.7 T with a MAS frequency of 12.0 kHz. The main species is located at an isotropic chemical shift of $\delta_{iso}(^{11}B)$ = -0.6 ppm and has a second order quadrupolar effect of 1.61 MHz which agrees with C_Q = 1.54 MHz and η_Q = 0.15 from the $^{11}B\{^1H\}$ MAS spectrum. All results were used for line shape simulation of $^{11}B\{^1H\}$ MAS spectra.

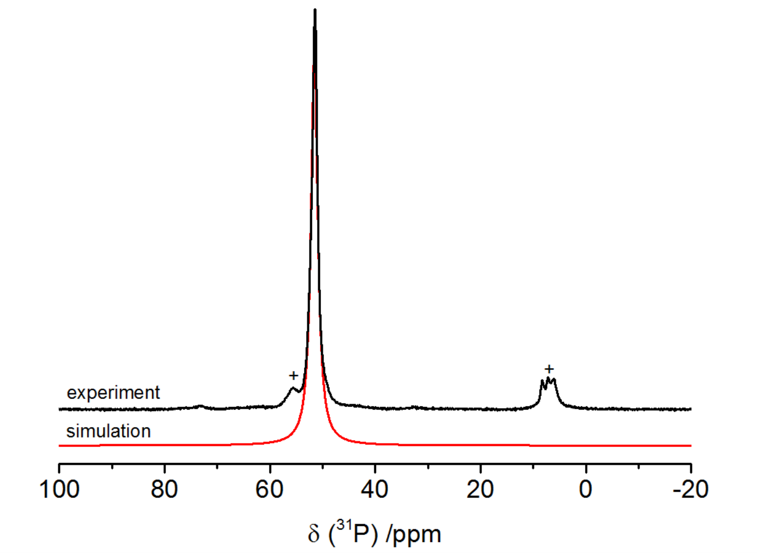

Figure S82. $^{31}P\{^{1}H\}$ CPMAS NMR spectrum (top) of the SO_2 adduct sample (sample from experiment 4.1) measured at 7.05 T with a MAS frequency of 12.0 kHz. Line shape simulation (lower trace) results in $\delta_{iso}(^{31}P)$ = 51.5 ppm. Minor side products are labelled by +.

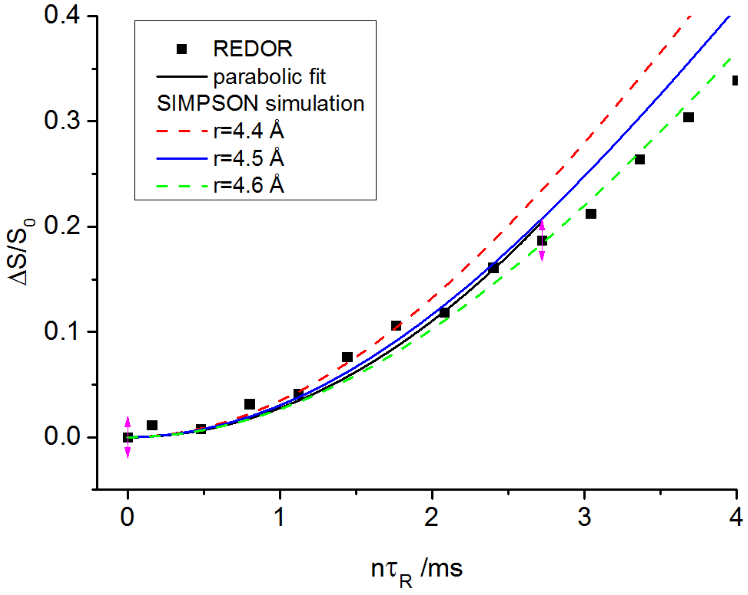

Figure S83. $^{11}B\{^{31}P\}$ REDOR of the SO_2 adduct sample (sample from experiment 4.1) measured at 7.05 T with a MAS frequency of 12.5 kHz. The experimental data are compared with SIMPSON simulations for different assumed P—B distances. The simulation most consistent with the experimental data suggests a boron-phosphorus distance of 4.5 Å (blue curve). Included is also a parabolic fit, following the approach[4] resulting in a second moment value $M_2 = 2.04 \cdot 10^{-5}$ rad^2/s^2 which yields a boron-phosphorus distance of 4.6 Å, assuming a two-spin interaction.

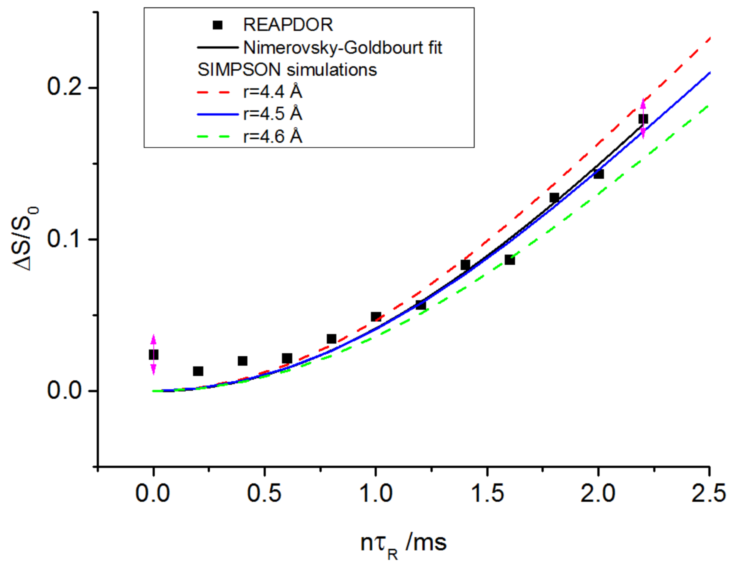

Figure S84. $^{31}P\{^{11}B\}$ CP-REAPDOR of the SO_2 adduct sample (sample from experiment 4.1) measured at 7.05 T with a MAS frequency of 10.0 kHz. Included are SIMPSON simulation for different assumed B--P distances. The simulation most consistent with the experimental data is obtained for a boron-phosphorus distance of 4.5 Å and the fit by the analytical approach[5].

Experimental Section

$^{11}B\{^{1}H\}$ MAS and $^{31}P\{^{1}H\}$ CPMAS NMR spectra were recorded at 7.05 T using a Bruker Avance III 300 spectrometer equipped with a 4 mm MAS-NMR probe, which was operated at a MAS frequency of 12.0 kHz and a nutation frequency of 69.4 kHz. $^{11}B\{^{31}P\}$ rotational echo double resonance (REDOR) were conducted with the standard sequence of Schaefer and Gullion,[6] at the spinning frequency of 12.0 kHz, using 180° recoupling pulses of 10.0 µs length. $^{31}P\{^{11}B\}$ rotational echo adiabatic passage double resonance (REAPDOR) data were measured at a spinning frequency of 12.0 kHz, using a radio frequency power level corresponding to an ^{11}B nutation frequency of 33.7 kHz and a recoupling time of 1/3 of the rotor cyle. Heteronuclear correlation spectra were acquired at 11.7 T with a MAS frequency of 29.8 kHz using a Bruker DSX 500 spectrometer. These spectra were measured using the cross-polarization sequence with Hartmann-Hahn contact times of 800 µs and 7.0 ms for ^{31}P and ^{11}B, respectively. The evolution time of the ^{1}H transverse magnetization was incremented in 50 steps of 33.6 µs. ^{1}H MAS NMR experiments were performed at 20.0 T with a MAS frequency of 60.0 kHz using the EASY background suppression scheme. An empty rotor was measured additionally as described in reference.[7] The BCU temperature was set to 262 K to avoid any frictional heating effects of the sample. ^{1}H chemical shifts were referenced to TMS using adamantane as an internal standard (1.78 ppm). All DFT calculations were performed using the TURBOMOLE program package (version 6.3).[8,9] DFT calculations of ^{1}H chemical shifts were performed on a def2-TZVP[7]/B3-LYP[11,12] level of theory on geometries obtained from unconstrained geometry optimizations on a TPSS-D3[13,14]/def2-TZVP level. ^{1}H chemical shifts were referenced to TMS.

Supplementary Information (Part 3. Computational Section)

Lei Liu[f], Jan Gerit Brandenburg[e,f], Stefan Grimme[f,*]

[e] London Centre for Nanotechnology, University College London, 17-19 Gordon Street London, WC1H 0AH, United Kingdom (computational chemistry)

[f] Mulliken Center for Theoretical Chemistry, Institut für Physikalische und Theoretische Chemie, Universität Bonn, Beringstraße 4, 53115 Bonn,Germany (computational chemistry)

1 Computational details

We closely follow the recently established computational protocol from Ref. 36. The crystal structures were optimized with the composite HF-3c method.[15] The thermostatistical contributions to the Gibbs free energy in the gas phase were obtained from a harmonic oscillator approximation at a temperature of 298.15 K and for 1 atm pressure. HF-3c frequencies are scaled by 0.86 as recommended in the original publication.[16] The geometry optimization and frequency analysis calculations were performed by employing a developer version of the CRYSTAL14 software.[17] This is the ideal software choice for cost-efficient electronic structure calculations as it can exploit all point- and space group symmetries. Accurate electronic energies were obtained from single point calculations at the TPSS[13] and PBE0 level of theory on the HF-3c structures.[18] London dispersion interactions are included with the DFT-D3 dispersion correction in the Becke-Johnson damping scheme including the triple-dipole three-body term. [14,19] A projector augmented plane wave (PAW) basis set[20,21] was used with the energy cut-off of 800 eV. The Brillouin zone was sampled at the Γ point. The single point energy calculations were performed by employing the Vienna Ab initio Simulation Package, VASP 5.4.[22,23] All crystal lattice energies are reported per FLP molecule. The molecular structures were optimized with HF-3c and the TPSS-D3 level of theory,[13,18,19] in conjunction with the def2-TZVP basis set.[10,24] Accurate electronic energies were obtained from single point calculations at the TPSS-D3 and PW6B95-D3 level[25] with a Gaussian type orbital basis set of def2-TZVP quality on the HF-3c structures. The thermostatistical contributions for the molecules were obtained as noted above. The TPSS-D3 frequencies are used unscaled. The density-fitting RI-J[26,27,28] approach for the Coulomb integrals were used to accelerate these molecular calculations which were conducted by the Turbomole 7.0 software.[29] The COSMO-RS (Conductor-like Screening Model for Real Solvents) solvation model[30,31] was used to compute the solvation Gibbs energies by employing the gas–phase optimized structures, and with toluene as the solvent. These calculations were done with the COSMOtherm program.[32] The dispersion corrected density functional tight binding Hamiltonian, DFTB-D3, has been used in its third order variant with self consistent charge redistribution, damping of all hydrogen containing pair potentials by an exponent of 4.2, and 3OB Slater-Koster splines.[33-35] In all periodic DFTB-D3 calculations, the Brillouin zone was sampled at the Γ point. The Born-Oppenheimer molecular dynamics (BO-MD) simulations were performed in the NVT ensemble in which the temperatures were controlled via Nosé–Hoover thermostat.

For more computational details see Ref.[36]

2 Benchmark results

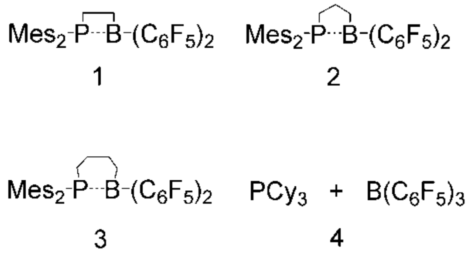

Scheme S13. Four studied frustrated Lewis pairs.

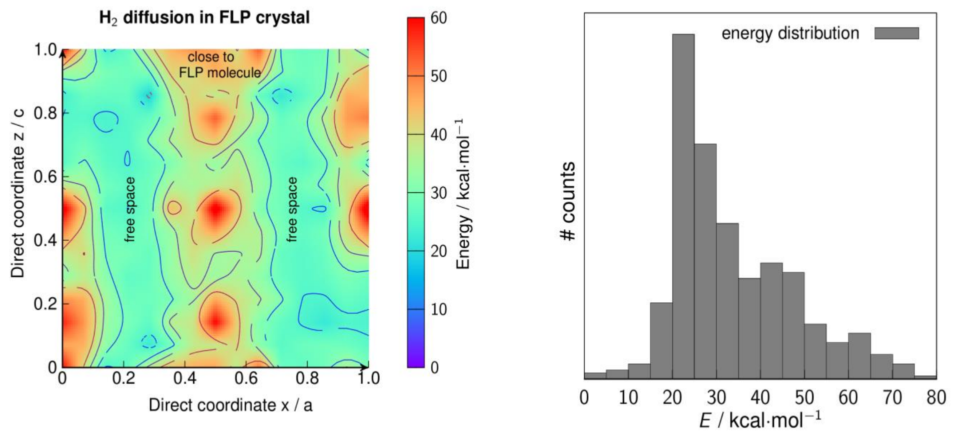

Figure S85. The relative energy of H_2 distributed in the unit cell of FLP **1** calculated at the DFTB-D3 level of theory. The contour plot in the ac-plane of the crystal (a), and the distribution of the relative energies (b).

To understand H_2 mobility in the crystals, we have computed the energies for the H_2 diffusion in the unit cell of FLP **1** via single point calculations at DFTB-D3 level of theory. As shown in Figure S85, one can see that most positions in the unit cell have similar energies denoting a flat potential energy surface. It indicates that H_2 should be able to move inside the bulk. In other words, H_2 can homogeneously distribute in the materials without too high energy barriers. Furthermore, we performed an BO-MD simulation at the DFTB3-D3 level at 300 K for a duration of 3 ps (with a time step of 0.1 fs) to qualitatively examine the H_2 mobility in the crystal structure of FLP **1**. The simulations show that a hydrogen molecule can almost freely move through the channels in the crystal structure driven by the concentration gradient.

Moreover, we have also performed a BO-MD simulation at the DFTB-D3 level of theory with a crystal mass density of ca. 1.0 g cm^{-3} for 15 ps (at 300 K and with a time step of 1 fs) to examine the "liquid" behavior of the intermolecular FLP **4** in the solid state. The results clearly show that the FLP components, in particular, the PCy_3 molecules move quickly from their equilibrium positions into the contact surface thereby diffusing eventually into each other (Figure S86). Hence, Lewis acid and base

components could interact at suitable distances and form solid state FLPs eventually enabling hydrogen splitting locally at contact surfaces.

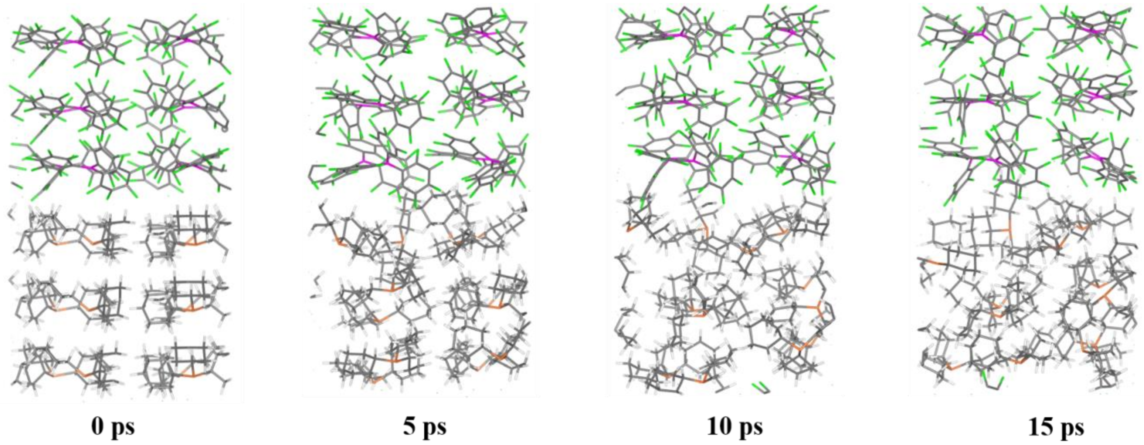

Figure S86. Snapshots of the periodic BO-MD simulation at the DFTB-D3 level of theory for FLP **4,** PCy_3 + $B(C_6F_5)_3$. Color legend: P yellow, B pink, C black, F green and H white.

We have examined the reaction Gibbs free energies for H_2 activation by FLP **1** in the solid state at different level of theory and with different numerical parameter settings (see Table S1). The results show that the reaction Gibbs free energies are independent on the level of theories, and the difference are less than 2 kcal mol^{-1}.

Table S1. Calculated reaction Gibbs free energies for H_2 activation by FLP **1** in the solid state at different level of theory and with different numerical parameter settings. All geometries were optimized at HF-3c level, the thermostatistical contributions were calculated at HF-3c level, and the single point energy calculations were performed with the same basis set (PAW). All values are given in kcal mol^{-1}.

	$+H_2$	$+2H_2$	$+3H_2$	$+4H_2$
TPSS-D3[a]	0.5	-0.6	-0.5	1.0
TPSS-D3[b]	0.9	0.0	0.4	2.2
TPSS-D3[c]	0.8	0.0	0.3	2.2
TPSS-D3[d]	0.8	-0.1	0.3	2.3
PBE0-D3[a]	0.6	-0.9	-0.9	0.2

[a] 1×1×1 k points and 800 eV energy cutoff; [b] 1×1×1 k points and 900 eV energy cutoff, [c] 2×1×1 k points and 900 eV energy cutoff, [d] 2×2×1 k points and 900 eV energy cutoff. $+xH_2$ denote we added one, two, three and four hydrogen molecules per unit cell.

Table S2. Calculated reaction Gibbs energies and the individual components for the H_2 activation by FLP **1** in solution and in solid state. All values are in given kcal mol^{-1}.

	ΔE	ΔG_{RRHO}	$\Delta\delta G_{sol}$	ΔG_{total}
Solution[a]	-6.2	11.5	-8.8	-3.5
Solution[b]	-4.7	11.5	-8.8	-2.0
Solid state[c]	-8.6	9.6	---	1.0

[a]TPSS-D3/def2-TZVP//HF-3c, COSMO-RS(toluene)
[b]PW6B95-D3/def2-TZVP//HF-3c, COSMO-RS(toluene)
[c]TPSS-D3/PAW//HF-3c

The direct crystal structure of FLP **4** is experimentally not available. We started from the crystal structure of **5** by removing the SO_2 species and optimized the structure at HF-3c level of theory. The calculated unit cell parameters are provided in Table **S3**. We compared the single point energies between TPSS-D3/def2-TZVP and PW6B95-D3/def2-TZVP level of theory, and the results are summarized in Table **S4** and **S5**. We can see that the differences are rather small, which are less than 1 kcal mol^{-1}.

Table S3. Calculated unit cell parameters for FLP **4**.

	Unit cell parameters (Å)			
	a	b	c	density (g/cm^3)
reactant	10.29	10.34	21.28	1.18
product	10.91	10.99	20.04	1.21

Table S4. Calculated reaction Gibbs free energies and the individual components for the H_2 activation by FLP **4a** in the solution and in the solid state. All values are given in kcal mol^{-1}.

	ΔE	ΔG_{RRHO}	$\Delta\delta G_{sol}$	ΔG_{total}
Solution[a]	-11.8	7.4	-11.0	-15.5
Solution[b]	-11.1	7.4	-11.0	-14.8
Solid state[c]	-25.1	5.4	---	-19.7

[a]TPSS-D3/def2-TZVP//HF-3c, COSMO-RS(toluene)
[b]PW6B95-D3/def2-TZVP//HF-3c, COSMO-RS(toluene)
[c]TPSS-D3/PAW//HF-3c

Table S5. Calculated reaction Gibbs energies and the individual components for the formation of **3a** from FLP **4a** in the solution and in the solid state. All values are given in kcal mol^{-1}.

	ΔE	ΔG_{RRHO}	$\Delta\delta G_{sol}$	ΔG_{total}
Solution[a]	-31.4	-4.7	-14.6	-50.7
Solution[b]	-32.0	-4.7	-14.6	-51.2
Solid state[c]	-45.4	-0.4	---	-45.8

[a]TPSS-D3/def2-TZVP//HF-3c, COSMO-RS(toluene)
[b]PW6B95-D3/def2-TZVP//HF-3c, COSMO-RS(toluene)
[c]TPSS-D3/PAW//HF-3c

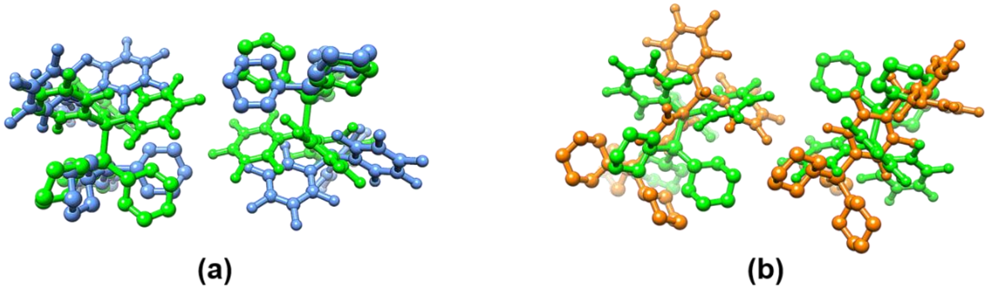

Figure S87. Overlays of HF-3c calculated crystal structures of $PCy_3/B(C_6F_5)_3$ (green) and $[HPCy_3^+][HB(C_6F_5)_3^-]$ (blue) (a), and crystal structures of $PCy_3/B(C_6F_5)_3$ (green) and the S_{N2Ar} product **3a** (orange) (b). Hydrogen atoms except P-H and B-H are omitted for clarity .

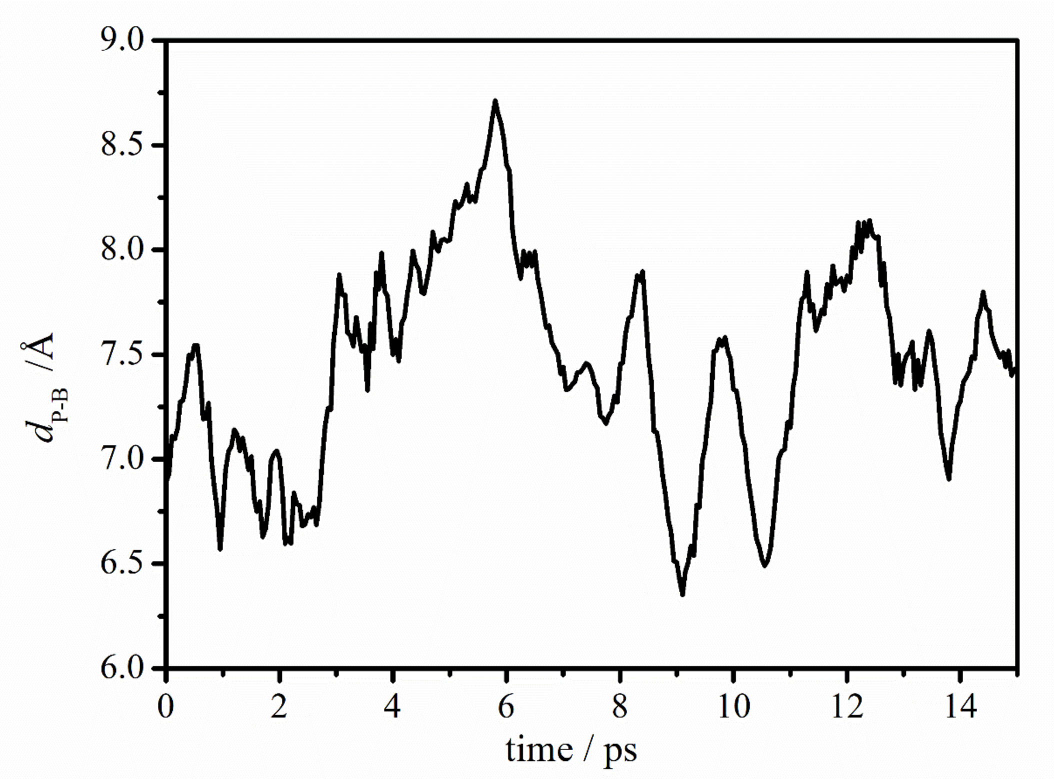

Figure S88. The distance between P and B (d_{PB}) along the MD simulations.

References

1. P. Spies, S. Schwendemann, S. Lange, G. Kehr, R. Fröhlich and G. Erker, *Angew. Chem. Int. Ed.*, 2008, **47**, 7543-7546.
2. Y. Jiang, B. Schirmer, O. Blacque, T. Fox, S. Grimme and H. Berke, *J. Am. Chem. Soc.*, 2013, **135**, 4088-4102.
3. G. C. Welch, L. Cabrera, P. A. Chase, E. Hollink, J. D. Masuda, P. Wei and D. W. Stephan, *Dalton Trans.*, 2007, 3407-3414.
4. M. Bertmer and H. Eckert, *Solid State Nucl. Magn. Reson.*, 1999, **15**, 139-152.
5. E. Nimerovsky and A. Goldbourt, *Phys. Chem. Chem. Phys.*, 2012, **14**, 13437-13443.
6. J. Schaefer, *J.Magn.Reson.*, 2011, **213**, 421-422.
7. C. Jaeger and F. Hemmann, *Solid State Nucl.Magn.Reson.*, 2014, **57-58**, 22-28.
8. R. Ahlrichs, F. Furche and C. Hättig, TURBOMOLE, version 6.3, Universität Karlsruhe, 2009.
9. R. Ahlrichs, M. Bär, M. Häser, H. Horn and C. Kölmel, *Chem. Phys. Lett.*, 1989, **162**, 165-169.
10. F. Weigend and R. Ahlrichs, *Phys. Chem. Chem. Phys.*, 2005, **7**, 3297-3305.
11. A. D. Becke, *J. Chem. Phys.*, 1993, **98**, 5648-5652.
12. P. J. Stephens, F. J. Devlin, C. F. Chabalowski and M. J. Frisch, *J. Phys. Chem.*, 1994, **98**, 11623-11627.
13. J. Tao, J. P. Perdew, V. N. Staroverov and G. E. Scuseria, *Phys. Rev. Lett.*, 2003, **91**, 146401.
14. S. Grimme, S. Ehrlich and L. Goerigk, *J. Comput. Chem.*, 2011, **32**, 1456-1465.
15. R. Sure and S. Grimme, *J. Comput. Chem.*, 2013, **34**, 1672-1685.
16. M. Cutini, B. Civalleri, M. Corno, R. Orlando, J. G. Brandenburg, L. Maschio and P. Ugliengo, *J.Chem.Theory Comput.*, 2016, **12**, 3340-3352.
17. R. Dovesi, R. Orlando, A. Erba, C. M. Zicovich-Wilson, B. Civalleri, S. Casassa, L. Maschio, M. Ferrabone, M. De La Pierre, P. D'Arco, Y. Noël, M. Causà, M. Rérat and B. Kirtman, *Int.J.Quantum Chem.*, 2014, **114**, 1287-1317.
18. C. Adamo and V. Barone, *J. Chem. Phys.*, 1999, **110**, 6158-6170.
19. S. Grimme, J. Antony, S. Ehrlich and H. Krieg, *J. Chem. Phys.*, 2010, **132**, 154104.
20. P. E. Blöchl, *Phys. Rev. B*, 1994, **50**, 17953-17979.
21. G. Kresse and D. Joubert, *Phys. Rev.B*, 1999, **59**, 1758-1775.
22. G. Kresse and J. Hafner, *Phys.Rev.B*, 1993, **47**, 558-561.
23. G. Kresse and J. Furthmüller, *Comput.Mater.Sci.*, 1996, **6**, 15-50.
24. F. Weigend, *Phys. Chem. Chem. Phys.*, 2006, **8**, 1057-1065.
25. Y. Zhao and D. G. Truhlar, *J. Phys. Chem. A*, 2005, **109**, 5656-5667.
26. S. Grimme, *Chem. Eur. J.*, 2012, **18**, 9955-9964.
27. K. Eichkorn, O. Treutler, H. Öhm, M. Häser and R. Ahlrichs, *Chem.Phys. Lett.*, 1995, **240**, 283-290.
28. K. Eichkorn, F. Weigend, O. Treutler and R. Ahlrichs, *Theor.Chem.Acc.*, 1997, **97**, 119-124.
29. F. Furche, R. Ahlrichs, C. Hättig, W. Klopper, M. Sierka and F. Weigend, *Wiley Interdiscip. Rev.: Comput.Mol.Sci.*, 2014, **4**, 91-100.
30. A. Klamt and G. Schuurmann, *J. Chem. Soc., Perkin Trans. 2*, 1993, 799-805.
31. A. Klamt, *J. Phys. Chem.*, 1995, **99**, 2224-2235.
32. F. Eckert and A. Klamt, *COSMOlogic GmbH & Co. KG, Leverkusen, Germany*, 2010.
33. M. Elstner, D. Porezag, G. Jungnickel, J. Elsner, M. Haugk, T. Frauenheim, S. Suhai and G. Seifert, *Phys.Rev. B*, 1998, **58**, 7260-7268.
34. M. Gaus, A. Goez and M. Elstner, *J. Chem. Theory Comput.*, 2013, **9**, 338-354.
35. J. G. Brandenburg and S. Grimme, *J. Phys. Chem. Lett.*, 2014, **5**, 1785-1789.
36. L. Liu, J. G. Brandenburg and S. Grimme, *Philos. Trans. A Math. Phys. Eng. Sci.*, 2017, **375**, 20170006.
